# Supplementary material for: Qualitative exploration of comprehension and experiences of healthcare professionals regarding nutrition care in Karachi, Pakistan
Source: PLOS Glob Public Health. 2025 Dec 30;5(12):e0005483. doi: 10.1371/journal.pgph.0005483 (PMC12753000; doi:10.1371/journal.pgph.0005483)
Supplement: S1 File — (DOCX) [file pgph.0005483.s001.docx]

**S1 File: Coding tree: Highlights the main themes, subthemes and their description**

| **Themes** | **Sub themes** | **Description** |
| --- | --- | --- |
| Filling gaps beyond their responsibilities |  | To identify and explore the understanding and perception of healthcare providers about nutrition care. |
| Compelled to care despite several bottlenecks | - Role variability between public and private healthcare settings - Communication barriers - Lack of in-depth knowledge regarding nutrition care - Financial constraints - Lack of health literacy - Lack of networking and collaboration | To understand and explore the role and challenges faced by healthcare professionals. |
| Disseminating awareness about nutrition care | - Integrated curriculum for nutrition education and training - Establishing a multidisciplinary collaboration and referral system - **Spreading information and** stakeholders’ engagement | To explore participants’ opinions about the strategies to improve nutrition care |
