# Supplementary material for: Qualitative exploration of comprehension and experiences of healthcare professionals regarding nutrition care in Karachi, Pakistan
Source: PLOS Glob Public Health. 2025 Dec 30;5(12):e0005483. doi: 10.1371/journal.pgph.0005483 (PMC12753000; doi:10.1371/journal.pgph.0005483)
Supplement: S3 File — (DOCX) [file pgph.0005483.s003.docx]

**S3 File. Semi structured interview guide questions for healthcare professionals**

|  | What do you understand or perceived by the term nutrition care? |
| --- | --- |
|  | Have you ever given or contributed to nutrition care to any person? If yes to whom and how? (Probe: individual nutrition care, population nutrition care) |
|  | What is your opinion or experience regarding nutrition care provision? (Probe: role and responsibilities, dietitian’s attributes) |
|  | What in your experience are the difficulties or challenges that you and your coworkers face in the delivery of nutrition care? |
|  | What in your experience are the changes needed or suggestions that can facilitate or improve nutrition care? |
