# Supplementary material for: Qualitative exploration of comprehension and experiences of healthcare professionals regarding nutrition care in Karachi, Pakistan
Source: PLOS Glob Public Health. 2025 Dec 30;5(12):e0005483. doi: 10.1371/journal.pgph.0005483 (PMC12753000; doi:10.1371/journal.pgph.0005483)
Supplement: S4 File — (DOCX) [file pgph.0005483.s004.docx]

**S4 File: Thematic analysis framework**

| **Phases** | **Description** |
| --- | --- |
| 1. **Data Familiarization** | Read interview transcripts and field notes multiple times, focusing on predetermined areas (e.g., barriers, facilitators, and strategies in nutrition care delivery). |
| 1. **Codes Generation** | Apply structured code guided by research objectives (e.g., communication barriers, limited nutrition knowledge, financial constraints, lack of collaboration). |
| 1. **Themes Generation** | Organize codes into broader, theory-driven themes such as Systemic Barriers, Professional Gaps, and Awareness Strategies. |
| 1. **Reviewing Themes** | Cross-check that themes comprehensively represent coded extracts and the full dataset, ensuring consistency with nutrition-related priorities (e.g., training, referral systems, interdisciplinary teamwork). |
| 1. **Defining and Naming Themes** | Refine and finalize theme names (e.g., Filling Gaps Beyond Responsibilities, Compelled to Care Despite Bottlenecks, Disseminating Awareness). |
| 1. **Producing the Report** | A structured report using illustrative quotes was developed. Link findings to existing nutrition care guidelines, public health nutrition literature, and policy implications for Pakistan. |
