# Supplementary material for: Qualitative exploration of comprehension and experiences of healthcare professionals regarding nutrition care in Karachi, Pakistan
Source: PLOS Glob Public Health. 2025 Dec 30;5(12):e0005483. doi: 10.1371/journal.pgph.0005483 (PMC12753000; doi:10.1371/journal.pgph.0005483)
Supplement: S5 File — (ZIP) [file pgph.0005483.s005.zip › Doctor Female -002.pdf]

[redacted]

N: اسلام و علیکم میرا نام نفیسہ
طاہرہ سید ہے اور میرا تعلق
ضیاء الدین یونیورسٹی سے ہے۔
اور میں جو ہیوں ہم لوگ
ریسرچ کر رہے ہیں کوالیٹیٹو
ریسرچ ہے ہماری

R: ہمم (ہم)

N: جس کے اندر جو ہے ہیلتھ
کیئر پروفیشنلز سے نیوٹریشن
کنسرنڈ ریلیٹڈ کیا انکی
اوپینین (opinions) ہیں
کیا انکے خیالات ہیں یا کیا جو
تے نا انکی تھوٹس (thoughts)
ہیں وہ جو ہیں نا ہم جاننا
چاہ رہے ہیں۔ تو..... کیا
ہوتے ہیں ہم آپکا آپ سے
کچھ کوسچنز (Questions) وغیرہ
پوچھیں گے اور آپ آپ
کے پاس رائٹ (Right)
ہے کہ آپ چاہیں کسی
بھی کوسچن (Question)
کا جواب دیں یا جسکا چاہے
آپ نہ جواب دیں
جس کا جواب نہیں دیں گی
اس کا انٹرویو (Interview)
پہ کوئی اثر نہیں پڑے
گا اور

گا۔ ٹھیک ہے؟ اور اگر آپ چاہتیں
ہیں کہ کسی بھی وقت آپ
انٹرویو (Interview) کو
ختم کریں تو آپ ختم کر سکتیں
ہیں! اور آپ جو بھی معلومات
دیں گیں تو وہ آپ کی جو
معلومات ہیں صرف ریسرچ
پرپز (Research Purpose) کے لیے
یوز (Use) ہونگی اور جب اس
کو اینالیسز (Analysis) کے لیے دے...
اینالائز کریں گے اس ریسرچ (Research)
کو تو آپ کا نام اور کوئی بھی
جو آپ کی آئیڈینٹیفیکیشن
(Identification) ہے وہ اس
میں سے ریموو (Remove)
کر دیں گے۔ اور جو بھی معلومات
مجھے ملیں گی اس کو
پبلش کرتے ہوئے کریں گے
مگر پبلش کے ٹائم (Time) پر
آپ کی کوئی امپورٹنٹ
(Important) جو آپ کی
معلومات ہیں وہ شیئر (Share)
نہیں ہونگی اور کمپلیٹ
(Complete) جو ہے نا اس کے اندر
آپ کے اینونیمس (Anonymous)
رکھا جائے گا۔ ~~اور~~

ہم...

N اور آر اور آپ اس تمام کنورسیشنز (conversations) کو اگری (Agree) کرتی ہیں۔ تو آپ اپنا وربلی (verbally) (Confi..) ٹھیک ہے نا ہمیں کونفی.... آ..... کونسینٹ (consent) دے دیجیے پلیز (Please)

R اچھا وعلیکم اسلام میرا نام ڈاکٹر (Doctor) [redacted] ہے۔ اور میں بالکل کنسینٹ (consent) دے رہی ہوں آپ کو جو سوال کرنا ہے وہ کریں۔

N ٹھیک ہے... اچھا آپ پہلے اپنے بارے میں بتائیں کیا ملازمت کرتی ہیں اور کیا آپ کی ریسپونسیبلیٹیز ہیں (Responsibilities) ہیں تو جی

R ٹھیک ہے اچھا میں نے نام تو بتا دیا کہ میرا نام [redacted] ہے اور میں نے جناح میڈیکل اینڈ ڈینٹل کالج سے MBBS کیا ہے۔ اس کے بعد میں نے JPMC سے ہاؤس جوب (House Job) کی ہے۔ ہاؤس جوب (House Job) کے بعد فی الحال ابھی میرا آگے ایمرجنسی میڈیکل

R (Pursue) پرسو (Emergency Medicine)
کرنے کا ارادہ ہے، تو اس کا
ایلیجیبلٹی ایگزیم (Exam) میں نے
(P ٹائٹل) پارٹ ون (One) دیا
ہوا ہے۔ جیسے کو MI CAMP I ?
بولتے ہیں وہ میں دیا ہوا ہے۔
[redacted] اور ابھی میں
(As an) ہسپتال میں ایز این
RMO کام کر رہی ہوں۔ تو
(Basically) بیسکلی میری ڈیوٹیز
(duties) جو ہوتی ہیں وہ وارڈ
میں بھی ہوتی ہیں کبھی جنرل
پرائیویٹ میں بھی ہوتی ہیں،
کبھی ICU میں ہوتی ہیں اور
کبھی ER میں بھی ہوتی ہیں
تو ڈیپینڈ (depend) کرتا
ہے باقی (as a whole) ایز
آ ہول زیادہ (Interested) انٹرسٹڈ
میں ICU میں ہوتی ہوں
یا پھر ایمرجنسی میں ہوتی
ہوں۔

N ٹھیک ہے۔ تو آپ کو جب کام
اتنا کر رہی ہوتی ہیں تو آپ کے
اندر آپ کا کبھی تعلق ہوتا
ہے کہ جو کسی کو (Nutrition)
نیوٹریشن کے حوالے سے کوئی
معلومات دیں یا کاؤنسلنگ

W T F S S Date

Ent - کمرشل (Counselling)
اس سلسلے میں -

بالکل ایسے بہت سارے مریض ہوتے ہیں جو بہت کومن (common) ہوتے ہیں۔ جیسے ڈائیبیٹیز پیشنٹس (diabetes) ہوتے ہیں تو ان کو (Patient) کاؤنسلنگ (Councelling) بہت دفعہ کرنی ہوتی ہے کہ کیا کھانا ہوتا ہے کس طریقے سے کھانا ہے کتنا فروٹس (fruits) آپ لے سکتے ہیں کیا [illegible] آپ لے سکتے ہیں۔ لائک (Like) کمپلیٹ ڈائٹ فار اے ڈائیبیٹیز پیشنٹ ((Complete diet for diabetes patient).

تو اس میں میں نے کافی دفعہ صاف نیوٹریشن کے حوالے سے بات کی ہے اور اس کے علاوہ میرا نہیں خیال کہ کبھی اس ایریا میں اتنا ایکسپوژر (Exposure) ہوا ہے کیوں کہ (Mostly) ہم لوگ (Superficially) کام کر رہے ہوتے ہیں کہ یہ یا یہ چیز کھا لیں یہ کر لیں یا پھر for e.g فار

ایگزامپل (کوئی) اسٹروک (Stroke)
کا پیشنٹ ہے تو میکسیمم
(Maximum) یعنی جس آرڈر میں ہوتے
ہیں کہ (Ensure) دے دیا جا
(Glucerna) دے دیا (4) تو اچھا
اس سے پہلے میں نے (NMC)
میں کام کیا ہے اور وہاں پہ کوئی
نیوٹریشنسٹ (Nutritionist) والا
رول (Role) میں نے کبھی نہیں
دیکھا۔ جو ڈاکٹرز (Doctors)
تھے وہی اپنے سلسلے سے
بتا دیتے تھے کہ ہاں یہ کھالیں
یہ کھالیں۔ یہ فیز جو ہے میں
نے ہاشمانیز (Hashmanis) میں جا کے
دیکھی ہے کہ پراپر (Proper)
نیوٹریشنسٹ (Nutritionist) ہوتی
ہے جو ڈائٹ (Diet) بتاتی
ہیں۔

N یعنی آپ کا اس سلسلے میں
کیا رول (Role) ہوتا ہے؟
کیا آپ کیا ایڈوائز (Advice)
کرتی ہیں؟

R ٹھیک ہے۔ اچھا ہمارا یہاں پہ آنے
کے بعد تو کوئی خاص رول (Role)
نہیں ہے۔ نیوٹریشن کے حوالے
سے ہم لازمی ضرور ~~[illegible]~~ کرتے
ہیں۔

[redacted] ہمارے یہاں
ہوتی ہے جو نیوٹریشنسٹ
(Nutritionist) ہیں - وہ پوچھتی
ہیں اکثر کہ میں اس کو یہ
کرا دوں، یا یہ چیز دے
دوں - تو ایک (Suggestion)
سجیشن آرڈر دیتے ہیں -
لیکن ایز سچ (as such) کوئی
کمپلیٹ (Complete) نیوٹریشنسٹ
جو ایک طریقے سے ایڈوائس
جیسے دیتی ہے یا ڈائٹ
جو پلان (plan) کرتی ہے وہ
آبویسلی (Obviously) نہیں
نہیں کرتے تو ہمارا جو
بیسک مینجمنٹ پلان
(basic management plan)
جو ڈائٹ (Diet) کے حوالے
سے جو ہوتا ہے وہ ویسے
ہوتا ہے کہ (for e.g) فار
ایگزامپل ہم نے شوگر فری
ڈائٹ کا بول دیا یا بیکری
آئیٹمز منع کر دیے اگر اسٹروک
(Stroke) والا پیشنٹ ہے تو
کوئی ملک (Milk) سجیسٹ
Glucerna کر دیا یا (Suggest)
سجیسٹ کر دیا (Suggest)
اگر (Diabetic) ڈائیبیٹک
ہے تو کوئی بیسک (Basic) -

ہے اتنا ڈیپتھ (Depth) میں نہیں جاتے ڈاکٹرز (Doctors) مطلب حوالے سے ایز آ ڈاکٹر (as a doctor). نہ میں نے کبھی پریکٹس (Practice) کی ہے ~~جیسے~~ کبھی اور میں نے کبھی ڈاکٹری کی تعلیم لی ہے ایسپیشلی (especially) پاکستان میں

N مطلب یہ کہ اگر آپ سے کوئی رائے معلوم کرے کہ جیسے پیشنٹ بعض دفعہ پوچھ بھی رہے ہوتے ہیں کہ کیا کھانا چاہیے کیا نہیں۔ تو کیا آپ اس کے بارے میں ان کو کوئی (Suggestions) سجیشن یا رائے دیتی ہیں۔

R بالکل، بالکل۔ اگر مریض کے پوچھ رہا ہے کہ کیا کھا سکتے ہیں، کیا نہیں کھا سکتے تو ہمیں جتنی نولیج (Knowledge) ہوتی ہے اس حساب سے ایڈوائس (Advise) دے رہے ہیں۔ دین اگین (Then again) ہماری جو نولیج (knowledge) ہے اس چیز پہ وہ بہت سپرفیشل ~~ہے~~ (Superficial) ہے۔ مطلب اتنی ڈیپتھ (depth) میں نہیں ہے۔

کہ یہ گیہوں دے سکتے ہیں یا یہ
گیہوں نہیں دے سکتے کیونکہ یہ
فار ایگزیمپل (For example) سیلیک (Celiac)
سیلیک ڈیزیز (Disease)
لہٰذا اس کا ہمیں پتہ ہے کہ یہ
لیکٹوز انٹولرنٹ (Lactose
Intolerant) ہو گا تو اسکو
ملک (Milk) نہیں دینا ہے۔ اسلئے
یہ چیزیں ہیں جیسے وغیرہ نہیں دینی
لیکن بعد میں بات ہے ہمیں نہیں
پتہ کہ جو چیز دے سکتے ہیں
وہ کتنی کوانٹٹی (Quantity)
میں دے سکتے ہیں۔ تو بہت
سوپر فیشل (Superficial)
نولیج (Knowledge) ہے
ریگارڈنگ (Regarding) نیوٹریشن
اینڈ ڈائٹ (Nutrition / Diet)
لیکن یہ بیسکس (Basics) سے
ہیں۔ ~~اگر کوئی~~ اگر کوئی مریض
پوچھے کہ مجھے شوگر ہے میں
کیا کھا سکتا ہوں تو مجھے پتہ
ہے کہ دن میں ایک پھل دن
میں کھا سکتے ہیں یا بیکری
آئٹم نہیں کھا سکتے تو ہاں
پیشنٹ اگر پوچھ رہا ہے تو
گائیڈ (guide) کر سکتے ہیں لیکن
پوری جو ڈائٹ چارٹنگ
(Diet charting) ہوتی ہے کہ آپ
گھر میں کیا کھائیں گے پورے

مہینہ (Month) کی کیا پلان ہوتی
پھر وہ ہم بھی کر پاتے -

N: ٹھیک ہے نہ پھر وہ آپ کے پاس
~~نیوٹریشن~~ نیوٹریشنسٹ کرتی
ہے وہ کام ۔

R: جی ۔ وہ بالکل ۔

N: ٹھیک ہے ۔ تو کبھی ایسا ہوا
کہ آپکو اس چیز کا موقع ملا
کہ آپ کس کمیونٹی میں کہ
گروپس (Groups) میں تو گئی
ہوں نیوٹریشن کیئر (Nutrition Care)
کے حوالے سے ان لوگوں ~~سے~~
سیشن دی ہو یا کاؤنسلنگ
کی ہو یا انکو کوئی پریزنٹیشن
دی ہو -

R: کمیونٹی، اس میں تو کلوز
فرینڈز ~~[illegible]~~ اینڈ فیملی بھی
آجاتی ہے پھر تو ~~[illegible]~~ ایسا ہے

N: اچھا جی

R: میرے فادر (Father) ہیں
وہ جیسے اسٹروک (Stroke)
پیشنٹ (Patient) ~~[illegible]~~ بھی
تھے پہلے اب تو خیر ٹھیک ہیں

اور پھر وہ ذیابیطک (Diabetic)
بھی ہیں تو ان کی بھی کمپلیکیشنز
(Complications) بھی زیادہ ہوتی ہے
زیادہ تر تو میں کہ آپ یہ نہیں کرنا
وہ نہیں کرنا ہے بس آپ نے سیم
ایک فروٹ کھانا ہے بیکری آئٹم
نہیں کھانے، میٹھا نہیں کھانا،
چائے میں چینی نہیں پینی تو
یہ چیزیں ہیں باقی یہاں (وہ
Such) کیسی فیملی (family) میں
زیادہ تر کوئی پوچھتا بھی ہے
تو وہ شوگر کے حوالے سے پوچھتا ہے
اور یہاں ڈسکشن وغیرہ ہو جائیں
کسی کو جو وہ پوچھ رہے ہوتے
ہیں کہ یہ کیا کھائیں تو ان چیزوں
کو یہ تو (Obviously) آبویسلی
گائیڈ کرنا چاہیے اور گائیڈ کرتے
بھی ہیں۔

N ٹھیک ہے ٹھیک ہے۔ مطلب
ایز سچ (As such) لوگ فیملی
ممبرز میں سے کوئی پوچھ رہا ہو
تو آپ ان کو ضرور سے گائیڈ
کر دیتے ہیں۔

ع بالکل۔ بالکل۔

N اور زیادہ جو ہوتا ہے کمیونٹی
بیسڈ۔

(Community Based) جو پروگریمز ہوتے ہیں۔

P اس میں کبھی موقع نہیں ملا مطلب (As such) کبھی نہیں ہوا کہ کمیونٹی میں جس طرح آپ پوچھ رہے ہیں ویسا کبھی ہوا ہو۔ بات انڈیویجول (Individual) لوگ پوچھتے ہیں تو (Obviously) آبویسلی انکا گائیڈ (Guide) کرتے ہیں لیکن As such نہیں۔

N کبھی گروپ میں موقع نہیں ملا۔ ٹھیک ہے۔ یہ جو آپ کی جو ساری ریسپونسبلیٹیز ہیں (Responsibilities) ہیں، آپ کے کیا آپ ایمرجنسی (Emergency) میں ہوتی ہیں تو ہر طرف ٹارگٹ (Target) شیڈول (Schedule) ہوتا ہے تو آپ کے خیال سے آپ کو کیا مشکلات پیش آرہی ہوتی ہیں یا چیلنجز (Challenges) پیش آرہے ہوتے ہیں۔ جب آپ سارے پیشنٹس (Patients) کو بھی ڈیل (Deal) کر رہے ہوتے ہیں اور ڈیل رہے ہوتے ہیں نیوٹریشن (Nutrition) کیئر (Care) کے حوالے سے غذائی صحت کے حوالے سے۔ کون

مشکلات یا پریشانیاں ہیں اور جو
ہوتی ہیں مجھے کہ بات سمجھ نہیں
آرہی تو آپ جو سمجھا رہے ہیں کہ
کیا ہو رہا ہے۔

e
انیشلی (Initially) ہمارے پاس
ER میں جو پیشنٹس آرہے ہوتے ہیں وہ
ڈائریہ (Diarrhea) اور وومٹنگ (Vomiting)
کے ساتھ ایسپیشلی (Specially)
[redacted] میں تو یہ جو بہت زیادہ
ہوتی ہے تو ڈائریہ اور وومٹنگ
والے پیشنٹ تو پوچھتے ہیں کہ ہم
کیا کھائیں۔ تو ہم [illegible]
ہر ایک مسئلہ ہوتا ہے کیونکہ
وہ کہتے ہیں کہ (اگر ہم سے ہم انہیں
پھر کہتے ہیں کہ کیلا کھائیں، تو کہتے ہیں
نہیں ڈاکٹر صاحب، ہم کیلے سے تو پھر
ہمارا سینہ خراب ہو جاتا ہے۔ اور
اس طرح بھی کنفیوز ہو جاتے ہیں کہ
کیلا کھانا ہے کہ نہیں کھانا ہے۔ ہوں
کہ اوبویسلی (Obviously) وہ چیسٹ
کنجیشن (Chest Congestion) کرتا ہے
لیکن پھر بھی یہ ساری چیزیں میڈیکلی
پروون (Medically Proven) نہیں ہیں کہ
کیلا (Banana) کھانے سے جو ہے chest
کنجیشن (Congestion) ہو رہا ہے یا چاول سے
کنجیشن ہو رہا ہے۔ یہ صرف باتیں
ہیں جو ہم میں منتقل ہوتے آرہے ہیں۔

تو اس عمر میں مجھے ڈفیکلٹی
(Difficulty) ہوتی ہے مشکل اور بات
اسٹروک (Stroke) کے پیشنٹس
(Patients) ہوتے ہیں یا (Paediatrics)
پیڈیاٹرک کچھ گروپ ہے ہمارے
پاس بلکل اولڈ (Old) ایج (age)
والے جو پیشنٹس [illegible] ہیں
60 ایئرز کے اوپر ہوتے ہیں یا 70
ایئرز کے اوپر ہوتے ہیں وہ کمپلیٹلی
(Completely) جس کو ہم کہتے ہیں
کیکیکٹک (Cachectic) لُک ہوتے
ہیں جیسے کینسر پیشنٹس والا لُک
(Look) ہوتا ہے بوڈی میں کوئی
ماس نہیں ہے صرف ہڈی سٹرکچر
ہوتا ہے تو انکو ذرا سمجھانے
میں ٹائم لگتا ہے ایک تو کنڈیشن
انکا جو معدہ ہوتا ہے
صحیح سے کام نہیں کر رہا ہوتا اور
وہ سمجھنے کو بھی تیار نہیں
ہوتے کہ کیا نہیں کھا سکتے -
پھر ER میں اور پیشنٹس (Patients)
آئے ہوتے ہیں ہارٹ (Heart Burn)
برن والے جن کو ~~[illegible]~~
معدہ میں جلن ہو رہی ہے،
ان کو کاؤنسلنگ (Counselling)
میں کافی ٹائم لگتا ہے کہ بھائی مرچ
والی چیزیں نہیں کھانی تو اب
چائے نہیں پینی، تو مشکلات
تو ہوتی ہیں لیکن آئی تھنک

I (I think) ایز آ نیشن (as a nation) پاکستان میں جو عوام ہے
ان کو خود بھی نہیں پتہ ہوتا کہ
کیا کھانا ہے کیا نہیں کھانا اور ایز
آ ڈاکٹر (As a doctor) ہمارا جو
نولیج (knowledge) بھی بہت
کم ہے اس حوالے سے کیونکہ ہم اتنے
زیادہ انوولڈ (Involved) ہوتے
ہیں ان چیزوں میں میڈیسن
میں (Medicine) میں کہ اس چیز پہ
کبھی فوکس (focus) نہیں کرتے
تو آتے جاتے پیشنٹ بالکل پیشنٹ
پوچھتے ہیں کیا کھائیں میکسیمم تو اگر
ہو سینہ خراب ہے ہم انہیں بس
یہ بتا دیتے ہیں کہ تلا ہوا نہیں کھانا
فاسٹ فوڈ نہیں کھانے، لیکن اگر اتنی
لمیٹڈ (Limited) سورسز (Sources) آف نولیج
ہیں کہ نہیں پتہ، جو پتہ چلا وہ
اس انہوں نے بتا دیا مجھے بھی تھے
تو یہ نہیں کھانا یہ نہیں کرنا بس
یوں ہی سے جو پتا ہے۔ سو
آئی تھنک سورسز آف نولیج
(Source of knowledge) جو ہے وہ
بہت کم ہے ڈاکٹرز (Doctors) کے
پاس اس حوالے سے تو یہی چیزیں
آئی ہیں۔

N تو آپ کی رائے کیا ہے جو یہ ہم
اس کو بہتر بنانے کے لیے (Improve) کرنے
کے لئے کیا کر سکتے ہیں؟ 1

R: کیا کر سکتے ہیں؟ ایز آ ڈاکٹر (As a Doctor) والے لیول پہ؟

N: جی آپ کے لیول پہ جو آپ کام کر رہے ہیں اس کے حساب سے کیا چیزیں آپ کر سکتے ہیں؟

R: آئی تھنک (I think) اب تو خیر بہت آسانی ہو جاتی کہ یہ نیوٹریشنسٹ (Nutritionist) ہے اگر دیں آئی تھنک پر سیمینار میں تو ہونے جا رہے۔ اور ایک سیمینار ہو جائے یا کبھی جیسے کوئی کانفرنس ہو جاتی ہے تو اس سے تھوڑا نالج (Knowledge) تو کچھ مل جاتی ہے وہ ہو جائے۔ اس سے کافی ہیلپ (Help) ملتی ہے۔

N: ٹھیک ہے۔ تو آپ کے مطلب سے سیمینارز (Seminars) وغیرہ ہونے چاہئیں اور یہ چیزیں ہوں گا جائیں تو اس سے چیزیں امپروو (Improve) ہو سکتی ہیں۔ اس کے علاوہ کیا اپنی رائے یا سجیشن (Suggestion) دینا چاہیں گی۔

R: مطلب کس حوالے سے؟

N مطلب نیوٹریشن کیئر (Nutrition & care) کے حوالے سے جیسے آپ کی کوئی ایڈیشنل (Thought) تھوٹ ہے یا کوئی ڈاکر ہے یا کوئی بھی چیز۔

I

R ایک تو یہ سوشل میڈیا کا بھی دور ہے تو مین سورس آف نولیج (Main source of knowledge) Instagram اور facebook بھی ہے تو اس پہ تو لیٹ (awareness) اویرنس اور (knowledge) آبویسلی (obviously) جو کہ چاہیے وہ یہ کام سے بہتر Nutritionist (awareness) کر سکتے ہیں تو وہ اویئرنیس کو بہتر کر سکتے ہیں۔

N تو اس سے جو (Masses) ماسس کو محبت صحیح (awareness) اویئرنیس کر سکتے ہیں۔ ٹھیک ہے ایسا ہی ہو Dr. آپ کا بہت شکریہ (Thank you)

R تھینک یو
