# Supplementary material for: Qualitative exploration of comprehension and experiences of healthcare professionals regarding nutrition care in Karachi, Pakistan
Source: PLOS Glob Public Health. 2025 Dec 30;5(12):e0005483. doi: 10.1371/journal.pgph.0005483 (PMC12753000; doi:10.1371/journal.pgph.0005483)
Supplement: S5 File — (ZIP) [file pgph.0005483.s005.zip › Doctor Female -003.pdf]

Date [redacted] - DF-3 Doctor Female-003

اسلام علیکم I

وعلیکم اسلام M

میرا نام نیرہ طاہرہ سید ہے میرا تعلق [illegible] سے ہے ہم لوگ جو ہیں qualitative Research کر رہے ہیں جس کو [illegible] different health professionals کی رائے اور ان کی خیالات اور [illegible] جاننا چاہ رہے ہیں [illegible] care Regarding تو یہ کہ ہم آپ سے جو ہیں اس سلسلے میں کچھ سوالات کریں آپ کو اختیار ہے کہ آپ جس سوال کا جواب دینا چاہیں اور جس کا نہیں اور کسی جس سوال کا جواب نہیں دینا چاہتی ہیں تو آپ کا اس [illegible] پر کوئی اثر نہیں پڑے گا آپ کبھی بھی اس Interview کو ختم کر سکتی ہیں اور آپ جو بھی معلومات اس [illegible] میں دیں گی وہ صرف Research Paper کیلئے use ہوں گی جب ہم اس کی Analysis وغیرہ کریں گی تو آپ کا نام اور کوئی بھی [illegible] کو Remove کر دیا جائے گا جو بھی معلومات ہمیں ملیں گی ہم اس کو [illegible] Publication کیلئے بھی use کریں گے مگر کوئی بھی time پر آپ کی کوئی Publication [illegible] share نہیں کی جائے گی جو ہے وہ Important information completely Anonymous رکھا جائے گا [illegible] اگر آپ اس [illegible] کو Agree کرتی ہیں تو آپ اپنا [illegible] ہمیں دیں Please verbally consent دیں تو [illegible] اس چیز کی اجازت دیتی ہیں I

جی جی اجازت ہے M

جی اچھا ٹھیک ہے سب سے پہلے تو آپ اپنے بارے میں بتائیں کہ آپ کا [illegible] جو بھی آپ کیا کر رہی ہیں I

میرا نام [redacted] ہے میں یہاں پر RMO کی Job کر رہی ہوں M

Date

[redacted] میں MBBS ڈاکٹر ہوں
اس میں MBBS کے بعد میں نے House job
میں نے JPMC سے کیا ہے
اپنے بارے میں بتا رہی تھی کہ میں
SMC سے MBBS کیا ہے پھر ایک سال House
job JPMC سے کیا ہے باقی ابھی میں یہاں
پر RMO کی Job کر رہی ہوں Hashmani
کے Hospital میں
ٹھیک ہے آپ جب سے یہاں پر کام کر رہی ہیں تو
آپ کی کیا Job Schedule ہے؟
میری Job responsibilities میں یہ ہے کہ M
میں general ward ہوتا ہے
اور Private ward میں جو Patient
آتے ہیں اس کی History لینی ہوتی ہے
treatment وغیرہ لکھنا ہوتا ہے وہ ہمیں دیکھنا
ہوتا ہے جو بھی consultant کے order
ہوتے ہیں ہمیں ان کو follow کرنا ہوتا ہے
اور Patients کو ظاہر ہے ہمیں monitor
کرنا ہوتا ہے کہ کوئی بھی اگر active complaint
ہو تو اس کو ہم دیکھ لیں consultant کو
بتا دیں اور کوئی بھی treatment ہو
on the spot وہ دے دیں
تو آپ لوگوں کا work load کافی ہے I
ہمارا work load ہے تو کافی ہے کبھی M
کبھی ایسا بھی ہوتا ہے کہ ICU میں ہے
کوئی Doctor available نہیں ہے تو ہم
ICU میں بھی چلے جاتے ہیں ہم ER میں
کوئی available نہیں ہوتا تو ER میں
بھی ہماری کبھی کبھی duty لگ جاتی ہے
ٹھیک ہے ٹھیک ہے اچھا آپ کا Nutrition care I
کے حوالے سے کیا خیال ہے جو آپ کر رہی
ہیں اس کے دوران آپ کو موقع ملتا ہے
یا آپ سے لوگ سوالات کرتے ہیں کہ

Date

پیشنٹ counseling کے بارے میں nutrition کے بارے
میں بتا کہ ہمیں یہ چیز کھانی ہے یا نہیں کھانی
ہے یا کیا کرنی ہیں

(بالکل nutrition تو ایک بہت important 18
کا حصہ ہے ہماری زندگی کا) all of Fluid
تو یہ کہ بلکہ patient اگر یہاں پر adult
ہوتے ہیں تو ان کو یہ concern ہوتا ہے کہ
بھی بیماری جو جیسے surgery کا اگر کوئی
patient ہے GS کا کوئی بھی ہے تو
post op بہت concern ہوتے ہیں کہ
بھی nutrition بیماری کیا ہوگی diet
فوراً سے تو ابھی liquid یا Hard diet solid diet
نہیں دے سکتے تو ہم پھر ہم ان کو آہستہ آہستہ
ہم چیزیں بتاتے ہیں کہ آپ کو کہاں سے
شروع کرنا ہے liquid سے soft diet start کریں
گے اور پھر آہستہ آہستہ solids کی طرف
جائیں گے اور پھر کیا بر ہے soft کے
علاوہ healthy diet کو بھی ہونا چاہیے
تو ہم یہ بھی suggest کرتے ہیں کہ
patients جو بھی healthy لیں کوئی
بھی ایسی چیز نہ لیں جو کہ unhealthy ہو
جو ان کی health کو effect کرتے
یعنی کہ آپ ان کو counsel کر رہی ہوتی I
ہیں بتا رہی ہوتی ہیں

بالکل کرتے ہیں (یہاں ہم ہماری nutritionist 19
بھی ہیں الگ سے available ہوتی
ہیں باقی ہم بھی patients کو counsel
کرتے ہیں۔ تو دو تین دفعہ patient
counsel ہو جاتا ہے) اب ظاہر ہے کوئی بھی
انسان کا بھی ہے کہ ایک چیز اگر اسکو دو تین
بار بتائی جائے تو پھر وہ سمجھتا ہے کہ بھئی یہ
important چیز ہے اس کا خیال رکھنا ہے
تو وہ رکھتا ہے ظاہر ہے پھر

CamScanner

Date

T: ہمم لو ایک خیال سے آپکو کیا مشکلات
پیش آتی ہیں counseling کرنے کے مطلب
nutrition کے بارے میں بتانے کیلئے۔
M: کچھ Patients ہوتے ہیں تو reluctant
ہوتے ہیں جن کو تو ہے وہ اتنا خیال نہیں
ہوتا ہے اپنی صحت کا یا خیال اگر ہوتا بھی
ہو تو ان کو لیس سا کہ سمجھ جانے ذہن اس
چیز کو اتنی کوئی خاص important نہیں ہے
تو وہ کچھ patients کا ہوتا ہے یہ مسئلہ
لیکن زیادہ تر Patients جو ہیں مان جاتے
بات وہ کوئی اتنا مسئلہ نہیں ہوتا ان کو
دو تین بار ان کو اگر ایک ہی بات سمجھائی
جائے طریقہ ہوتا ہے counseling کرنے کا صحیح طریقہ
سے اگر صحیح کی جائے counseling تو وہ سمجھ جاتے
ہیں بات کو
T: ہمم تو آپکا کبھی اتفاق ہوا کہ آپ جو
ہیں community میں group کے اندر جو
تھے وہ اپنے counseling وغیرہ کرتے ہیں یا
nutrition یا medication کے حوالے سے کوئی بات بتا
سکیں لوگوں کو۔
آگے سے تو میرا ایسا کوئی Experience
نہیں ہوا کہ الگ سے میں Patient counsel کروں
کرتی لیکن اس سے پہلے میں جہاں پہ کام کرتی
تھی health SINA کرتا ہے وہاں پہ
یہ تھا کہ وہاں پہ counseling پہ زیادہ focus
تھا as compare to providing medicine
وہاں پہ medicine پہ اتنا focus
نہیں، کہتے تھے Patient کو ڈاکٹر تو
بھی تھے تو ہم ان کو educate زیادہ کرتے
تھے ان کی condition کے بارے میں اور
ان سے یہی کہتے تھے کہ بھئی medicines آپ
کم سے کم کوشش کریں کہ کم سے کم استعمال میں
آئیں اور آپ اپنی diet سے

Date

ہے چیزیں وہ چیک کریں تو وہاں پہ بہت زیادہ
مسئلہ یہ تھا کہ میں Patients نے diet
کو بتاتے اگر ان for example اگر کوئی
LTP Patient
ہے جو constipation کا تو ان کو یہ ہے کہ ہمیں بھی
laxative چاہیے دیں ہمیں کوئی دوا دے دیں
جس سے ہم فوراً ٹھیک ہو جائیں تو ہم کوشش
کرتے تھے کہ آپ diet سے ٹھیک کرنے کی
کوشش کریں اس سے اگر ٹھیک نہیں
ہوتا تو پھر آپ medicine کی طرف آئیں
تو اس میں صرف ہم ان کو بتاتی تھیں کہ بھئی
کیا کیا آپ diet لیتی ہیں آپ
جو ہے [illegible] لے سکتی ہیں تو اس سے
ٹھیک ہو جاتی ہے constipation آپ fluids
کا استعمال زیادہ کر سکتی ہیں آپ اپنے
diet میں fiber ایسے سبزیاں ہیں تو آپ کا
constipation relief ہو جائے گا تو یہ
ساری چیزیں ہم وہاں کرتے تھے تو اس
میں کافی بہتر nutrition experience ہے
کے حوالے سے زیادہ

آ: تو وہاں پر آپ کو کوئی مشکلات پیش آتی
تھیں کہ مطلب عمل نہ کر رہے ہوں

M: وہاں پر سب سے بڑا مسئلہ جو تھا وہ تھا کہ
patients جنس [illegible] علاقے سے تھے وہ
under privileged area تھا تو وہاں پر جتنے بھی
patients آتے تھے وہ ایسے ہی تو سب
سے بڑی بات تو language barrier بہت تھا
وہاں کا دوسرا یہ کہ patients جو ہے وہ
سمجھتے نہیں تھے بات کو ظاہر ہے ان کو اتنا
شعور نہیں تھا وہ بس ان کو سمجھاتے تھے
ہم کوشش ہم اپنی طرف سے پوری کرتے تھے بعد
میں سمجھانے کی لیکن ان کا یہی problem
ہوتا تھا کہ وہ مسئلہ کرتے تھے کہ ہمیں دوائی
دیں دے دو اور میں ہم مطلب وہ instant relief

Date ______

چاہئے تھی | پہلے اس کا مطلب یہ ہم سے جتنی کوشش
ہو سکتی تھی ہم کر رہے تھے educate کر لیں Patient کو
کرنا کہ وہ اس چیز کو سمجھیں کہ یہ تھی Medicines
پر اتنا بس focus نہیں ہونا چاہیئے [illegible] پہ
ہمیں پہلے بس Focus رکھنا ہے

I اسکا مطلب ہے وہاں پر تو کافی کام ہوا ہوگا ایریا
M وہاں پر تو بہت exposure ملا تھا also area
ایسا تھا تو وہاں پر Patients بھی بہت آتے
تھے تو میرا تو کافی زیادہ experience رہا ہے
وہاں پہ 60 Patients میں دیکھتی تھی
OPD per day میں
I اچھا
M تو 60 Patients اگر آ رہے ہیں تو اسی طریقے
کم آ رہے ہیں سب سب کا یہی تھا
I اچھا تو work load کافی ہوتا تھا ادھر تو
M workload تو بہت زیادہ ہوتا ہے Patients
ایک دن میں کیسے دیکھتی ہو
I اور پھر ان کو سب چیزیں بتانا explain کرنا Prescribe
M یہ face کرنا language barrier دیکھنا سب
سے پہلے تو آپ کی بات ان کو سمجھ
ہی نہیں آ رہی Translator منگوانا
Translator بھی busy ہوتے تھے وہ
آئے پھر ان سے بات کر لیں پھر اپنی
بات Convey کرنا بہت مشکل ہوتا تھا
لیکن some how ہو ہی جاتا تھا
I تو وہاں پھر ان کو ان کے اندر کیا
مسائل لگتے تھے زیادہ تر مریضوں کے اندر
M وہاں پر Patients کا مسئلہ یہ تھا ایسے
زیادہ تر آتے تھے Pediatric Population زیادہ
آتی تھی کیونکہ وہ Family medicine کا clinic تھا
تو وہاں پر Pediatric Patients کا ظاہر ہے
وہی upper respiratory tract infection
low infection باقی hygiene کا بڑا مسئلہ تھا

Date ______

تو hygiene کے حوالے سے ان کو Patient
زیادہ تر Scabies کے ہمیں مل رہے
ہوتے تھے۔ کوئی بھی skin infection ہوئے
ہوتے تھے اب اس میں ڈاکٹر سے ہم counsel
بھی کرتے تھے کہ بھئی hygiene کا بھی خیال
رکھیں medicine بھی properly دیں
ہم جو جو local application دیتے ہیں
وہ بھی ٹھیک سے لگائیں لیکن یہی تھا
nutrition کے حوالے سے غذا کے حوالے T
سے ان کو کیا کہتے تھے؟
غذا کے حوالے سے بھی ہم یہی بتاتے تھے کہ M
کوئی بھی کوئی بھی مرض یا condition
ہو تو اس کے حوالے سے diet plan میں
فوراً بتاتی Patient کو اب جیسے For
instance کوئی بچہ آیا ہے اس کا
ان کی mothers نے upper respiratory tract infection
میں ہے تو Antibiotics مل جائے [illegible]
مل جائے لیکن میں ان کو یہی کہتی تھی
کہ بھئی دوا سے پہلے آپ کوشش یہی کریں
اگر ایسا Bacterial کی طرف نہیں گیا مسئلہ
ابھی تک viral ہی ہے تو آپ steam لے سکتے ہو
آپ Hot fluids لے سکتے ہیں soup
یخنی یہ ساری چیزوں سے ٹھیک ہو جاتا ہے معاملہ
Antibiotics تک بات نہیں جاتی تو یہ ساری
چیزیں ظاہر ہے ہم اکثر سمجھاتے ہیں۔
تو ڈاکٹر مطلب اس نسخے پہ عمل کرتے تھے T
وہ لوگ یعنی آپ کو بتا رہی ہوں M
نا عمل کرنے والے کرتے تھے لیکن جو
جن کو ہوتا تھا کہ بھئی فوراً ٹھیک ہو جائے بچہ
وہ تو ظاہر ہے ان کو مشکل ہوتی تھی ان
کو بات سمجھنے میں
تو آپ کیا suggest کریں گی کہ مطلب T
ہم کس طرح improve کر سکتے ہیں مطلب یہ کہ

Date ____________

redundance جو ہے Improve سو

Improve تو دیکھیں اسی طرح کر سکتے ہیں M
کہ (لوگوں میں sentence ایسے ہی آ رہے تھی
جب آپ ایک بات اُن کو بار بار بتائیں گیں
بار بار بتائیں جیسے یہاں پر ہو رہا ہے کہ
redundant ایسے ہیں ہم الگ بتا رہے ہیں
patients کو تو مطلب ایک بات جو ہم بتا رہے
ہیں اور وہ چیز اُن کو دو تین جگہوں سے
اور سننے کو مل رہی ہے تو اُن کو لگے گا کہ یہی
یہ چیز important ہے اسکا خیال رکھنا ہے)

یعنی کہ چیز بہت same ساری جگہوں سے I
اگر بار بار سننے کو ملے گی

بار بار اگر سننے کو ملے تو ہم اس میں ظاہر M
ہے فائدہ ہی فائدہ ہوگا

تو ہم confusion کہاں آ جاتی ہے patients I
کے اندر کہاں وہ confuse ہو جاتے ہیں زیادہ
کہ ہم یہ کریں یہ نہ کریں

(confusion تو As such نہیں ہوتی M
patients میں میں نے تو یہی دیکھا ہے
کہ وہ بس thats it ہوتے ہیں کہ
ہمیں بتایا ہے کہ یہ چیز ٹھیک ہے ہمارے لیے
یہ نہیں کرنا مطلب ہم نہیں کرنا چاہ
رہے) جیسے میں نے آپ کو SINA Health
کا بتایا وہاں پر patients کا یہ تھا کہ
بھی اپنا ہی diet follow کر جاتے diet
وہاں چھوڑ دو medicines یہ چیز
تو تھی

یعنی کہ آپ کے کہنے کا مقصد ہے اُن I
patients کو کوئی اُس چیز کی importance
کا پتا ہی نہیں ہے

importance کی پتا نہیں ہوتی health M
ہاں بھی میں کہہ رہی تھی importance کا نہیں
پتا ہوتی اب ایک ہی چیز بار بار لوگ

Date

ہیں گے کوئی doctor کہہ رہا ہے کوئی
nutritionist کہہ رہا ہے تو ہم ان پہ ظاہر ہے
ان کو important کا پتا چلے گا کہ بھئی یہ
چیز ہے اسکا خیال رکھنا ہے
Hmm ٹھیک ٹھیک تو آنٹی کہا کہ اپنے I
community کے حوالے سے اپنے بتایا کہ
community کے اندر بھی اتنا اتنا نہیں
ہوا تو family کی اپنی community
وغیرہ family community ہوتی ہے اس
کے اندر بھی کبھی رائے وغیرہ آپ
سے مانگی گئی ہو کہ اچھا یہ
کیا کھانا ہے یا یہ کس طرح کرنی ہیں mom
family میں تو کوئی [illegible] M
ابھی تک تو میرے ساتھ کچھ نہیں ہوا کہ NICU
کسی نے مانگا ہو
ٹھیک ٹھیک اور کوئی ایسی کوئی I
suggestion دینا چاہیں گی کو [illegible]
thoughts ہمارے ساتھ share کرنا
چاہیں گے
نہیں جی بس یہی میں نے جواب کو بتا لیں M
جتنے بھی بس یہی ہیں that's it
improvement کیلئے ہم اور کیا کیا I
چیز کر سکتے ہیں
improvement کے لئے بس یہی ہے کہ M
awareness بڑھانے کیلئے آپ لوگ
جیسے nutritionist رکھی ہوئی ہیں
Hospital میں nutritionist کا ہونا بہت
ضروری ہے الگ سے
ٹھیک ٹھیک I
تاکہ وہ education کریں Patients M
الگ سے کر
Hmm education جو ہے very important I
ہے

Date ______

M: بہت Important ہے awareness پھیلانا لیکن
بچوں میں کم diet اپنا [illegible]
Important aspect ہے

I: Haan ٹھیک ہے ٹھیک
Thank you so much

M: You are welcome
