# Supplementary material for: Qualitative exploration of comprehension and experiences of healthcare professionals regarding nutrition care in Karachi, Pakistan
Source: PLOS Glob Public Health. 2025 Dec 30;5(12):e0005483. doi: 10.1371/journal.pgph.0005483 (PMC12753000; doi:10.1371/journal.pgph.0005483)
Supplement: S5 File — (ZIP) [file pgph.0005483.s005.zip › Doctor Female -004.pdf]

Date [redacted] Doctor Female-004

ہم PhD students ہیں qualitative Research کر رہے ہیں I
تو اس کے اندر ہم کچھ سوالات کریں گے
اور آپ کی مرضی ہے آپ جس کا جواب
دینا چاہیں یا نہیں اور یہ ساری آپکی معلومات
Research Purpose کیلئے use ہوں گی اور اس
کے علاوہ جب ہم Publish کریں گے
تو آپکا نام anonymous رہے گا تو
اگر آپ Agree کرتی ہیں تو اپنا
consent دیں Thanks

جی J

اچھا تو سب سے پہلے تو آپ Thank you I
اپنے بارے میں بتائیں

[redacted] نام ہے میرا gastro کی J
PG

Okay تو آپ کو کتنا عرصہ ہو گیا ہے I
یہاں پر کام کرتے ہوئے اور کیسا Patient flow رہتا ہے

Average ہے J

تو آپ کیا سمجھتی ہیں کہ nutrition care I
کے بارے میں جب ہم بات کرتے ہیں اس حوالے
سے تو آپ کے mind میں کیا چیز آتی ہے
آپ کیا سمجھتی ہیں کیا ہے یہ

غذائی صحت، اچھی healthy diet J

آپکا Patient سے Interaction ہوتا ہے I
آپ سے nutrition care کے بارے میں پوچھتے ہیں

نہیں ہم سے gastro سے related J
stomach problems پوچھے جاتے ہیں کہ question
ہے تو ہم کیا چیزیں نہ کھائیں تو اس
سے related ہم یہ advice کر دیتے ہیں
کہ یہ چیزیں نہیں کھانی۔ یہ چیزیں کھانی ہیں

اچھا تو آپ کے خیال میں جو healthy diet I
ہے وہ Patient کی nutrition care ہے اور جو
میں جیسے gastro سے ایک ہے تو آپ

Date ______

Patients کو nutrition کے حوالے سے بیماری کی
ہوتی ہیں کیا کھانا ہے اور کیا نہیں کھانا
تو یہ بیماری ہوتی ہیں آپ ان کو لیکن کبھی
ایسا ہوتا ہے کہ آپ کو مشکلات پیش آتی
ہیں کہ بات اتنی اچھی نہیں کر پا رہے اپنی
بہت کا خیال

J ہاں sometimes ایسا ہوتا ہے کہ بات کچھ
Patients ہوتے ہیں نا ان کو to the point
بہت لمبی [illegible] چاہیے ہوتی ہے اگر آپ
کے پاس ایک pamphlet یا sometimes
کیا ہوتا ہے ایک ہر چیز images پہ یاد ہوتی
ہیں یہ کرتی ہے یہ follow نہیں کرتی Advice
(کہ nutrition کا کیا ہوتا ہے sometimes
چیزیں یاد رہتی ہیں کچھ چیزیں یاد نہیں ہوتیں
جیسے high protein کس میں زیادہ ہے کس میں
potassium کس میں زیادہ ہے اس طرح کی چیزیں
یاد نہیں ہوتیں)

T تو اس کیلئے کیا ہونا چاہیے

J (چھوٹا سا کوئی pamphlet یا ایسی کوئی
چیز ہونی چاہیے جیسے اس میں ہمارے
پاس basic چیزیں ہوں تاکہ high protein
food ہے کیسی patient کو اگر ہمیں سمجھانا
ہے تو ہمارے پاس کچھ ایسی چیزیں ہوں جو ہم
patient کو hand over کر دیں) کہ یہ
آپ کی diet ہے اس میں اتنا protein ہے یہ
Junk food ہے یہ avoid کرنی ہے
(کیونکہ ہر patient کا ایک جیسا IQ
نہیں ہوتا جو highly educated یا literate ہوتے
ہیں وہ سمجھ جاتے ہیں جو layman ہوتے
ہیں وہ ایک ایک چیز پوچھ رہے ہوتے
ہیں کہ یہ کھانی ہے یا یہ نہ کھائیں) یہ
چیزیں کریں وہ نہ کریں اس کیلئے ہم [illegible]
[illegible] جاتیں اس کیلئے ہمارے پاس ایسے

کوئی چیز ہو (ایسا کوئی problem ہو یا امداد [illegible]
[illegible] جیسے ہم بہت سارے Patients کو
[illegible] کے پاس بھیج دیتے ہیں) جب ہمیں
لگتا ہے کہ [illegible] Patient بہت زیادہ question
کر رہا ہے) تو ہم انکے پاس بھیج دیتے ہیں تاکہ
وہ handle کر لیں

یعنی کہ کوئی ایسی چیز ہو یا [illegible] ہو Hmm — I
یا [illegible] کوئی

ہاں (کوئی ایسی چیز ہوتی ہے جو ہم Patient — R
کو hand over کرتے ہیں اور وہ انکے لیے بھی
Easy ہو جائے گا اور ہمارے لیے بھی Easy
ہو جائے گا

لوگ کیا کبھی اتنا ہوا [illegible] Experience ہوا — I
کہ آپ [illegible] میں جا کر یا community Public میں
[illegible] میں اس طرح کی Counseling کرتے
ہیں ان کو بتاتے ہیں غذائی صحت کے حوالے
سے کچھ

نہیں Public [illegible] — R

Public means [illegible] people — I

ہاں وہ کیا ہے — R

یہ کس طرح سے وہ Patients ہوتے ہیں — I

Patient ہوتے ہیں ان کے attendants ہوتے — R
ہیں ان کے family members یا Sometimes
ایسا ہوتا ہے کہ family gathering ہوتی
ہے تو بہت سارے لوگ اکھٹے ہوتے ہیں
ہیں more or less same questions کے
پوچھ رہے ہوتے ہیں Same question
کے answers ہوتے ہیں

Family میں یعنی خاندان — I

Socially کہیں پہ بھی لوگ آتے ہیں — R
اور آپ سے بات کرنے لگ جاتے ہیں
کہ gastro کیلئے

gastro کو بہت سی [illegible] ہے تو — I

Date ________

اُس میں basically کس کس پر focus کر رہے ہوتے
ہیں اور آپ اس کو کیا بتا رہے ہوتے ہیں
آج کل تو ظاہر ہے جو زیادہ آ رہے common problem I
ہوتے ہیں junk food وغیرہ زیادہ لیتے ہیں
تو gastric upset کے ساتھ patient آتے
ہیں اور اُن کا best solution یہی diet
ہوتا ہے کہ اپنے diet کو properly follow کریں اپنی
اور اپنا stomach ٹھیک ہو جائے گا
ٹھیک تو اگر ہم level پر patient کی بات I
کریں یا غذائیت کی بات کریں جس
طرح لوگ ہیں دال چاول یا کھانا کھا رہے ہوتے
تو آپ اُن کو کیا مشورہ دینا چاہیں گے یا
کیا آپ کا opinion ہوگا کہ کس
طریقے سے وہ اپنی health یعنی غذائی صحت
جو ہے وہ اچھی کر سکتے ہیں
I think (ایک تو ہمارے پاس ایک I
awareness نہیں ہے ہم لوگ junk food اور
باقی جو چیزوں کی طرف بہت زیادہ چلے
گئے ہیں وہ جو nutrition ہوا کرتی healthy habits ہوا کرتی
ہیں نا وہ some how بہت دور رہ گئی
ہیں even ہم Doctors خود بھی اُن
چیزوں کو follow نہیں کرتے ہم لوگ
سارا دن یہاں پر wards میں ہوتے
ہیں تو ہم بھی healthy diet نہیں لیتے جس کی
وجہ سے سارے problems ہو رہے ہوتے
ہیں تو یہ والی چیز تو ہے So I would suggest
یہ جو (ہمارے بیچ میں ایک gap ہے کہ لوگوں
کا جو lifestyle ہے وہ نہیں ٹھیک اس سے
وجہ سے imbalance nutrition ہوتا ہے اور آپ
کی ساری problems شروع ہوتی ہیں
اُس لیے ہم کیا کر سکتے ہیں I
I think تھوڑی بہت awareness I
I would suggest ہر patient level

ایسا ہونا چاہیے اگر ایک OPD یا OPD
کے باہر بیٹھے بیمارا تو بہت لمبا ہوتا ہے انتظار
ہے اُس چیز سے لمبا بیٹھے لوگ انتظار تو
کوئی ایسا ایک بندہ بیٹھا ہو یا کوئی ایسا
ہو Assistance staff کہ وہ جو اُن لوگ
جن کو نہیں سمجھ میں آتا ہے اُن کے پاس
جا کر ان سے Enquiry کریں تاکہ ہمیں پتہ چلے
کہ اگر ان کو Patient لئے کو Problem ہوتا ہے
تو وہاں سے بھی ایک visit ہو جائے

I: آپ کے خیال میں Nutrition care جو ہے وہ
سب کو کون Provide کر سکتا ہے

R: Nutritionist

I: تو وہ جو Attendance ہوتے ہیں جیسے آپ [illegible]
نے بات کی کہ ہونا چاہیے تو اسکی qualification
کیا ہونی چاہیے

R: نہیں وہ Educated ہونا ہے ظاہر ہے وہ بالکل
پڑھ کر نہیں سمجھائے گا ظاہر ہے Consultation
جتنا میں نے سنا ہے کہ اُس میں basic
ہوتے ہیں ایک Nutrition اور ساری چیزوں
کا پتہ ہو تو ظاہر ہے Consultation کر سکتا ہے

I: Pakistan کی اگر ہم Nutrition care کی
Public Hospital سے ہٹ کر over all
کی بات کریں تو آپ کے خیال میں کوئی ایسی
چیزیں ہے Challenges ہیں اور اُن کا کوئی Solution
ہو سکتا ہے ایسے کوئی مسئلے ہیں پاکستان کے
Overall اگر ہم Population کی بات کریں
اور اگر مسئلے ہیں تو انہیں کیسے Solve
کیا جائے۔

R: مسئلے ہمارے Myths بہت ہیں ہم چیزیں
کھاتے ہیں گرم ہوتا ہے یہ ٹھنڈا ہوتا ہے یہ
والی چیز mind set سے ہے یہ Problem ہے
ہمارا یہ نہیں کھاؤ اُس سے یہ ہو جائے گا یہ
چیز کھاؤ گے تو یہ ہو جائے گا۔ اسکی

Date ______

وجہ سے کافی چیزیں Problem ہے اس کا بس
یہ ہے کہ تھوڑا سا آپ نے counseling کرنی ہے
Myth وغیرہ بہت ہیں تو اس کیلئے them I
counseling کر سکیں

پہلے counseling کریں awareness اتنی ہونی چاہئے J
کہ وہ اُن کو ایسے ٹھنڈا گرم یہ لوگوں
کے دماغ میں ہوتا ہے یہ نہ کرو وہ نہ کرو
ویسا نہ کرو ویسا نہ کرو ایسا کرو یہ کھاؤ گے
تو یہ ہو جائے گا جیسے simply اگر کہوں تو
کسی کو chest infection ہے تو اس کو دہی نہیں
کھانے دی جاتی ہے ایسا اس کو چاول نہیں کھانے
دیے جاتے ہیں اس کا such as کوئی relation
نہیں ہے آپ پڑھ لیں کہیں یہ نہیں لکھا ہوا کہ
یہ Patient کو کوئی اگر chest infection ہے اور
وہ چاول کھائے گا تو خراب ہو جائے گا مطلب
یہ Myth ہے۔ دہی نہیں کھا سکتا گلا خراب
ہو جائے گا

تو اس کیلئے best platform کونسا them I
ہونا چاہئے

I think social media best platform J
platform ایسا ہے I would suggest social media
ہمارے پاس آ گیا ہے جو بہت پاورفل ہے
آپ لوگوں کو communicate کر سکتے ہیں
اور لوگ پہلے easily لے بھی لیتے ہیں
بھی اگر ہم کوئی seminar کرتے ہیں تو
Public آپ کے پاس جو totally
ہے وہ بہت کم آتی ہے social media پہ جیسے
چھوٹے چھوٹے messages اگر nutrition کے حوالے سے
ڈال دیے جائیں social media پہ کہ کوئی بھی
potassium related سے carbohydrates ایک
multivitamin زیادہ بہت related سے
کس میں ہیں کون کون سے fruit کھانے
سے یہ vitamins & minerals ہمیں مل سکتے

Date ______

تو اسی چھوٹی چھوٹی videos ہم بنا کے social
media پہ share کر لیں تو یہ ہر ایک لوگوں کا
awareness چھوٹی چھوٹی بن جاتا ہے videos
تو ان میں awareness بڑھ جائے گی
ہم seminars میں ہوتا ہے کہ بہت کم لوگ
آتے ہیں properly اپنا کام چھوڑ کر آنا
پھر وہ attend کرنا جو بہت زیادہ willingness
ہوگا وہ جائے گا otherwise نہیں جائے گا
صبح اور شام social media کو basically
وہ لوگ دیکھ رہے ہوتے ہیں
اس level کے لوگ دیکھ رہے ہوتے ہیں
چھوٹے سے چھوٹا اور بڑے سے بڑا اور اگر
سادہ میں بات کی جائے layman language میں
سمجھایا جائے تو ہر کسی کو سمجھ آ جائے گی
اور اگر ایسا کوئی opinion ہو تو آپ
کچھ add کرنا چاہیں کچھ بھی
یہی چیزیں کہ کچھ ایسا ہونا چاہئے
nutrition کا کیونکہ ہمارا problem سارا
جو issue آ رہا ہے نا یہیں سے آ رہا ہے
جیسے ہم gastro کی field میں ہیں
ہمارے پاس 70% problems میں تو diet
سے ہیں، Hepatitis ہو رہا ہے vomiting
ہو رہی ہے gastric upset ہو رہا ہے Abscess
ہو رہا ہے enteritis ہو رہا ہے diet
ٹھیک نہیں ہے nutrition [illegible] پانی سے
آپ کو چیز جو basic وہ کم آپ
صحیح کر دیں گے تو باقی چیزیں automatically
بھی ٹھیک ہو جائیں گے۔ ٹھیک ہے

Thank you so much
