# Supplementary material for: Qualitative exploration of comprehension and experiences of healthcare professionals regarding nutrition care in Karachi, Pakistan
Source: PLOS Glob Public Health. 2025 Dec 30;5(12):e0005483. doi: 10.1371/journal.pgph.0005483 (PMC12753000; doi:10.1371/journal.pgph.0005483)
Supplement: S5 File — (ZIP) [file pgph.0005483.s005.zip › Doctor Female -005.pdf]

Date [redacted]

Doctor Female-005

م اسلام وعلیکم

آ وعلیکم السلام [redacted] بات کر رہی ہیں

م جی بالکل

آ میں [illegible] بات کر رہی ہوں میں [illegible]
[illegible] university میں lecturer ہوں اس [illegible]
سے actually یہاں پر ہوں PhD Scholar [illegible] ہماری
ایک qualitative analysis ہے Research ہے تو اس
میں ہم explore کر رہے ہیں qualitatively Health
Perceptions & practices [illegible] care providers
کے حوالے سے تو میں آپ سے کچھ سوالات
پوچھوں گی اگر آپ اجازت دیں تو اگر آپ
کو کسی سوال کا جواب دینے میں آپ نہ دینا
چاہ رہی ہوں تو آپ بیشک اس کا جواب
نہ دیجیئے گا اس پہ ہمارے [illegible] پہ کوئی
فرق نہیں پڑے گا اس پر

م ٹھیک ہے

آ اور دوسری بات جو آپ سے یہ کرنی تھی آپ
کی یہ جو ہے وہ جتنی بھی ہماری گفتگو ہو گی
یہ Research Purpose کیلئے استعمال ہم Information
کریں گیں اور اس میں آپ کا نام بالکل بھی نہیں
آئے گا results [illegible] بھی جب ہم کام کر رہے
ہوں گیں یا [illegible] تو آپکو anonymous رکھا
جائے گا آپ جو بھی کہیں گی جو بھی آپ سے سوالات
ہوں گیں جو بھی جوابات ہوں گیں اس کو بالکل anonymous
اس [illegible] کیسے بنائیں گیں اگر آپ مجھے اجازت
دیتی ہیں آپ کا Interview لینے کی تو آپ Consent
دیتی ہیں تو Please verbally آپ مجھے اجازت
دے دیں تاکہ ہم آگے Procedure کریں

م جی ٹھیک ہے کریں

آ آپ مجھے Please یہ بتائیے اپنے بارے میں تھوڑا
سا بتائیں آپ کہاں پر Job کر رہی ہیں کتنے
عرصے سے ہیں کتنا [illegible] کا [illegible] سے

M [redacted] تقریباً ۹ سال ہو گئے ہیں

Date ____________

کراچی میں صفورا پہ ہے وہاں پر کام کرتی ہوں
ہے Private [illegible] hospital

ٹھیک ہے تقریباً Patients کا average I
کتنا ہوتا ہے آپ کے پاس ایک OPD
میں

Every OPD کا دیکھا جائے تو تقریباً 20، 25 M
کبھی کم بھی ہوتا ہے کبھی 40s بھی ہو جاتے
ہیں اور depends ہے

ٹھیک ہو گیا اچھا آپ مجھے یہ بتائیے I
Patients کو nutrition کے حوالے سے لوگوں سے
بات چیت کرنے کا آپ کو یا counselling کرنے کا
بھی موقع ملتا ہے

جی بالکل M

اچھا تھوڑی سی اسکی تفصیلات بتائیں گی I
کس حوالے سے کیا پوچھتے ہیں آپ انکو کیا
جواب دیتی ہیں

Specially Acne related جو Patients آتے ہیں M
تو انکا nutrition care جو مجھے پتا چلتا ہے انکی
meals دیکھ کر کے that's not good اور زیادہ تر
Patients یہاں کراچی کے ہوتے ہیں جو ہم بہت
زیادہ Junk food کرنے والے ہوتے ہیں
دوسرے لحاظ سے اگر دیکھا جائے تو کچھ اور
بیماریاں بھی ایسی ہوتی ہیں Psoriasis
ہے جس کے اندر بڑے کا گوشت کھانے سے
بیماری بڑھ جاتی ہے اور بھی کچھ ایسی diseases
ہوتی ہیں جن کے اندر Allergies ہوتی
ہیں جن میں milk، meat، fish، Egg
سے Allergies کی Patients بڑھ جاتی
ہیں

ٹھیک ہو گیا جی جی آپ کچھ کہہ رہی تھیں I

جی پوچھیں اور M

اچھا تو مجھے یہ پوچھنا تھا آپ سے I
کہ جیسے آپ کا تو background medicine میں

Date ________

ہے اور Counseling کی جب بات آتی ہے تو وہ
آپ خود کر رہی ہوتی ہیں

نہیں جتنا میرے بس میں ہوتا ہے Patient M
کو تھوڑا بہت counsel کر دیتی ہوں کہ بھئی
Junk نہیں کھانا یہ healthy eat کریں
صبح یا شام کی 30 min walk, 2 glasses of milk
سو ہر [illegible] کے اندر amount of [illegible]
fruit intake, 2 serving vegetables, vegetables 2
Patient کو [illegible] کریں ان کے بعد بھی [illegible]
ہے میں advice کر رہی ہوتی ہوں کہ
because نہیں کھانا جا کر کے nutritionist
دوسری ہے obesity میں [illegible]
چیزیں بھی ہیں صرف یہی چیزیں کرنے سے Patient
بہتر نہیں ہو جاتا تو multidisciplinary approach لے کر
چلتی ہوں

ٹھیک ہو گیا تو آپ کو اس طرح سے جیسے Counsel
کرنے کے دوران یا اس طرح سے کبھی challenges
کا سامنا کیا کبھی آپ کو ایسا ہوا کہ یہ challenge
ہے nutrition care میں تو اس پہ تھوڑی سی
روشنی ڈالیے گا please

اس کے اندر challenges in some Patient
ہاں (ہیں Patient denial Phase میں
ہوتے ہیں جیسے ہم تو تھوڑا سا کھاتے ہیں
ہم تو صرف noodles کھاتے ہیں ہم باقی
تو کچھ اور نہیں کھاتے chocolate کا intake
تو اتنا نہیں ہے ہم تو bakery item کھاتے ہیں تو
اس قسم کے Patient related challenges کی بات کر رہی ہیں
کیا پوچھنا چاہ رہی ہیں

جی جی کسی بھی طرح کا challenge چاہیے I
ان کی جو ہے Patient related ہو یا
challenges کسی بھی طرح کا یا کسی language barrier
کے لئے تو ہمارے پاس ہوتی ہیں language barrier M
ہیں کوئی نہ کوئی staff آ جاتا یا اٹینڈنٹ attendant

Date ________

ہوتے ہیں باقی اِس لحاظ سے Patient related جو
لوگ Patients بہت زیادہ posh family سے
ہوتے ہیں They are usually academic نہیں صرف
لحاظ سے ایسا کوئی فرق پڑتا ہے اور Acne
تو ہمارے جو Patients کو بھی تھی اور Acne prone skin
میری cousins کو بھی ہے وہ تو یہ کھاتے ہیں
کچھ cousins یہ کھاتی ہیں اُن کو تو کچھ
نہیں ہوتا اور ہم کھاتے ہیں تو ہمیں کیا فرق پڑے گا
اِس لحاظ سے

صحیح اچھا مجھے Please یہ بتائیے گا کہ آپ کے I
خیال میں ایک nutrition related counselor کا
background کیا ہونا چاہیے اُس کا counseling
کیا ہونا چاہئیے

اچھا خاص qualified ہونا چاہئیے اُن کے پاس M
کم سے کم MS ہو کوئی Degree ہو کسی
Institute سے Proper یعنی nutrition میں
سے پڑھی ہوئی ہو جو بھی difference ہوتا
ہے جو ایک doctor اور quack کے اندر
ہوتا ہے جو ایک پڑھے لکھے doctor کے اندر
اور ایک quack جو quackery کر رہا ہوتا ہے
گلی محلے کا doctor یہی difference ہے

صحیح تو آپ تقریباً اپنے کتنے Patients I
کو nutritionist کو refer کرتی ہیں
Percentage اگر میں پوچھوں تو

30 35% Patients need referral M
ہاں

صحیح تو اس میں سے کتنے لوگ جاتے ہیں I
آپکے خیال میں اور کتنے نہیں جاتے

جاتے تو 1% ہی ہوں گے صرف because M
باقی تو میرا نہیں خیال کہ اتنا زیادہ
Patients جاتے ہیں گے because ہمارے
ہاں اتنا زیادہ رواج نہیں ہے نا یا قوم جو
ہے جو چل رہے ہیں ویسے چلتا ہے۔

Date

واپسے جاتے رہے

I ۔۔ جو نہیں جاتے اس کا آپ کا کوئی ہے اندازہ

کیوں نہیں جاتے ہوں گے وہ وجوہات کیا ہوں گی

R Probablly [illegible] بھی ہو سکتا ہے کیونکہ

doctors جو ہوتے ہیں وہ ہمیشہ follow up سالانہ پہ

کہ بلا رہے ہوتے ہیں تو یہ بھی ایک وجہ

ہو سکتی ہے کہ مطلب دوسری یہ بھی ہو سکتی ہے

کہ وہ کہتے ہیں نہیں ہم نے Lollars لگایا

ہے ہمارا [illegible] we were doing better we feel quite [illegible]

تو پھر ہم نے visit نہیں کیا اور اب

ہم [illegible] کر لیتے ہیں ۔۔ ہم اپنی walking

کم لیتے ہیں تو we think we don't need

a dietitian review also for it and we are

following your directions

تو جو آپ نے کہا تھا ۔۔ ample amount of water

تو سب تو we don't think we need a dietitian

Some people do visit also

مگر وہ 1-2% ہیں جن کا record بھی

میرے پاس آ جاتا ہے کہ ہاں انہوں نے visit

کیا تھا اس hospital میں یا کہیں پر بھی

[illegible] کا وہ جا رہے ہیں جی انہوں نے بھی

diet chart دیا ہے

I صحیح آپ کے حساب سے جو ایک field

ہے جیسے medicine ہے اس میں کتنی significance

ہے nutrition care کی یا nutrition counseling کی

M بہت زیادہ اچھا میں ایک ۔۔ بات بتانا بھول گئی

diabetic patient کا 100% turnover ہے وہ لوگ تو

جاتے جاتے ہیں جو جو diabetic patient میرے پاس

آتا ہے ۔۔ diabetes related disease ہوتی ہے

تو ان سے میں کہتی ہوں اگر آپ کا بالکل [illegible]

اسی طرح control نہیں رہے گی بیماری ایسے

آ رہی ہے کیونکہ یہ sugar کی بنا پر آ رہی ہے

تو وہ تو 100% ۔ انکا 99% چلو 100%

Date ________

کہتے ہیں کہ 99% Patient کا turn over
ہے کہ جب dietitian کے پاس جاتے ہیں sorry
ایسا کیا question کیا تھا
جی میں آپ سے یہ پوچھ رہی تھی کہ nutrition care T
کے حوالے سے آپ کے خیال میں جو medicine
دیا جاتا ہے اس میں کتنی significance ہے
اس کی کتنی importance ہے
بس بہت important ہے bread & butter M
ہے اگر یہ آپ اس طریقے سے صرف ہم ہی
لے کر چلیں گے multidisciplinary approach نہیں ہوگی
تو Patient failure میں چلا جائے گا
اور اگر Patient response اتنا اچھا نہیں کرتا
میں بھی counseling چھوڑ دوں تو Patient
تو مجھے پتا ہے کہ they are not going to
اگر میں بھی counseling چھوڑ nutritionist
دوں تو ہم تو definitely failure to response
ہوں گے towards treatment most of the people
لیکن آپ کے خیال میں ایک doctor I
زیادہ بہتر counseling کر سکتا ہے یا ایک
dedicated person جن کا background ہی nutrition care
dedicated کیونکہ یہ زیادہ Person والا background M
ہے related patient کی Specialist ہوتی ہے
جیسے میں اگر dermatologist ہوں تو میں orthopedic
related تھوڑا بہت تو Patient کو guide
کر سکتی ہوں زیادہ نہیں کر سکتی اور جیسے dietitian
ہیں وہ maximum ان کو دیں گی آ کر detail
دے گی ان کو بتائے گی کہ کیا کھانے کیلئے ضروری ہے
کس کے اندر کتنا ہونا چاہیئے Portion Control کرنا Calories
caloric Control کرنا ہے تو definitely اس کا Impact
ہے بہت زیادہ
لیکن آپ کا کبھی community میں جا کر I
nutrition care کے حوالے سے لوگوں سے ایک ساتھ
consultative group میں بات کرنے کا موقع ملا ہے آپ کو یا

Date ________

آپ کر رہی ہیں اس طرح سے
نہیں جی۔ M
ٹھیک ہو گیا اس کو ان Practices کو T
nutrition care کو improve کرنے کیلئے اس طرح
suggestion آپ دینا چاہیں گیں جس سے یہ
Practices improve ہوں
نئی طریقے سے campaign ہو سکیں ہیں nutrition M
television یا اس طریقے سے campaign چلائیں جو TV
پہ بھی آئیں اور جو بڑے تر جو mass population
دیکھے کہ ہاں یعنی اچھا یہ بھی کوئی topic ہے
اور اچھا اس topic پر بھی کام ہو سکتا ہے
یا اس سے relate ہمیں بھی جانا چاہئے تو
اس طریقے سے nutritionist جو ہوتے ہیں
وہ بس ایک hospital based میں دیکھا ہے
اسمیں ہوتے ہیں ان میں کوئی campaign
نہیں ہوتی ہے کوئی TV mass media کے اندر
اندر ایسی کوئی چیز نہیں آتی جتنا میں نے
دیکھا
ٹھیک ہے اور آپ کے خیال میں جیسے Patient T
جا رہا ہوتا ہے Technician کے پاس بھی جا
رہا ہوتا ہے Interaction nurses سے بھی ہوتا ہے
doctor's Interaction ہوتا ہے کس level پہ جنہیں
nutrition care کو advocate کرنے کی زیادہ ضرورت ہے
Hospital کے اندر دیکھا جائے تو doctor کے M
اوپر کے level کے doctor یا ان کے level کے اوپر کہ وہ آ کر
doctor کو بتائیں گیں کہ اس doctor Patient کو
calorie control یعنی تھوڑا بہت guide کو بھی doctors
کریں اور Television کے اوپر بھی جو عام Population
دیکھتی ہے
مطلب آپ کہنا چاہ رہی ہیں کہ جو ہے nutritionist T
جا کر doctor اور Television کو کہے پہ کہے
پہ یہ مقصد ہے
ہاں nutritionist, Television پہ کہے doctors کو M

Date ____

کو یا staff کے اندر کوئی ایسی کسی setup
کے اندر جس کے اندر nutritionist بھی ہوتی
ہیں تو دو nurse related doctors ایسا
کوئی monthly یا 2 week یا bimonthly
یا اس طریقے کی کوئی meeting ایسی
ہوتی چاہیئے کہ جس میں وہ nutrition
staff کو doctors کو وہ awareness دیں
کو

I صحیح ٹھیک ہے اور اسمیں آپ کچھ
اپنا suggestion اور کچھ add کرنا چاہیں گیں
آسمیں nutrition care کے حوالے سے

M Home کے setups میں بھی ہے لیکن اس طریقے کے
ہونی چاہیئے اگر multidisciplinary hospitals
ہے اس کے اندر تو definitely monthly
discussions کے meetings, morning meetings
یہ ساری چیزیں بہت ضروری ہیں
کیونکہ nutrition, nutrition definitely
what ever we are calling it helps
and a lot of impact in our daily life
in our own body also
تو یہ بہت ضروری ہے

I صحیح ہے Thank you so much for your time.

M You welcome thank you

I اللہ حافظ

M اللہ حافظ
