# Supplementary material for: Qualitative exploration of comprehension and experiences of healthcare professionals regarding nutrition care in Karachi, Pakistan
Source: PLOS Glob Public Health. 2025 Dec 30;5(12):e0005483. doi: 10.1371/journal.pgph.0005483 (PMC12753000; doi:10.1371/journal.pgph.0005483)
Supplement: S5 File — (ZIP) [file pgph.0005483.s005.zip › Doctor Female-001.pdf]

Doctor Female-001

[redacted]

I یہ جو Study Qualitatively ہم کر رہے ہیں اس میں ہم یہ
Explore کریں گے کہ یہ جو Health providers ہیں ان کا کیا
Nutrition care میں Practices اور Perceptions کے حوالے
سے اس بارے میں آپ سے کچھ سوالات کیے جائیں گے
آپکو پورا اختیار ہے کہ آپ جس سوال کا جواب دینا
چاہیں اس کا جواب دیں اور جس سوال کا جواب نہیں
دینا چاہیں گی اس سے اس Interview پر کوئی اثر نہیں
پڑے گا۔ آپکو کسی بھی وقت اس Interview کو ختم کر سکتی
ہیں آپ۔ جو بھی معلومات اس Interview میں دیں گی وہ
صرف Research paper کیلئے استعمال ہو گی جب ہم اسکی
Analysis کریں گے تو آپ کا نام پتا آپکی کوئی بھی
Identification ہم Remove کر دیں گے جو بھی معلومات ہمیں
Interviews کے through ملیں گی ہم انکو Publish
کریں گے لیکن Publication کے time پہ بھی آپکی
کوئی Information share نہیں ہو گی اور یہ Completely
Anonymous رکھا جائے گا۔ اگر آپ ان تمام conditions سے کو
Agree کرتی ہیں تو آپ Please verbally ہمیں اس
چیز کی اجازت دے دیجیے۔

بالکل مجھے کوئی Issue نہیں ہے آپ Proceed کریں۔

چلیں جی Thank you very much. Doctor

یہ بتائیے کہ آپ کیا Job کرتی ہیں تھوڑا
سا آپنے بارے میں بتائیے کہیں

میں [redacted] [illegible] میں M
ہے ایک Primary health care unit ہے اس طرح
کا وہاں پر میں OPD کی Incharge ہوں اور TB
کے TB Basically ہوں Focal person
سارے Patients میں Handle کرتی
ہوں اور باقی Medical OPD کے جتنے
بھی Patients سب کے سب وہ ہمارا یہاں
100-110 کا adult OPD کا daily patient turnover
ہوتا ہے۔

I Oh, hoo تو بہت زیادہ ہے یعنی آپ بہت busy
رہتی ہیں پورے دن میں تو جی۔ اچھا یہ

Date ______

بتا یئے کہ [redacted] Nutrition Area
کے حوالے سے آپ کی لوگوں کے ساتھ Identification
ہوتی ہے جہاں پہ آپ کام کرتی ہیں؟
M. ہوتی ہے جیسا کہ ہم Patients ہیں ان کو
کیا زیادہ تر ہمارے Patients Diabetic
ہوتے ہیں ان کے ساتھ Hypertension
ہوتی ہے کیونکہ ان کو Counsel کرنا
پڑتا ہے Drug management کے ساتھ
ساتھ Diet ایک Integral part ہے
اب جیسے TB کا Patient ہے تو ان کے بہت
سارے Misconception ہوتے ہیں ان کو
clear کرنا پڑتا ہے کیونکہ اگر diet
اگر اچھی ہو گی TB کی Patient تو وہ جلدی
Recover ہو گا Recovery Time کم ہوتا ہے
تو اسلئے بیماری تو ہوتی ہے diet کے
اہم بات۔
I: ٹھیک تو آپکا Background جو ہے وہ Medicine
میں ہے تو جب یہ یہاں پہ Counseling کی بات
آتی ہے تو Diet کے حوالے سے Counseling
کون Provide کر رہا ہوتا ہے ان Patients کو
M. Actually ہمارے پاس کوئی Proper Counselor نہیں
[illegible] بندہ [illegible] جیسا کرے اگر میرا
Patient ہے تو میری ذمہ داری ہو گی
اسکو Counseling کرنا تو یہ ہماری ذمہ داری
ہوتی ہے Doctors کی کہ وہ اسکو Counsel
کریں اور اسکے ایک Issue Problem کو Solve کریں
I. ٹھیک ابھی [redacted] جیسے اپنے بتایا کہ آپ
کے پاس ایک تو Turnover کا Load
ہے تو اس میں آپ کو کیا Problems
کیا Challenges Face ہوتے ہیں جب آپ Nutrition
کے حوالے سے بات کرتے ہیں Patients سے
M. جیسا کہ میں جب ہم بات کرتے ہیں نا تو ایک
تو ان کا Mindset نہیں ہوتا کسی بھی چیز کو

Date

پہلی بار ۹۹٪ جب آتے ہیں تو ہم اسکو سمجھاتے ہیں ورنہ اسکا ۹۰٪ ذہن بھی عمل نہیں کر رہا ہوتا Mental Illness کی Acceptance کے سلسلے میں تو پھر اسکی Complications کیسی بڑھنا شروع ہوتی ہے Faith بڑھتا ہے آہستہ آہستہ تو پھر وہ چیزوں کو سمجھنا شروع کرتا ہے لیکن اس میں ٹائم لگتا ہے اور وہ بھی تب ہوتی ہے جب Patient نے پہلی بار بھی دکھایا ہے اور Next time میں کسی Reason سے نہ آئیں تو وہ اسکو کوئی اور Doctor مل جاتی ہے تو وہ Treatment دے یا نہ دے سمجھائے نہ سمجھائے یہ بیچ میں Hinderness آتی ہیں ہمارے پاس۔

T. اور اسکا آپکے خیال میں کیا طریقہ ہو سکتا ہے کہ اس Problem کو کس طرح سے حل کیا جا سکتا ہے

M. ہمارے پاس Proper ایک بندہ ہونا چاہیے جو خاص طور سے ان چیزوں کی Counselling کرے۔

T. صحیح اور اسکا Background کیا ہونا چاہیے آپکے خیال میں اس بندے کا۔

M. اسکی ایک تو Sound knowledge ہونی چاہیے کم از کم Courses ہوں جو Lessons کرنے والا ہو Sound knowledge اسکی Sound knowledge ہو آجکل Academic qualification ہو obviously (Student) اسکی تو وہ سمجھتا ہے اور دوسرا وہ Patients کے ساتھ Empathetically ہو انکے مسائل کو سمجھے انکے lead پہ آکے بعض اوقات ہم لوگوں کے Rude behaviour ہو جاتے ہیں اتنا load ہوتا ہے Patient ہم بھی تھک جاتے ہیں ہم بھی کبھی کبھار Rude ہو جاتے ہیں

I تو وسائل ہو کم از کم اور دوسرا یہ کہ اگر ایک ہی اگر Counsellor ہے تو وہ 3-4 لوگ جمع کرکے ایک Time Session کرے تاکہ اسکا بھی آسان ہو اور Patients کے لیے بھی آسانی ہو کیونکہ Group کے Form میں بھی Counselling ہو رہی ہے

CamScanner

Date ________

I — فہیم، فلیحہ: اچھا یہ بتائیے کہ Nutrition Care
جو ہے جیسا آپ نے گزرا ہے Setup کی بات کروں
آپ TB Focal Person TB ہیں
کے حوالے سے دیکھیں Nutrition Care کی کیا
Significance ہے آپ Area... جہاں پہ
کام کر رہے ہیں وہاں پہ Nutrition Care کو آپ
کس طرح سے دیکھتی ہیں کتنی کس حد تک
رائٹی Significance ہے اپکی Field کے
حساب سے

M — بہت Significance ہے TB کے Patients کے اندر
کیونکہ Patient کو جو TB ہوتا ہے ایک تو
وہ Malnourished ہوتا ہے مگر اکثر لوگ جو
ہوتے ہیں لوگوں کی Myths ہوتی ہیں
گوشت نہیں کھلانا، دودھ نہیں پلانا، کیلا نہیں
کھلانا تو وہ Nutritionally deficient ہو جاتے ہیں کافی چیزیں
ہوتی ہیں تو ہم ان کو Educate کرتے ہیں اسکی
[illegible] لگتا ہے Time میں بھی Recovery time لازمی نا
ہر چیز اسکو کہتے ہیں کھلائیں unless اور
کوئی Illness نہ ہو جیسے Diabetic, Hypertensive نہ ہو
کوئی ایسی چیز نہ ہو کہ جس کی وجہ سے منع
ہو وہ گوشت منع کر دیتے ہیں کہ بالکل بھی
گوشت کھانا ہی نہیں ہے ایک چھوٹا بچہ
ہے میرے پاس آیا ہوا تھا 8-9 سال کا تھا
اب اسکو جو ہے انھوں نے منع کر دیا کہ
دودھ نہیں پلانا میں نے کہا اسکو دودھ نہیں
دیں گے تو کیا ہوگا کہتے دودھ سے منع کر دیا
کیونکہ TB بڑھ جائے گی۔ اسکو دودھ منع کر دیا
دہی منع کر دی کھائی نہیں ٹھیک ہو گئی
ٹھنڈی چیز ہوتی ہے تو یہ Problem ہوتی
ہے لیکن ہم اسکو اپنے Myths کو بتاتے
ہیں کہ یہ بھی Myths ہیں اسکو ختم کریں اسکو
چھوڑ دیں (باقی) Follow up پہ ہمارے پاس
Patients 2 weeks بعد یہ آیا ہے اسکا weight [illegible]

[illegible]

Assess کر لیتی ہیں weight gain سے۔ بس Idea
ہو جاتا ہے اور اس کی Overall looks سے ہی پتہ
لگتا ہے بھی Idea ہو جاتا ہے

I ٹھیک۔ صحیح اچھا یہ بتائیں کہ یہ جیسا اب
آپکا ایک Medical کا Background ہے تو
جب آپ نے Professionally اپنی Field میں کام
کرنا شروع کیا تھا تو Nutrition کے حوالے
سے آپ نے کوئی Specialized training کی تھی یا
کس طرح سے آپ نے Nutrition کی کس طرح
سے اپنے skills develop کیں لیکن

M Post graduation میں ہوتا ہے جب ہم Actually
کر رہی ہیں نہ تب ہم لوگوں کا ہوتا ہے Exam
ہوتا ہے نہ ہمیں آخری level تک جانا
ہوتا ہے ٹھیک ہے جب بھی ہم کوئی long case
کر رہے ہوتے ہیں کوئی Patient prepare کر رہے
ہوتے ہیں تو ہم ہماری ذمہ داری ہوتی ہے
کہ ہم نے اُسکی counseling کرنی ہے تو اس
پہ اور Examiner ہمیشہ پوچھتے
ہیں کہ اسکی counseling کرو CKD کا
Patient ہے اسکی counseling کرو تو اس لیے
ہمیں پتا ہے کہ ہم نے کیسے counseling کرنی
ہے کہ جیسے کوئی Patient warfarin پہ
ہے تو اسکی counseling اپنی ہے
ایسا تو آپ کو ہر جگہ آپ کو Dietitian
نہیں ملے گا کوئی Counselor نہیں ملے گا
تو یہ کام آپ ہی کا ہے کہ جو Doctor
اچھا Doctor ہے اس کو ایک اچھا Counselor
بھی ہونا چاہیے

I Hmm... ٹھیک۔ لیکن ابھی جیسے آپ نے کہا کہ
اب لوگ overburdened ہوتے ہیں OPDs میں اتنا
maybe اس کے پاس time constraints ہوتا ہے جو ابھی اپنے
Doctor mention کیا تو آپ کا کیا خیال ہے کہ ایک
اگر Nutrition counseling کرے گا وہ زیادہ بہتر ہے یا ایک

Date ____________

Background کا Nutrition کا جو کہ جس کا Dedicated Person
ہے جو زیادہ ہو اگر وہ Counseling کرے گا وہ زیادہ
Effective رہے گا۔

M پہلے تو جو dedicated person ہے وہ زیادہ بہتر کرے گا
ہے اس کے پاس زیادہ بہتر Knowledge ہو گی
اب ہم تو ایک Superficial سی بات کر رہے ہیں
نہ ادھر ادھر سے وہ تو detailed بات کرے
گا detailed بات سمجھائے گا

I صحیح صحیح

M جیسے کہ ہم Glycemic Index اور شوگر صحیح سے سمجھا
نہیں سکتے ہیں جو ایک Qualified بندہ سمجھا
سکتا ہے

L صحیح صحیح ان کا کیا خیال ہے Nutrition Care کا اگر ہم
جیسے پاکستان ایک Resource Limited country
ہے جیسا آپ نے بھی ابھی Resources کی بات کی
کہ Time کی کمی ہوتی ہے یا Patient Flow
اتنا زیادہ ہوتا ہے اس میں جو ہمیں challenges
ہیں ان کو آپ کیا Suggestion دیں گی As a health care provider
ہے کہ ہم کس طرح سے Effectively ان کو address
کر سکتے ہیں اپنی صورتحال کو دیکھتے ہوئے
کہ ہمارے پاس Resources کی بھی کمی ہے ہم
کس طرح سے ان کا کیا خیال ہے کہ کیسے اس چیز
کو overcome کیا جا سکتا ہے Nutrition Care کس
طرح بہتر بنایا جا سکتا ہے

M مختلف کوئی Sessions کر لیں یا اس میں بہت ساری
Lady health workers کو Involve کر لیں اس طرح سے ہم
کر سکتے ہیں کیونکہ اتنا تو Possible نہیں ہے
کہ Basic Primary health unit کے اندر Qualified dietitian ہم دے دیں
یا Nutritionist ہم دیں یہ تو Possible ہی نہیں
ہے تاکہ اس طرح ہو سکتا ہے اگر پورے Town
کے اندر بھی اگر ایک qualified بندہ ہے
ایک qualified dietitian ہے تو وہ ایک دو Sessions
کر لے ہفتہ میں ایک آدھ session کر لے اس

Date ________

Town کی Health workers ہیں ان Health workers کے
ساتھ ایک session کریں تاکہ اگر وہ Health workers کو
ہمیں مدد دے دیتی ہیں educate کرنے کیلئے ہمارے ساتھ
OPD میں ہم اپنے ساتھ Health workers کو اپنے ساتھ بٹھا
لیں ان کو train کر دیں تھوڑا بہت کہ ذرا Basics
سمجھ جائیں اور وہ اگر یہاں سے بات ہمیں سمجھ میں
نہیں آتی تو town level پہ اگر ایک جگہ Posted
ہوں گے تو وہاں ہم بھیج دیں

I سمجھ Hmm تو آپ یہ کہہ رہے ہیں کہ یہ [illegible] ہے
ہمارے اگر جو Resources کو دیکھا جائے تو اس Students
میں بھی Double ہے

M دیکھیں Double ہے Town پر attention میں ایک ایک
Town پر رکھ سکتے ہیں اگر Nutritionist یا Dietitian
میں ایک ہے ایک town میں اگر 2 کی Lady workers
ہے وہ ان کے مختلف Sessions کرے وہاں کے Area
کے ہمارے Health workers کے تو وہ جو ہے نہ ان کو Help
کریں گے بلکہ Issue بھی کرے نہیں کہ کس جگہ پہ
کو پتا Issue آ رہا ہے کہ کس Area میں کونسا
Issue آ رہا ہے

I صحیح صحیح

M وہ Health workers کی بات مانیں گے ہماری نہیں
کیونکہ ان Health workers پہ ان کا Faith ہوتا ہے
وہ ان کے Area کے ہی لوگ ہوتے ہیں ہمیں
میں سے نقل کر آتے ہیں تو اگر آپ ان کو Educate کریں
گے تو پورا ایک Area Educate ہو جائے گا

I صحیح صحیح اچھا یہ بتائیے کہ آپ کا خود کوئی
Experience رہا ہے community میں Nutrition Care
کے حوالے سے کام کرنے کا کبھی community میں
جانے کا اتفاق ہوا ہے آپ کو

M community میں جانے کا اتفاق ہوا ہے لیکن Nutrition
کے حوالے سے نہیں ہوا

I جی جی اس کے علاوہ [redacted] کوئی اور
Nutrition Care کے حوالے سے کوئی آپ ہماری معلومات

Date ________

میں اضافہ کرنا چاہیں گی یا آپ کی کوئی Suggestion
ہے یا کوئی ایسی اپنا چاہا ہے کہ کوئی کہ جو
Healthcare Providers ہیں ذہن میں یہ ہم آپ سے ایسا
ہیں Healthcare Providers جو [illegible]
opinion لے رہے ہیں
ایک Awareness کیا Nutrition Care کے بارے میں
کیا Perception ہوتا ہے یا کیا Experience ہے

M ہمارے جو بڑے officers ہیں جیسے کہ
Medical Superintendent ہیں DHO level کے جو لوگ
ہیں ہمیں تو پہلے انکو Educate کرنا چاہیے انکو
سمجھانا چاہیے کہ اس چیز کی Importance کتنی
ہے پہلے انکو یہ بات سمجھانی پڑے گی
تاکہ آگے لے چلیں۔

I آپکا کیا خیال ہے اس چیز کو کون زیادہ بہتر
طریقے سے educate کر سکتا ہے

M District Health Office میں Higher Officials
ہمارے [illegible]
کر سکتے ہیں کیونکہ ایک [illegible] ہیں
سارے Medical Specialist آتے ہیں اگر وہ اس
بات کو سمجھ گیا تو ہم obviously وہ سب کو
اس میں لگا دے گا اور اس line میں
Camps وغیرہ پتا چل جائے گی Importance
کر سکتا ہے Camps even کر سکتا ہے
Lady Health Workers کو Circular بنا سکتا ہے نکال سکتا
ہے کیلئے کہ سب اپنے پاس Mandatory کریں
بہت سے options ہیں ان کے پاس
انکے پاس Resources ہوتے ہیں اگر ہم
District level پہ جا کر بات کر لیں تو بہت [illegible] ہو جائے
گا

I اچھا [redacted] جیسا آپ نے کہا کہ Health Care Workers
کو یا Lady Health Workers کو Involve کریں اگر میں
آپ سے آپکا opinion لوں کہ کس level کے اوپر
ایک Medical Professional ہے ایک For Instance ایک ہے
Technician ایک Nursing staff ہے ایک Consultant
کس level کے اوپر سب سے زیادہ Nutrition Care [illegible]

Date

تو بچے نہ انکو engage کرنے کی ضرورت ہے

M کس Level کے اوپر ہمارے Local Teachers کی

کے اوپر کیونکہ وہ بہت ساری چیزوں کو نہیں سمجھتے

اور وہ اکثر جو ہیں نہ ان سے [illegible] QA دینے ابھی

OPD بھی چلا رہے ہوتے ہیں تو ان کے اوپر کرنے

کی زیادہ ضرورت ہے

I [illegible] your time Thank you very much بہت اچھے ٹھیک ہے

ایسا کوئی سوال جو آپ ہم سے پوچھنا

چاہتی ہیں

M نہیں ایسا کوئی سوال نہیں (ہنسی ہوئے) آپ لوگ

اچھا کام کر رہے ہیں بس ہم یہ چاہتے ہیں کہ یہ

Implement ہو جائے ایسا نہیں بن جائے

Problem ہو جائے گی نہیں سمجھ

ہمارے لیے آسانی ہو جائے گی

ہم مانے گے۔

I بالکل ٹھیک بات ہے we totally understand کہ

Practice کی آپ لوگوں کی [illegible]

کتنی زیادہ [illegible]

ہوتی ہیں ہم اوپر سے اگر جو Simple چیزیں

ہیں [illegible] Nutrition کے حوالے سے اور وہ بھی اگر

آپ لوگوں کو بتانی پڑ گئیں تو یہ آپ لوگوں

کا جو burden ہے وہ بڑھ جاتا ہے۔ تو

Thank you very much for your time we totally understand it

تو یہ بتائیے اگر ہم اسکو Publish کرائیں

ہیں تو کیا آپ اسکو اس کے بارے میں

جو کوئی بھی Research Results ہو گئیں اس

کیلئے Information لینا چاہیں گی آپ اگر

آپ چاہیں گی تو جو بھی ہماری Findings

ہو گئیں تو ہم آپکو Email کر سکتے ہیں

M بالکل کر دیں میں اپنا Email دے دونگی

I Thank you very much ٹھیک ہے چلیں
