# Supplementary material for: Qualitative exploration of comprehension and experiences of healthcare professionals regarding nutrition care in Karachi, Pakistan
Source: PLOS Glob Public Health. 2025 Dec 30;5(12):e0005483. doi: 10.1371/journal.pgph.0005483 (PMC12753000; doi:10.1371/journal.pgph.0005483)
Supplement: S5 File — (ZIP) [file pgph.0005483.s005.zip › Doctor Male -006.pdf]

S ہیلو اسلام وعلیکم

N وعلیکم اسلام

S [redacted] بات کر رہے ہیں؟

N جی بات کر رہا ہوں

S سر میں مس سوکینا بشیر
بات کر رہی ہوں۔ مس
فائزہ سے آپکا نمبر لیا
تھا۔ آپ کو بتایا ہوگا انہوں
نے۔

N جی جی

S سر آپ سے بات ہو سکتی ہے؟

N بالکل ہو سکتی ہے بتائیے خیریت؟

S جی اللہ کا شکر ہے سر میں
ڈاؤ (Dow) یونیورسٹی
آف ہیلتھ سائنسز میں لیکچرر
ہوں اور ضیاء الدین
(Ziaudin) سے PhD اسکولر
ہوں نیوٹریشن اینڈ
ڈائیٹیٹکس میں PhD کر رہی
ہوں۔ اور ابھی آپ سے

بات کرنے کا مقصد ہے وہ یہ
ہے کہ کوالیٹیٹیولی
ٹاپک ایکسپلور کرنا ہے یہ ہمیں
کوالیٹیٹیو ریسرچ کے تحت
کہ سیلف ٹیچنگ پیریڈ [illegible] کا
ایجوکیشن کا کیا رول / پرسیپشن
کیا ہے اور ان کی پریکٹیشنز
کیا ہیں یونیورسٹی ٹیچرز کے
حوالے سے۔ لہٰذا میں ایسے
سوالات پوچھوں گی اگر
آپ کو کسی سوال کا جواب
دینا مناسب نہ لگے تو آپ
جواب مت دیجیئے گا۔
اس سے ہمارے انٹرویو
پہ کوئی فرق نہیں پڑے
گا۔ ہمیں آپ کے 15، 15
منٹ چاہئیں۔ اور میں
جو بھی انفارمیشن آپ سے
لوں گی اس انفارمیشن کو میں
ریسرچ پیپر کے لئے یوز
کروں گی مگر آپ کا (Name)
نیم اینڈ (And) آئیڈینٹیفیکیشن
(Identification) ول (will)
(Anonymous) اینونیمس (Remain)
اور اس کے علاوہ پبلیکیشن
جب ہو گی اس میں بھی آپ
کا نیم (Name) (Anonymous)
اینونیمس رہے گا آپ جو

T) (F) (S) (S) (وا)

Date

بتائیں گے (everything will be anonymous)
ایوری تھنگ ول بی اینونیمس ۔

N صحیح ہے۔

S اور اگر آپ مجھے اس کی اجازت
دیں دیتے ہیں تو (Please) پلیز آپ
مجھے (Verbal) وربل کونسینٹ
(Consent) دے دیں۔

N جی میری طرف سے اجازت ہے

S شکریہ۔ سر مجھے آپ اپنے
بارے میں تھوڑا بتا دیں کہ آپ کونسے
ایریا میں جوب کرتے ہیں۔ اور
یہاں جب سے لگے ہیں
اور کتنے عرصے سے یہاں
ہیں؟

N آج کل تو میں [redacted]
ہسپتال میں جاب تو نہیں کرتا
صرف (OPD's) او پی ڈیز کرتا
ہوں، 2020 (Since) سنس
سے

S ٹھیک ہے۔

N اس سے پہلے میمن میڈیکل انسٹیٹیوٹ میں تھا (As a HOD) (ایز اے ایچ او ڈی) ۔ اس سے پہلے میں اسوسیئیٹ پروفیسر (Associate Professor) تھا ضیاء الدین ~~ٹیکنالوجی میں~~ میڈیکل یونیورسٹی کالج میں ۔

R ٹھیک ہے ۔ (OK)

N اور میں ابروڈ (Abroad) بھی گیا تھا مرحلے کے UK میں کام کیا سعودیہ (Saudia) میں تھوڑا سا کام کیا

R صحیح، تو آج کل آپ جو ہیں [redacted] میں ہوتے ہیں ؟

[redacted]

N جی [redacted]

R تو ڈیلی (Daily) وہاں کا کتنا ہوتا ہے پیشنٹ کا ٹرن (Turn over) اوور usually.

N یہ خیر انشاء اللہ بزی (Busy) فگر نہیں ~~ہے~~ ۲۰ - ۲۵ پیشنٹ دیکھ لیں آپ ۔

ٹھیک ہے۔ S

ویسے میرا نام [illegible] ہے، یہاں ایک سے دو ہفتے [illegible] ہے۔ N

صحیح صحیح۔ صحیح۔ تو سر پلیز یہ بتائیے گا، نیوٹریشن کے حساب سے آپ کا انٹر ایکشن ہوتا ہے لوگوں سے؟ S

جی بالکل ہوتا ہے (نیوٹریشن کے حساب سے۔ ایک تو ڈائبیٹیز (Diabetes) سے ریلیٹڈ (Related) ہوتا ہے، دوسرا (Obesity) اوبیسٹی یا کسی کوئی کنڈیشن) ہو اس کے حساب سے ہوتا ہے۔ N

صحیح جیسے آپ کا میڈیسن کا بیک گراؤنڈ (Background) ہے تو کیا کونسلنگ (Counselling) کی جب بات کرتے ہیں تو پھر وہ کون سی کونسلنگ (Counselling) پر زیادہ ہوتا ہے وہاں پر [illegible] (Abn[illegible]) کیا ایڈ کر دیا ہوتا ہے؟ S

وہاں پہ ابھی تو کوئی ڈائیٹیشن (Dietitian) نہیں ہے۔ N

صحیح S

N تو میں ریفر کر دیا ہوتا ہوں
جیسے شوگر کے مریضوں میں نرس میں
کر دیتا ہوں ~~[illegible]~~ دیتیوں۔
کسی کسی کیس میں ریفر
(Pattern) بھی کر دیا ہوتا
ہوں (outside) آؤٹ
سائیڈ۔ اگر یہاں نہیں ہیں
تو میں ہاں ہے۔ کسی سے
کنسلٹ کروائے ڈائیٹیشن
سے (Dietitian)

S ٹھیک۔ تو سر آپ کو۔

N یعنی الگ سے کوئی وہ نہیں ہے
کہ یہاں بھی اور جگہوں
بھی میں نے جہاں کام کیا
ہے ڈائیٹیشن نہیں ہوتی ہے
یہاں بھی تھی اور ضیاء الدین
میں بھی تھی لیکن ڈیپینڈ
(Depend) کرتا ہے کہ اگر آپ
ان کو کنسلٹ دیتے ہیں
تو وہ آ کے اس کو لیتی تھیں
ورنہ مریض تک نہیں پہنچتی۔

S تو سر کبھی چیلنجز فیس
ہوئے ہیں آپ کو ~~[illegible]~~
کا ڈسٹنگ بش نیوٹریشن
کیئر (Nutrition care) کی؟

N: کس طرح کے چیلنجز (Challenges)؟

S: ہمیں کسی بھی طرح کے چیلنجز، پیشنٹ (Patient) سمجھو نہ یہ رہا ہو یا جو ہے اس کے اپنے مس کونسیپشن (Misconceptions) میں زیادہ ہوں یا اس طرح کی بھی چینج چیلنجز ہے۔

N: ویسے یہ کم ہی ہوتا ہے۔ اتنا زیادہ ایکیں ہوتا۔ ہوتا ہے زیادہ تر جیسی یہ چینجز فیس کرتا ہوں جیسے اوور ویٹ (Over weight) اوبیس (Obese) اس طرح کے لوگ آتے ہیں لیکن تو وہ ریئلائز (Realize) نہیں کرتے کہ وہ کس طرح کی ڈائٹ لے رہے ہیں۔ وہ زیادہ تر اپنے آپ کو سمجھ رہے ہوتے ہیں کہ اتنا تو میں کھاتا ہی نہیں ہوں۔ مگر زیادہ تر ریئلائز (Realize) کر لیتے ہیں کہ جو ڈائٹری ریکمنڈیشنز (Dietary Recommendation) وہ فالو نہیں کرتے تو ان کو تھوڑا سمجھانا پڑتا ہے اس طرح کی میل (Meal) ریکمنڈیشنز ہیں کس طرح سے آپ نے اپنے میل کو کوانٹیٹی (Quantity) کرنا ہے۔

کو انٹنسٹی کو ایوریج (Average)
پر لانا ہے تبھی اس پیشنٹ کے اندر
بہتر ہو سکتا ہے۔ تو وہ شوگر چانچ
میں۔ زیادہ تر جو (Diabetian)
ڈائیٹیشنز ہمارے ساتھ ایسوسی ایٹ ہوتے
ہیں وہ کونسلنگ نہیں ~~[illegible]~~ کر لیتے ہیں
اس پیشنٹ کو۔ اگر ان کی کاؤنسلنگ
(Counselling) کی جائے تو اس
کا رزلٹ (Result) بھی صحیح
مل جاتا ہے۔

ک صحیح۔ سر آپ کے خیال میں
ایک کاؤنسلر (Counsellor) کا
بیک گراؤنڈ (Background)
کیا ہونا چاہئے؟ آئیڈیلی
(ideally) نیوٹریشن کاؤنسلنگ
(Nutrition counselling) کے دوران
جیسے اس کا اپنا بیک گراؤنڈ
(Background) کیا ہونا چاہیے

N بیک گراؤنڈ (Background)
یعنی کوئی ڈائیٹیشن (Dietician)
ہی ہو لازمی جو اس سیٹ اپ
[illegible] میں کام کر رہا ہو (Setup)
اور زیادہ تر (Counselling) بھی
نیز ہمیں اگر دو چیزوں میں
پتہ ہے ایک تو ڈائیبیٹیز
(Diabetes) سے ریلیٹڈ

Date ..........

اور وہیٹ ([illegible]) سے ریلیٹڈ اور جی آئی (GI) سے ریلیٹڈ (Related) ہیلتھ پرابلمز ہوتی ہیں جیسے سیلیئک ڈزیز (Celiac disease) وغیرہ۔ میں GI والے زیادہ ریلیٹڈ دیکھتا ہوں تو انکو ریفر کرتا ہوں

S: صحیح۔ تو آپ کے خیال میں اس سائن کی سگنیفیکنس (Significance) کتنی ہے آپ کی فیلڈ کے حساب سے اگر ہم نیوٹریشن کیئر (Nutrition care) کی بات کریں تو اس کی میڈیسن میں جو آ رہے ہوتے ہیں پیشنٹس ان میں کیا سگنیفیکنس (Significance) ہے؟

N: وہ تو ڈیپینڈ (depend) کرتا ہے کس کنڈیشن (condition) کے ساتھ آ رہا ہے۔ ظاہر ہے اگر کوئی میرے حساب سے آپ پوچھ رہی ہیں تو میرے خیال میں ڈائبیٹیز (Diabetes) کے ساتھ آ رہے ہوتے ہیں پیشنٹس (Patients) اور جو ہیں وہ [illegible] سے ریلیٹڈ

آ رہے ہوتے ہیں جیسے (Cardiovascular)
ڈزیز (Disease) کے متعلق آ رہے
ہوتے ہیں۔ ان کو اس کے مطلب
سے کاؤنسلنگ کرنی پڑتی ہے
کچھ انفیکشنز (Infections) کے
مطلب سے آ رہے ہوتے ہیں اور
ظاہر ہے اس میں اسی ہی قسم کی
چیزیں ہوتی ہیں اگر (Diabetes)
ڈائیبیٹیز کے متعلق آ رہے ہوتے
ہیں تو ظاہر ہے اس مطلب سے
کاؤنسلنگ کرنی ہوتی ہے کہ وہ اپنی
ڈائیبیٹیز کو کنٹرول کریں۔

S صحیح

N جی تو ویری (Vary) کرتے
ہیں پیشنٹس (Patients) تو
سپیشلسٹ کے پاس صرف
ڈائیبیٹیز یا اور بھی [illegible]
کے پیشنٹس نہیں آتے صرف
ایڈلٹس (Adults) سے ڈیلیورڈ
(Related) جو بھی ایشوز ہوں
ہوتے ہیں۔

S صحیح۔

S اور سر آپ کا کیا خیال ہے
کہ ایک ڈاکٹر بہت اچھے
سے (Counselling) کر سکتا ہے

(ڈیڈیکیٹڈ Dedicated
یا کوئی ~~(Dietetics) ڈائیٹیشن~~
ریلیٹڈ (Related) پرسن اس
کے لئے ہونا چاہیے - جس کا
بیک گراؤنڈ میں نیوٹریشن
کا -

میرا یہ خیال ہے جس چیز میں اپنی 21
اسپیسلائزیشن (Specialization)
ہوتی ہے اور جو اس کا
سپیشلائزڈ (Specialized) پرسن
ہوتا ہے - اس کی بہتر کاؤنسلنگ
(Counselling) کر سکتا ہے - لیکن
میرا خیال ہے جو جنرل فزیشن
(General physician) ہے یا جو
اینڈوکرائنولوجسٹ (Endocrinologist)
(Deal) ہے جو ڈیل کر رہے
ہوتے ہیں اس طرح کی چیزوں
کی ان کی نالج (Knowledge)
انہیں ان ~~مریضوں~~ کو ڈیل کرنے کی
کاؤنسلنگ کے طریقے پتہ ہیں -
ٹائملی (Timely) بھی تو ہونا
چاہئے کہ فالو اپ ٹائم دیں گے
ایک ٹائم ملے تو میرا اپنا
ایکسپیرینس (Experience)
ہے کہ اس کے رزلٹس آتے
ہیں آپ سمجھائیں گے لیکن اس
کے رزلٹس آتے ہیں جو
اگر آپ سمجھاتے ہیں تو اس

کے رزلٹ بھی نہیں آ سکیں گے

S بلکل صحیح۔ صحیح کہہ رہے ہیں سر آپ

N تو فیز میں تو اچھا خاصہ ٹائم لگتا ہے ہوں پیشنٹس کو

S صحیح۔ سر نیوٹریشن کیئر (Nutrition care) کو امپرو (Improve) کرنے کے لیے جیسے ہم اپنے پاکستان کو دیکھتے ہیں تو دیکھ رہے ہیں کہ اتنا آپ کے ~~[illegible]~~ لاسٹنگ لیک آف ریسورسز (like, lack of resources) ہیں زیادہ طور پہ آپ نے بتایا کہ ڈاکٹرز تو یا کوئی بھی پبلک کیئر پروفیشنل (Health care) (professional) کو شامل لگانا چاہیے پیشنٹ کے تو پھر رزلٹ آتے ہیں۔

N یہ ایک جنرل چیز ہے لیکن اس کا ریسورسز (Resources) سے کیا وہ۔ ابھی پھر پیشنٹس آ کہ مطلب کوئی (Physician) پیشنٹ (Patient) آ رہا ہے اور اس کو 15 منٹس دینے ہیں تو اس کو تو [illegible] منسلک

(counselling) پروپر انداز میں کرنی چاہیئے۔

S بالکل۔

N لیکن مجھے یہ رسپونس نہیں ملتا ہے زیادہ تر پیشنٹس سے تو ڈاکٹرز کی طرف سے شاید وہاں نہیں ملتا کہ انہوں نے دیکھا، لکھا اور کہہ دیا کہ زیادہ بہتر یہ ہوتا کیا ہے لیکن جو دوسرے طرح کے ہوتے ہیں کیونکہ ان کو ریفر کر دیتے ہیں کیونکہ خود سے نہیں بولتے۔

S مجھے [illegible] تو اس میں آپ کا کوئی سجیشن ہوگا کہ ہم اس کو نیوٹریشن لیول کو بہتر کر سکتے ہیں پیشنٹس (Patients) کے لیے۔

N پرچیز کا ایک کوسٹ (cost) ہوتی ہے ہمارا فوڈ لیٹ اپ (Set up) سسٹم ہی پاکستان کا وہ انشورنس (Insurance) بیسڈ (Based) نہیں ہے گورنمنٹ اس کو سپورٹ (Support) نہیں کرتی مہنگی ہے، تو مجھے یہ بھی

سوچنا پڑتا ہے اپنے مریض کے بارے
میں کہ میں اس کو اگر ایک اور
کنسلٹ (Consult) کروں گا تو اس
کے اوپر اس کی اور کوسٹ بھی
تو پڑے گی

S بالکل صحیح

N ٹھیک ہے نہ کوشش کرتا ہوں
جتنا میں کر سکتا ہوں کروں
لیکن کبھی کبھی ایسا ہوتا ہے بہت
ڈیفیکلٹ (difficult) ہوتا ہے
ایشوز (issues) اور کچھ پوچھے
ریکوسٹ (Request) بھی کرتے
ہیں کہ ہمیں بتائیں پھر سے ایک سے
کنسلٹ (consult) کریں۔
تو ظاہر ہے ان کو وہ آپشن دیا
جاتا ہے کہ وہ ایک دو لوگوں
کو دکھائیں سپیشلسٹ
(specialists) ان کو دکھائیں

S صحیح

N تو میں کہہ رہا تھا کہ آپ نے
پوچھا کہ اس کو کیسے امپروو
(Improve) کر سکتے ہیں تو (Ideally)
آئیڈیلی تو کوشش اگر ہر جگہ
پہ کوئی ایسا ہو تو یہ تو بات

اچھی بات ہے۔

S مطلب جب اس کو کاؤنسل (Counsel) کرے؟

N جو اس کو کاؤنسل (Counsel) بھی کرے بعد میں اس کو فالو اپ (Follow up) بھی کرے۔ جو پیشنٹ (Patient) کو ٹیچ (Teach) بھی کرے ایجوکیٹ کرے ان چیزوں کے بارے میں، بروڈر (broader) کا نٹیکسٹ میں ایڈوانس (advance) تک سمجھ پانے۔ سکریٹ بڑا مشکل کام ہے سیٹ اپ (Set up) کرنا۔ ہمارے ہاں میں جو لوگ اپنی جیب سے پے (Pay) کر رہے ہوتے ہیں ان کے لئے بڑا مشکل ہو جاتا ہے یہ کہ وہ تین چار جگہ پہ جائیں نہ جیسے اب ایک فزیشن (Physician) دیکھتا ہے پیشنٹ کو۔ وہ کہتا ہے کہ (Cardiologist) کارڈیولوجسٹ کو بھی دکھاؤ تمہارے دل کا مسئلہ لگ رہا ہے ڈائیٹیشن (Dietician) سے اور ڈائٹ سمیع کو کنسلٹ (Consult) کرو۔ اپنی کچھ چیزیں ایسی بھی ہوتی ہیں۔

S → N: میری پریکٹس (Practice) میں، میں سمجھتی ہوں کہ اکثر میرے سمجھانے سے بھی پیشنٹ نہیں سمجھ پا رہا ہوتا اس کو سمجھنا اور میرے لئے سمجھانا ڈفیکلٹ (Difficult) ہوتا ہے۔

تو آپ کا کیا خیال ہے کس لیول پر ہمیں پیشنٹ کو کانفیڈنس لیول سے ڈیل کرنا ہوتا ہے ٹیکنیشن (Techinician) کے پاس جانا ہے نرس کے پاس جانا ہے ڈاکٹر کے پاس جانا ہے کس لیول پر (Nutrition) کی طرف اور (Advocacy) ایڈووکیسی سے [illegible]

ڈیل کرنا یا وہ اس طریقے سے
سمجھ نہیں پا رہا تو ضرورت ہو
جاتا ہے اس کو ریفیر کرنا تاکہ
بات سمجھ میں آئے کہ مسئلہ
کیا ہے۔

S صحیح۔

N اور (Inability) ان ایبلٹی
کو فیس کرنا بھی چاہیئے لیکن
پر فزیشن (Physician) کو بھی جو بھی
اس سے ڈیل کرتا ہے اس نے
بھی اس کو بھی نہ کچھ کاؤنسل (Counsel)
ضرور کرنا چاہیئے پہلے سے اسٹارٹ
(Start) کی جائے (Understanding)
انڈرسٹینڈنگ یا ایجوکیشن
(Education) شروع تو ہو جائے
اس کی کمی وہاں پر۔

S سر اس کے علاوہ کچھ اور ایڈ
(Add) کرنا چاہیں گے؟
نیوٹریشنس آر (Nutritionists are)
کے حوالے سے پیشنٹ (Patients)
کی بیہائنڈ دا سینیریو آف
پاکستان (Behind the scenario of Pakistan) ؟

**N:** ویسے میرا اپنا اندازہ ہے کہ
لوگ سمجھنا چاہتے ہیں صحیح
سے مریضوں کو جو میرے
پاس مریض آتے ہیں۔
ہر طرح کے مریض ہوتے یعنی
اچھے پڑھے لکھے بھی ہوتے
ہیں ایسے بھی ہوتے ہیں جو
اتنے پڑھے لکھے نہیں ہوتے ہیں
لیکن زیادہ تر لوگ سمجھنا
چاہتے ہیں اپنی مریضوں کو
تو کاؤنسلنگ (Counselling)
کی ضرورت تو ظاہر ہے بہت ہوتی
ہے تو ظاہر ہے ٹائم (time)
چاہیے۔ اور ظاہر ہے (Setup)
سیٹ اپ ایسا ہو
جائے کہ ہر ایک کو یہ
فیسلیٹی (facility) (available)
اویلیبل ہو تو میرا خیال
ہے زیادہ تر لوگ اس کو
افورڈ (afford) کر سکتے ہیں۔
ان کے اوپر کوئی بہت زیادہ
برڈن (Burden) نہیں پڑے گا

**S:** شکریہ (Thank you so much for your time)

**N:** Most Welcome

**S:** اللہ حافظ
