# Supplementary material for: Qualitative exploration of comprehension and experiences of healthcare professionals regarding nutrition care in Karachi, Pakistan
Source: PLOS Glob Public Health. 2025 Dec 30;5(12):e0005483. doi: 10.1371/journal.pgph.0005483 (PMC12753000; doi:10.1371/journal.pgph.0005483)
Supplement: S5 File — (ZIP) [file pgph.0005483.s005.zip › Doctor Male -007.pdf]

M T W T F S S Date

Doctor Male - 007

S [redacted] بات کر رہے ہیں؟

P جی جی بات کر رہا ہوں

S [redacted] سب سے پہلے سکینہ

بات کرنے کا مقصد بتا دوں بیرا ہے

پتہ چلا کہ ہوگا آپ گھر میں

PH میں ورک (work) کر رہے ہیں

(As a) ایز آ پیڈیاٹریشن اسسٹنٹ

اینڈ (And) ایز آ (Are)

ڈائیٹیشن (Dietition) اور جیسے

آپ نے بولا ہے وہ کچھ کوئسچنز

(Questions) پوچھنے ہونگے کیونکہ

جو ہم یہیں کوالیٹیٹو (Qualitative)

(Qualitative) ریسرچ (Research)

(actually) ایکچولی کر رہے ہیں۔

مل (student) کی اسٹوڈنٹ PHD

ہیں ضیاء الدین میں تو اس

حوالے سے ہم ایک حصہ لی بھی

ریسرچ کر رہے ہیں ڈاکٹرز پر

اور میڈیکل ہیلتھ (Health care) ورکرز

جو پرووائیڈرز (Providers) ہیں

جیسے نرسز (Nurses) وغیرہ سے ان

میں تو ہم ان کی پرسیپشن

(Perception) ان کے پریکٹیشنرز

(Practicers) معلوم کر رہے ہیں

نیوٹریشن کیئر (Nutrition care)

کہ سوال ہیں۔ تو اگر آپ مجھے
اجازت دیں گے تو آپ سے کچھ
کوئسچنز (Questions) پوچھ
سکتی ہوں اگر آپ کو کنسینٹ (Consent)
دیں تو۔

R جی مجھ سے آپ بالکل پوچھ سکتی
ہیں۔ شیور (Sure)

S اوکے (OK) ٹھیک ہو گیا
(Thank you sir) مجھے آپ
کو یہ بھی بتانا تھا کہ اگر
آپ کوئی یا کسی سوال کا جواب
نہ دینا چاہیں تو اس سے
انٹرویو (Interview) پر کوئی فرق
نہیں پڑے گا اور آپ کی کوئی
انفارمیشن (Information) دی ہوئی
ہوگی جو اس میں آپ کا نام
(Name) نہیں آئے گا نہ آپ
جہاں ورک (Work) کرتے ہیں
نہ جو بھی آپ کا پرائیویٹ
(Private) سیکٹر (Sector)
میں جو بھی ہے کوئی نام
(Name) اس میں نہیں آئے گا
سب کونفیڈینشل
(Confidential) رکھا جائے گا
اب آپ پلیز (Please)
مجھے اپنے بارے میں تھوڑا

پیسا بتائیں گے ؟ آپ کہاں پر
ورک (work) کر رہے ہیں اور
کیا آپ کی ~~جاب~~ لائکنگ (like)
ڈرماٹولوجسٹ (Dermatologist)
ہے تو کہاں کہاں ~~کلینک~~
بیٹھتے ہیں (OPD) او پی ڈی
میں کتنے لوگ ~~دیکھتے~~ آتے
ہیں وغیرہ وغیرہ

R میں [redacted]
(Hospital) میں بیٹھتا ہوں
نارتھ ناظم آباد میں ڈرماٹولوجی
(Dermatology) پریکٹس کرتا ہوں
یہاں پر ڈیلی کے پیشنٹس
(Patients) ہیں وہ (4-5)
خود کو فائدہ ہیں

S ٹھیک ہے۔

R دوسرا میرا پرسنل (Personal)
کلینک ہے دار الشفاء مسلن ایجو
اینڈ جنرل کلینک وہاں پیشنٹ
سیون تا ٹین (7-15) میں ڈیلی
بیسس پہ۔

S ٹھیک ہے۔

R میں قطر ہسپتال (Qatar Hospital)

میں بیٹھتا ہوں اور نگی فلاؤن میں
ER میں بیٹھتا ہوں ایمرجنسی
(Emergency) میں

S جی

R اس میں جو ڈرامہ (Drama) کے
پیشنٹس ~~[illegible]~~ (Patients) ہوتے
ہیں وہ دیکھتا ہوں۔

S ~~ٹھیک~~ ٹھیک۔ تو آپ کا کام
کافی بیزی (Busy) ایسکیجیول
(Schedule) ~~[illegible]~~ رہتا ہے اس
کے حساب سے تو۔ تین جگہ
جیسے ہوئے ہیں تو ڈیلی بیسس
(Daily Basis) آپ کا
تینوں جگہ پر جانا ہوتا ہے یہ ایز سچ
(As such) ڈیز (days) آپ کے
ڈیوائیڈڈ (Divided) ہیں۔

R نہیں، تقریباً ڈیلی بیسز
(Daily Basis) پر جانا ~~[illegible]~~ ہے۔

S چلیں، اوکے (OK) گڈ (good)
یہ بتائیے گا آپ کا کبھی واسطہ
پڑتا ہے نیوٹریشن کیسز سے سوائے
سے؟

ڈھونڈ کر ہمارے ہے تقریباً روز ہی
سواسطہ پڑتا ہے۔ ~~جیسے~~
جیسے سکن (skin) کی
پرابلمز ہوتی ہیں جس میں
ڈائٹ ریسٹریکٹ (Restrict)
کرتے ہیں۔

S ٹھیک ہے۔ تو اس طرح کی کونسی
پرابلمز جیسے ہوتی ہیں جس میں
ڈائٹ ریسٹرکٹ پڑتی ہے۔

R کوئی فنگل پرابلمز ہوتے ہیں
فنگل ایشوز ہوتے ہیں ان میں
ریسٹرکٹ (Restrict) کرتے ہیں
ایکنی (Acne) کی ایشوز (Issues)
ہوتے ہیں ان میں ریسٹرکٹ (Restrict)
کرتے ہیں جو الرجک (Allergic)
کاز ہوتے ہیں (Causes) یہ تین
(3) بنیادی چیزیں ہیں جس
میں ہم ریسٹرکٹ (Restrict) کرتے
ہیں چیزوں کو۔

S اچھا تو جیسے آپ نے بتایا الرجک
(Allergic) پرابلمز (Problems) ہوتے
ہیں یا سکن (Skin) کے دوسرے
پرابلمز ہوتے ہیں ایکنی (Acne)
وغیرہ کے تو آپ اس میں
کس طرح کی ڈائٹ (Diet)

جو یہ پیشنٹس (Patients) کو کچھ ہیں کاؤنسلنگ (counseling) کے وقت لیا گیا ہے ہیں آپ ان کو ؟

R ان کو جو یہ نہ پروٹین ریسٹرکشن (Protein Restriction) ڈائٹ (Diet) کو کہتے ہیں مثال کے طور پہ جو یہ مٹن قورمے اسٹاپ (Stop) کرواتے ہیں جنک (Junk) کو اسٹاپ (Stop) کرواتے ہیں آئلی (oily) جو فوڈز (food) ہوتا ہے اس کو سٹاپ (Stop) کرواتے ہیں۔

S صحیح۔

R یہ دو بیسک (Basic) چیزیں ہیں جو یہ اسٹاپ (Stop) کرواتے ہیں۔ پیشنٹس (Patients) تھوڑے سے تو لیتے ہیں مگر فالو (follow) بہت کم کرتے ہیں

S صحیح تو آپ کے خیال میں کتنے پرسنٹیج لوگ فالو (follow) کرتے ہیں ریگولر کاؤنسلنگ (counseling) کو۔

R: مشکل ہے (50) ففٹی (fifty)
پرسنٹ کر دیں اس کو میکسیمم (Maximum)

S: ٹھیک ہے۔ اور شروع میں آپ سٹاپ
کروا رہے ہوتے ہیں (stop)
تو بتا رہے ہوتے ہیں کہ کتنے
منتھس (Months) کے لئے سٹاپ
کرنا ہے یہ فار (for) (stop)
دیئر ہول لائف (their)
ڈونٹ نیڈ 3 دے (whole life)
ٹو ٹیک دیز تھنگز
(They don't need to take these things)

R: انہیں لمیٹڈ ٹائم (Limited Time)
کے لئے ہوتے ہیں نہ اس کو سٹاپ
کرواتا ہوں (stop) ڈیپینڈ
کرتا ہے سکن (skin) کی (depend)
جو پرابلمز (Problems) ہیں وہ کتنے
عرصے میں سورٹ آؤٹ (sort out)
ہوتی ہیں۔ اس کے بعد لوگ
اوپن (open) کر دیتے ہیں
کہ جو فوڈ (food) لینا چاہے
لے سکتا ہے

S: ٹھیک ہے۔ اور کبھی آپ کا
واسطہ پڑتا ہے پیشنٹ (Patient)
سے کہ آپ کے گروپ (Group)
میں کاؤنسلنگ (Counselling) کی ہے

کمیونٹی بیسڈ (Community based) پہ نیوٹریشن کیئر (Nutrition care) کے حوالے سے۔

نہیں میرا کبھی واسطہ نہیں پڑا 7

صحیح۔ تو آپ لوگوں کو کوئی چیلنجز (Challenges) فیس (face) کرنے پڑے ہیں کاؤنسلنگ میں کسی بھی طرح کے چیلنجز 8

ابھی چیزوں کے حوالے سے پرابلم تو ہوتی ہے پیشنٹ (Patient) فالو (follow) نہیں کرتا۔ تو ظاہر سی بات ہے ہمارے لئے بھی ایک ایشو ہوتا ہے۔ کہ پیشنٹ بار بار آ رہا ہے۔ اس کے بعد یہ کہ ظاہر سی بات ہے کہ بتانا بند کر دیتے ہیں تین سے چار بار کے بعد کہ آپ فالو نہیں کر رہے ہیں تو میڈیسن ہی لیتے رہیں۔ کچھ لوگ تو بلکل فالو نہیں کرتے 9

صحیح تو اس کے پیچھے ریزنز (Reasons) کیا ہو سکتے ہیں جو فالو نہیں کر رہے ہوتے۔ 10

R

ڈرمہ (derma) کے حوالے سے
تو پیشنٹ کمپلائنس (Patient compliance)
بہت بڑا ایشو ہے ٹریٹمنٹ (Treatment)
سے جو ایکسپیکٹ (expect) کر رہے
ہوتے ہیں کہ جلدی سے ٹھیک
ہو جائیں سکن (Skin) کے جو
ایشوز (Issues) ہیں ظاہر
ہے کہ وہ لونگ ٹرم ہیں، ڈرمہ
(سکن) سکن (Skin) کے جو پرابلمز (Problems)
ہیں جس کی وجہ سے پیشنٹ
سلپ (Slip) کر جاتا ہے
دوسرے ڈاکٹرز سے کنسلٹ (Consult)
کر لیتا ہے۔ آپ نے یہ توضیح
اپنے (Scenario) سنیریو سے
بتا دیا ہوا ہے لیکن یہ ہے کہ
مختلف ڈاکٹرز کے اپنے
کومنٹس (Comments) ہوں گے
اس حوالے سے لیکن یہ بات
ظاہر سی بات ہے جب آپ ریسٹرکٹ
(Restrict) کرتے ہیں تو پیشنٹ
آپ کو پوزیٹو (Positive)
ریپلائی (Reply) نہیں کرتا اپنی
انسٹرکشنز (Instructions) کے
حوالے سے۔

S

صحیح تو آپ یہ کہہ رہے ہیں
آپ کو سکن (Skin) میں
چیلنج (Challenge) ہو

CS CamScanner

Date

فیسنگ ~~ہیوتا~~ (facing) ہوتا
ہے وہ پیشنٹ (Patient) کی کمپلائنس
(compliance) میں ہوتا ہے کہ آپ
ان کو نیوٹریشنل (Nutritional)
جو بھی کیئر بتا رہے ہوتے ہیں
وہ پیشنٹس (Patients)
یوجولی (usually) فالو (follow)
نہیں کر رہے ہوتے ہیں

Q جی اگر نیوٹریشن (Perspective)
پرسپیکٹو سے آپ پوچھیں مجھ سے
ہے۔

Q تو آپ خیال میں ہم اس کے
لیے ایسا کیا کیا جائے یا کیا کیا
جا سکتا ہے کہ پیشنٹس
(Patients) فالو کریں؟

R اس کے حوالے سے یہ ہے کہ ہم
میڈیا (Media) کا سہارا لیں
پیمفلیٹس (Pamphlets)
وغیرہ بنائیں، چھوٹے چھوٹے
سے پیمفلیٹس (Pamphlets)
بنیں ڈاکٹر اپنے روم میں
پرسکرپشن (Prescription) کے ساتھ
ان کو پیشنٹ کو دیں اور
(hand over) کر دیں
پیشنٹ (Patient) کے سامنے
ہی۔

کوئی چیز ہوگی تو ظاہر ہے جو رزلٹس
(Results) ہمیں اس کے اچھے ملیں
گے (ہمارے education point of view)
ایجوکیشن پوائنٹ آف ویو سے۔

S جیسے، ٹھیک، مطلب آپ کہہ رہے
ہیں کہ (Pamphlet) پیمفلٹ
وغیرہ بنائیں ان کا سہارا لیں
اور ان کو دے رہیں تو گھر
جو اگر لٹریٹ (literate)
ہوں تو وہ کر لینگے فالو اس
کو۔

R اوبویسلی (Obviously) پرمننٹ
امپرومنٹ (Improvement) آئے
گی۔ اس حوالے سے۔

S جیسے آپ کے خیال میں نیوٹریشن
کیئر (Nutrition care) کی
کتنی سگنیفیکنس (Significance)
ہے میڈیسن (Medicine) پوائنٹ آف
ویو سے؟

R زیادہ ہے، اور ڈرما (Derma)
کے حوالے سے تو بہت زیادہ ہے
کیونکہ میں نے پچھلے دو سال میں
تو میں نے خود ایکسپیرینس
کیا ہے کہ (Experience)

جب ہم پیشنٹس (Patients) کے ڈائٹ
(Diet) ریسٹرکٹ (Restrict) کرتے
کر رہے ہوتے ہیں میڈیسن (Medicine)
سے زیادہ نیوٹریشن (Nutrition)
امپورٹنٹ ہیں تو ہمیں اچھے
رزلٹس (Results) ملتے ہیں
سیکنڈ (2nd) اور تھرڈ (3rd)
فالو اپ (follow up) پہ

تکنیک۔ تو کونسی ایسی ڈیزیزز س
(Diseases) ہیں ڈرمہ (Derma) کے
حوالے سے جس میں آپ کو
بلکل پوزیٹو (Positive) ہنڈرڈ
(100) پرسنٹ (Percent) یا
ایٹی، نائنٹی پرسنٹ رزلٹ
(80 eight, ninety percent result)
مل رہا ہوتا ملے ہیں؟

بہت اچھے رزلٹس (Results) ج
ہیں جو ہمیں ڈائٹ ریسٹرکٹ
کرنا سے ملے ہیں۔

جی کونسی ایسی ڈزیز (Disease) س
ہے کہ جس میں جو ہے پیشنٹ
پیشنٹ (Patient) جو ہے
فالو (follow) کرتا ہے
جو آپ نے کاؤنسلنگ کی ہے
آپ کو بہت اچھے رزلٹس ملے ہیں

R فنگل (Fungal) ڈزیز (Disease)
سکن ایکنی (Acne) نیوٹریشن، ایلرجی
(Allergy) پروبلمز (Problems)
ہو سکتی ہیں۔

S صحیح، صحیح اور کوئی اسی طرح کے
چیلنجز آپ کو فیس (Face) کرنا
پڑتے ہوں ریلیٹڈ (Related)
نیوٹریشن کیئر (Nutrition care)

R نہیں زیادہ تر تو یہی چیزیں ہوتی ہیں
نیوٹریشن کے حوالے سے لیکن ایز سچ
(As such) تو کچھ اپنی پریکٹس
(Practice) کے حوالے سے تو کوئی ایشو (Issue)
نہیں ہوتا، نیوٹریشن (Nutrition)
سے زیادہ تو پیشنٹ (Patient)
ایکسپیکٹ (Expect) کرتے ہیں کہ
میڈیسن (Medicine) دی جلدی سے۔

S جی۔

N نیوٹریشن (Nutrition) میں ریشو ہے ذرا
لیکن اتنا نہیں ہے ایز کمپیئرڈ ٹو
(As compared to) میڈیسن (Medicine)
کے

S صحیح۔ سر آپ کے خیال میں سب
سے اچھا بہتر طریقے سے نیوٹریشن

کیسے (Nutrition care) کون پرووائیڈ (Provide) کر سکتا ہے؟

R: اوبویسلی (Obviously) ~~نیوٹریشن~~ نیوٹریشنسٹ (Nutritionist) ہی جو بیسٹ کیئر پرووائیڈ (Provide) کر سکتے ہیں کیونکہ جو وہ بتا سکتے ہیں وہ اس سے زیادہ جو ہے ~~نیوٹریشنسٹ~~ (Nutritionist) نیوٹریشنسٹ وہ ہماری ہیلپ (help) کر سکتا ہے تو ہونا چاہیے تو کسی کو ~~سٹیو~~ یہی بہتر طریقہ ہے؟

S: صحیح۔ نیوٹریشن کیئر (Nutrition care) اگر آپ اوور آل (overall) کو پاپولیشن (Population) کی بات کریں تو پاکستان کے سینیریو (Scenario) کو دیکھتے ہوئے کیا ~~ایسا~~ ایسا انکلوڈ (Include) کر سکتے ہیں کہ اس میں کچھ امپروومنٹ (Improvement) آ سکے، کچھ ایسی سجیشن (Suggestion) آپ دینا پسند کریں گے؟

R: اس کے حوالے سے ہماری جو ایکٹیویٹیز (Activities) ہیں نہ ~~[illegible]~~ سٹڈیز (studies) جو ہے نہ نیوٹریشن (Nutrition) کی وہ ڈسٹریبیوٹ (distribute) کر جا سکتیں ہے

(Top of the triangle) (Ground level)

گراؤنڈ لیول سے ٹوپ آف دا ٹرائینگل
بیسکلی (Basically) انٹش برگ
(Challenge) یہ ہے کہ جتنا اچھا آپ
ایجوکیٹ (Educate) کریں گے اتنا ہی
آپ کو انٹش برگ رزلٹ
(Result) اچھا دے گا۔ جیسا کہ
میں نے پہلے میں کہا (Education) ایجوکیشن
بیسک ٹیچنگ (teaching) ہے تو وہ
بھی ایجوکیشن (Education) کا پارٹ (Part)
ہے مطلب اوپن (Open Air) آپ جو
کمیونیکیشن (Communication) کرتے ہیں، اوپن
جو گفتگو ہے وہ ان چیزوں کو بہت
بہتر کر سکتی ہے ان دی ٹرم آف گروپس
(In term of groups)

S: صحیح۔ تو جیسے آپ نے بتایا کہ
گراؤنڈ لیول سے ٹوپ لیول تک
ایجوکیشن (Education) میں امپروومنٹ
(Improvement) کرنی چاہیے تو گراؤنڈ
لیول (Ground level) سے مطلب
کیا مراد ہے مطلب (Specify)
سپیسیفائی کریں گے تھوڑا پلیز
(Please)۔

R: گراؤنڈ لیول (Ground level) سے
تو آپ دیکھیں پمفلٹس (Pamphlets)
وغیرہ تو ٹاپ آف دا (top of the)
چیزیں ہو گئی ہیں

S: جی۔

e آپ جو بھی نہ گروپس (Groups)
بنائیں اور اس میں (educated) ایجوکیٹڈ
اور اَن ایجوکیٹڈ (Uneducated) کا فوٹو [illegible]
ان کا سیناریو (Scenario) بنائیں
اَن ایجوکیٹڈ (Uneducated) جو ہیں
ان میں آپ کو زیادہ ورک آپ (Working)
کرنا پڑے گا بنیفٹ آپ اور اوپر آئیں
ایجوکیٹڈ (educated) پیپل (people)
پھر اس کو مزید ڈیوائڈ (divide) کر سکیں
ان لئے (Under) ڈیزیزڈ پاپولیشن
اینڈ (And) (Diseased population)
نان (non diseased population) ڈیزیزڈ پاپولیشن
لئے اس میں اگر آپ ورک اپ (Work up)
کریں تو مختلف فیلڈ (field) کے
لوگ تو بہت اچھا نیوٹریشن (Nutrition)
کا (Scenario) سیناریو بن سکتا
ہے) یہ ڈزیز (disease) کے حوالے
سے ۔

5 اور اس میں (Stake holders) سٹیک ہولڈرز
کون کون ہوں گے جو سارا پروائڈ
کر رہے ہوں گے گراؤنڈ لیول (Provide)
(Ground level) سے (top level) ٹوپ لیول
تک ؟

K اگر کلینیکل اس میں جو سے سپیشل ہو
تو جو کنسرن (Concern) ڈیپارٹمنٹ
(Department) سے (State department) اسٹیٹ

ڈسپارٹمنٹ، گورنمنٹ اور پرائیوٹ
سیکٹرز (sectors) اور این جی اوز
(NGO's) سے جو ہیں۔ یہ کردار پلے
(Role play) کر سکتے ہیں۔
خاص طور پر یہ این جی اوز (NGO's) ،
گورنمنٹ (Government) اور پرائیویٹ
(Private) ادارے تو بہت اچھا رول
پلے کر سکتے ہیں۔ اس کے علاوہ جتنے ملٹی
نیشنل (Multi-national) کمپنیز (companies)
ہیں وہ بھی بہت زیادہ رول پلے
کر سکتی ہیں۔

S
سیمپل اور ان کو امپورٹینس (Importance)
جیسے آپ نے بہت اچھا سجیشن
دیا ہے۔ تو ان کو جو یہ امپورٹینس
وغیرہ جو نیوٹریشن کیئر (Importance)
کیئر کی ہے یہ کون بتائے گا (Nutrition)

t
جب یہ انویسٹمنٹ (Investment)
کریں گے اور ظاہر بات ہے اس میں
لوگوں کو ہائر (Hire) کریں گے
اس کا ایک لیول ہونا چاہیے کہ
جس لیول کے ٹیچرز آتے ہیں۔
ہم جتنا سمپل لیول رکھیں گے
ایجوکیشن میں اتنے رزلٹ آئیں
گے۔ اٹس نوٹ لائک (Its not like)
کہ نہ آپ بلا رہے ہیں اور
ٹاسک (Task) ٹیچ (teach) کر دیے ہیں
کہ

وہ تو میں بھی کہہ رہا ہوں کہ آپ
گھروں میں (Pamphlet) پمفلٹ
جا کر دیں پانچ پانچ دس دس
منٹ باہر جو یہ گفتگو کریں سٹریٹس
(Streets) پر ، جو اچھی اچھی
شاپس (Shops) ہیں جیسے سپر
مارکیٹ ہوگئی ، فاسٹ فوڈ کی
شاپس (Shops) ہوگئی وہاں
آگے آپ (Pamphlet) پمفلٹ
لگا دیں یا (Stickers) کر دیں تاکہ
لوگ دیکھیں ، بچوں کے حوالے سے
کارٹون (Cartoon) لیول (Level)
یہ چیزیں آپ (Broadcasts)
براڈ کاسٹ یا سوشل میڈیا
(Social Media) پہ کریں (WhatsApp)
پہ پروموٹ کریں اس طرح کی
چیزیں تو بہت اچھا رزلٹ
ملے گا

ک: رائٹ ، رائٹ ، تھینک یو
سو مچ ، فار یور سجیشن
(Thank you so much for your suggestion.)
اس کے علاوہ آپ کوئی سجیشن
(Suggestion) دینا چاہیں گے ؟

R: بس یہ ایک بہت بڑا اسٹیپ ہے سوسائٹی کا
کہ ہیلتھی ڈائٹ (healthy diet) جو ہے نہ

ہر ایک کے لئے سوئیز چاہیئے یہ نہیں
کہ صرف ٹاپ آف دا برینڈز
کے اس (Top of the brand) میں نے
پراڈکٹ کو (Aide) اور دیکھا
جائے کہ نہ ہر کوئی کے مثلاً سب بیلنسی
ڈائٹ (Realthy diet) چاہیئے
پوور کے (Poor) ہو یا رچ (Rich) کے
پیپول۔ اس کو ہمیں بتانے کی
ضرورت ہے کہ کون سی چیزیں اچھی
ڈائٹ ہے۔ اچھی نیوٹریشن ہے
اور جنک (Junk) فوڈ (Food)
کو کم کرنا چاہیئے، جو کہ تقریباً جو
دیکھا جا رہا ہے میرے ایکسپیرینس
(Experience) کے مطابق کیونکہ
بیس بائیس (20, 22) سال ہو گئے
ہیں مجھے پریکٹس کرتے ہوئے،
ہماری سوسائٹی میں بہت دل
مرض ہو رہا ہے ہارٹ ڈزیز
(heart disease) کے حوالے سے، میں اپنی
(Derma) بات کروں گا اگر ڈرما
کے حوالے سے تو اس کو پروموٹ
(Promote) کریں تو بہت اچھی کاوش
ہوگی ہم بھی آپ کا ساتھ دیں گے

تھینک یو سو مچ فار یور
(Thank you so much for your time sir) [illegible] شاہ سر 5.
