# Supplementary material for: Qualitative exploration of comprehension and experiences of healthcare professionals regarding nutrition care in Karachi, Pakistan
Source: PLOS Glob Public Health. 2025 Dec 30;5(12):e0005483. doi: 10.1371/journal.pgph.0005483 (PMC12753000; doi:10.1371/journal.pgph.0005483)
Supplement: S5 File — (ZIP) [file pgph.0005483.s005.zip › Nurse Female - 003.pdf]

Date [redacted] Nurse Female - 003

I اسلام و علیکم

FA السلام علیکم

I میرا نام نعیمہ طاہر اسد ہے اور میرا تعلق Ziauddin سے ہے اور ہم جو ہیں آپ Nutrition care کے حوالے سے Qualitative Research کر رہے ہیں اس کے اندر ہم Nurses ڈاکٹرز اور different ہم پتہ کر رہے ہیں کہ ان کی کیا understanding ہے Nutrition care کے حوالے سے تو ہم آپ سے اس سلسلے میں کچھ سوالات کریں گے اور آپ کو اختیار ہے کہ آپ کس سوال کا جواب دینا چاہیں اور کس کا نہیں اور ہم جس سوال کا جواب نہیں دینا چاہیں گی اس کا اس Interview پر کوئی اثر نہیں پڑے گا آپ کسی بھی وقت اس Interview کو ختم کر سکتی ہیں اب جو بھی معلومات اس Interview میں دیں گی وہ صرف Research Purpose کیلئے استعمال ہو گی۔ جب ہم اس کو Analysis کر رہے ہوں گے تو آپکا نام اور کوئی بھی Identifying things وہ Remove کر دی جائے گی اور جو بھی معلومات ہمیں ملیں گی اس پر ہم Publication کیلئے دیں گے اور اگر Time پر Publisher کے Share کی Information تو اپنی کوئی completely Anonymous رکھی جائے گی جو کہ Conditions تمام اس پر آپ اگر جائے گا Agree کو کر رہی ہیں تو آپ اپنا Please ہمیں دے دیں اجازت دیں Verbally consent ہمیں اس چیز کی

F ہاں okay

I تو میں کہنا چاہتی ہوں کہ آپ اپنے بارے میں بتائیں کہ آپ کا کیا نام ہے اور آپ کیا کرتی ہیں

F میرا نام ہے [redacted] اور میں Paeds کی department کی ward Incharge ہوں

Date

اور صبح 8 بجے سے لے کر 4 بجے تک onduty
ہوتی ہوں

آپ Nursing department میں ہوتی ہیں I
نہیں' میں اپنے کے department میں ہوتی ہوں F
ہوتا

اچھا I

designated department I mean F
جو ہے وہ ward Incharge کی ہے
Senior Nurse

okay آپ Senior Nurse ہیں I
آپ کہہ سکتی ہیں As a team leader F
ٹھیک ہے تو ایک Nutrition care کے I
حوالے سے کیا خیال ہے کہ آپ سمجھتی ہیں
کہ غذائی [illegible] جو ہوتی ہے وہ کیا
ہوتی ہے

دیکھیں جو As a Patient diets F
کیونکہ ہم Advice کرتے ہیں According
According to کرتے ہیں to Patient
No. 1 Patient's condition
According to patient's age No. 2
No. 3 According to the situation
اب کیا Hospital میں during admission
پاس کیا ہے اور آپ کیا Patient
کو دے سکتے ہیں آپ کے پاس کیا
ہے اور ہم Patient Available
کی age اور کیا ہے Age کے ساتھ
ساتھ اس کی condition کیا کہ وہ کیا لے
سکتا ہے اسی کے Accordingly ہم
Patient کو diet advice کرتے ہیں

ٹھیک ہے تو آپ جس طرح ایک I
Responsibilities ہوتی ہیں different تو ان
کے اندر آپ کا کتنا اس میں ہے تعلق
کہ آپ Patient کو Advice پہنچاتی ہے

Date

کر سکیں [illegible]

کرتیں صبح میں جب Patients کے پاس F

جاتی ہوں تو mothers سے میں پوچھتی

ہوں کہ Patient کی feeding کیسی

ہے oral ہے کتنا ہے Poor ہے

Adequate ہے یا کھاتا ہے بہت کم دوسری

چیز لے کہ Patient [illegible] کرتا ہے کہ نہیں

کرتا ہے ہم میرا کام ہے Patient سے

As a hygiene counseling کرنا ہے کہ وہ

اگر وہ feeding bottle استعمال کرتی ہیں تو bottles

کو boiling بھی کرتی ہے bottles کو کم نہیں

hygiene کتنی maintain ہے

Hand washing ہے کہ نہیں ہے bottles

boiled نہیں کرتیں کہ نہیں ۔ یا تی boil

Patient ہے کہ نہیں ہے ہم اگر

weakness یہ ہے تو وہ کیا ہے سکتا ہے

اس کو کیا Advice کرنا ہے ہم Patient

کو diet Advice کیا جاتا ہے

According to metabolism

metabolically اگر اس کے electrolytes اور

باقی چیزیں normal ہیں تو ہم بھی ایک نو Advice

کرنا ہے کہ آپ جو nutrition دے رہی ہیں

ہیں وہ ٹھیک ہے یا نہیں ہے اور اگر اس

میں electrolytes میں کمی ہے 19، 20 تک جیسے

electrolyte imbalance ہے تو سکتے ہیں electrolyte میں

CKD کے یا severe کمی ہے Potassium

Sodium ہے پوٹاشیم ہیں Potassium کم ہے یا

کم ہے تو بھی اس کے electrolytes

diet advice کرنا ہے کہ وہ کیا لے سکتی ہیں

diet میں Potassium ہمیں Potato سے

اچھی ملتی ہے Potassium ہمیں bananas سے

اچھی ملتی ہے ہم ہمیں KYD

diet advice کرنا ہوتی ہے

Date ______

KYBD means Yogurt، پھیکی banana
advise کرتی ہیں جیسے banana Standard ہوتی ہیں
ہیں جیسے Potassium ایسا مل جاتا ہے
مجھے تو Improve کرتی ہیں

تو یعنی کہ آپ ماشاءاللہ اتنا آپ کے اوپر Responsibility I
ہیں اتنی سب چیزیں آپ manage کر رہی ہوتی
ہیں تو آپ ساری چیزیں ایسے کہ ابھی diet
وغیرہ بھی وہ بھی آپ Properly مطلب آپ ہی
دیکھ رہی ہوتی ہیں اور پھر آپ کے ساتھ تھوڑی
اور بھی help کر رہا ہوتا ہے

میں جب Patients سے Patient کے Parents F
سے feedback لینے جاتی ہوں تو میرے ساتھ
night کا ایک ایک Staff

اچھا I

Plus team leaders, Sub coordinators F
اور morning کا ایک ایک Staff جیسے
morning کی team leader ہوتی ہیں
Junior nurse ہوتی ہیں اور ایک Sub coordinators
اور پھر میرے پاس ایک لڑکا Second nurse
بھی ہوتا ہے student As a helper
بھی ہوتے ہیں ہم ان سب لوگوں کے سامنے
Advice کرتی ہیں تاکہ وہ بھی سیکھیں

Hmm تو وہ ساری چیزیں وہ بھی مطلب I
آپ پھر آگے ان سے جس طرح وہ
ward کے اندر ہی ہوتی ہیں مطلب
ایسی جو [illegible] Patients وہیں پر جو
رہے ہوتے ہیں Hospital کے اندر ابھی کو
کر رہی ہوتی ہیں یا جو ویسے OPD وغیرہ
میں آ رہے ہوتے ہیں ان کو بھی آپ Advice
کرتی ہیں

نہیں صرف ان Patients کو کرتی ہوں جو F
میرے پاس Admitted ہوتے ہیں

Date

اچھا Admitted ہوتی ہیں تو پھر یہ ساری diet I
وغیرہ جو آپ Advice کر رہی ہیں پھر وہ اپنے
گھروں میں [illegible] دے کر آتے ہیں؟
نہیں Hospital کی diet بتاتی ہے Accordingly F
اچھا I
کہ اگر AG Patient کے ساتھ [illegible] ہے F
diet KYBD دیتی ہے پھر KYBD diet یہ ساری چیزیں
آ جاتی ہیں اگر کسی Patient کو disease کے
ساتھ ہے جیسے hypoproteinemia ہے یا پھر کسی
Case کی Surgery کے ساتھ ہے یا کوئی wound
ہے پھر بھی اس کو high protein diet advice،
کرتی ہے تو بھی بتاتا کہ Protein اور diet کیا کیا ہے
ملے گا، بہتر ہے ہیں Accordingly کے ساتھ آئے
ہیں انکو بتانا ہے کہ Anemia کیلئے آپ
کیسے recover کر سکتے ہیں آپ کو کیا کیا
diet چاہیے آپ کو کیا کیا diet دینی چاہیے
جس growth بھی ہو Anemia بھی cover
ہو جس سے Patient کو بھی ایسی Attractive diet
بنا کر جیسے کہ taste کے ساتھ کچھ
کھائے
یعنی کہ یہ ساری suggestions جو ہیں یہ آپ I
آپ نے بتایا یہ جو بھی ہیں جو arrange
کر رہی ہوتی ہیں ساری cooking وغیرہ کی
Patient کیلئے آپ انکو Advice دیتی
ہیں
نہیں انکو میں Advice نہیں دیتی F
انکو پھر کون؟ I
Patient to Patient یہ dietitian ایک F
آتی ہے یہاں
اچھا I
جو diets کو [illegible] کر کے جاتی ہے F
Advice بھی consultants یہ Plus
کرتے ہیں کہ یہ چیزیں دیں یہ بھی اپنے End

یہ کام کرنا ہے وہ اپنے level پر کام کر رہے ہیں

I تو آپ اپنے level پر ان کو بھی بتاتی ہیں کہ dietitian کو بھی کہ آپ یہ یہ issues ہیں

F اگر کوئی consultant کہے کہ dietitian کا visit کرا دو تو

I اچھا ٹھیک ہے یعنی کہ اپنا team work ہوتا ہے کہ اگر کوئی کیس کو ضرورت ہے مطلب consultant سمجھتا ہے تو وہ کہتے ہیں آپ کو کہ آپ بھی آپ dietitian کا visit کروا دیں اس کے بعد وہ dietitian اس کو چیزوں کو manage کر کے چیزیں کم کر کے آپ کو بتاتی ہے۔

F کچھ کو کچھ patients problematic ہوتے ہیں نہیں consultant نے بتا دیا لوگ believe نہیں کرتے ہیں یا پھر بہت سے cross questions کر رہے ہوتے ہیں لیکن تو پھر consultant, advice کر دیتے ہیں dietitian کا ایک visit کرا دیں تاکہ وہ proper plan بنا کر دے گی اور پھر آپ اس plan سے پھر اور بہتر ہو dietal issue ہو۔

I ہمم ورنہ ویسے phenomenon جو ہے وہ ہم آپ بھی دیکھ رہی ہوتی ہیں

F ہاں

I تو آپ counseling وغیرہ بھی کر رہی ہوتی ہیں مطلب ان کو ساتھ میں بتا بھی رہی ہوتی ہیں ان کو سب چیزیں

F صحیح صحیح

I صبح morning time کے اندر تو مطلب کتنا time دیا جاتا ہے ہوگا ہر patient پہ اگر آپ ان کو counseling کر رہی ہیں تو

F 10-15 min per patient

I اچھا اچھا تو آپ کو کیا مشکلات کیا پیش آتی ہیں جب آپ ان کو بتا رہی ہوتی ہیں کبھی patient کی طرف سے مطلب کوئی challenges ہوتے ہیں آپ کو اس وقت کہ جو

Date ____________

ee جو کرری ہوتی ہیں
Feeder پڑے ہوتے ہیں ٹنکے F
Hmm. I
Plus یہ کہ لوگ unmeasured پی رہے ہوتے ہیں F
ہیں وہ جو بڑے بڑے gallons آتے ہیں
اچھا I
تو ان ساری challenges کو ہمیں cover F
تو کرنا ہے - Delicate کے ساتھ بھی آ رہے ہوتے
ہیں unhygienic water dirty کے ساتھ آ رہے ہوتے ہیں
یہ ساری چیزیں ہیں counseling تو کرنی پڑتی ہیں ہم
میرا کام ہے جو میرا کام ہے as a senior nurse مجھے
کرنا ہے کوئی small follow کرتا ہے نہیں کرتا ہے وہ اسکی
اپنی wish ہے
تو ایک نیا آدمیں کیا change کرنا مطلب کرنا I
وہ ہے جو آپ suggest کر رہی ہیں
Environmental changes تو بہتر ہیں F
ہے نا ہمارے کراچی کے اندر نہ پانی صاف ہے
نہ آپ کو environment hygiene wise کوئی باہر
ہے جیسے بچے باہر کھیل رہے ہیں آپ کو کیا
پتا ہے کہ اچھی جگہ کھیل رہے ہیں نہیں کھیل [illegible]
رہے ground میں Parks میں چلے جائیں
چلے جائیں تو اتنی اچھی maintenance تو نہیں ہے
نا کہ بچے آ کے آپ اٹھک بیٹھک کر کے بچوں کو چھوڑ دیں
نہیں جس طرح ہوتا ہے نہ کہ آپ سمجھتے ہیں کہ یہ I
والا یا ہماری یہ والی چیزیں Improve ہو گئیں تو
اس سے ان کی غذائی صحت اچھی ہو جائے گی
سب سے پہلے تو پانی اچھا ہونا چاہیئے اور اگر F
RO Plant وغیرہ کے پانی یا پھر hygiene wise پانی
لوگ باہر سے خرید کر پیتے ہیں تو وہ کہ ہم
نہیں پتہ کہ ادھر بھی شہر سے کیسا پانی ہوتا ہے پتا
نہیں کہاں سے بھرا ہوتا ہے کیا ہے نہیں میں تو یہ
Advice کرتی ہوں اگر وہ پانی آپ لیتے ہیں
تو اسکو boil کریں تا کہ آپ اس بات کیلئے

Date ____

ensure ہو جائیں کہ ہمیں ہم نے یہ سب کر لیا

I — تو غذائی صحت کے حوالے سے آپ کیا Advice
دیں گیں کہ یہ چیز اگر کریں...

E — کم سے کم بچے ہوتی چیزیں کھائیں یہ جو
food ایسا ہیں اس سے Avoid کریں بالکل
ہے کہ چیزیں یہ جو بازار میں بکتا ہے
ہوتی ہیں اس سے Avoid کریں۔ بچوں کو
عادت ڈالیں کہ homemade کر کے کھانا
کھائیں جتنی کم سکتے ہیں اس سے زیادہ کیا
کرنا

I — اور یہاں ہم جس طرح آپ کی Responsibilities
ہیں انکے اندر آپ کیسے کم اچھا ہے اگر
change آ جائے تو بچوں کی جو غذائی صحت
ہے غذائی صحت اچھی ہو جائے گی Improve
ہو جائے گی۔

E — definitely

I — وہ کیا چیزیں ہو سکتی ہیں

E — گھر کا کھانا کھائیں، پانی boil کر کے پیئے
اچھے environment میں رہیں، بچوں کا ٹائم
ہے اس یہ چھوڑیں اب Adults میں میں
تو کام نہیں کر رہی ہوں تو کہ میں Adults میں
بھی یہ باتیں بتاتی ہیں میں تو بچوں میں کام
کر رہی ہوں۔ This is very difficult job. بچوں میں
کام کرنا اور بچوں کے Parents کو Counseling
کرنا اور پھر ان کو Convince کرنا ہر
چیز کو بتانا کہ follow کرتے ہیں جو
کرتے ہیں وہ فائدہ اٹھاتے ہیں جو نہیں کرتے
ان کی اپنی مرضی ہے

I — تو زیادہ تر لوگوں کا کیا رجحان ہوتا ہے
مانتے ہیں بات follow کر رہے ہوتے ہیں

E — ہمارے سامنے تو کر رہے ہوتے ہیں
بات کا پتہ نہیں

Date

تو کبھی ایسا ہوا ہے اتفاق کہ آپ نے کبھی community میں کسی یا personally میں آپ نے لوگوں کو چیزوں sessions کے Advice دیتے ہوں یا counselling کی ہو I

نہیں مجھے بس اپنے گھر سے [redacted] اور [redacted] سے گھر کا رشتہ ہے E

اچھا ٹھیک ٹھیک تو اس کے علاوہ آپ Suggest لگا یا ہمیں کیا مطلب solution opinion ہے یا کیا کو جو کہ آپ اپنے own پہ ڈیلے بھی improve کرنا چاہیں I

سکولوں میں سکولوں میں یہ چیزیں آنا چاہیے sessions ہونے چاہیں چھوٹے چھوٹے sessions ہونے چاہئیں کہ بچوں کو students guide کیا جائے Teachers کو guide کیا جائے اور ایک خیال میں سکولوں میں جو ہوتے رہتے ہیں اس ٹائم میں جو environment اتنا بڑے ہوتے ہیں؟ پیش کم نہیں ہوتی ہے Even آپ ایک School Saint Peter کا ایک Role کو دیکھ لیں Saint Peter آپ اچھا کر دیکھ لیں تو 10 بچے گھر رہا ہوتا ہے وہ کیسا ہوتا ہے نہیں پتہ E

hmm ٹھیک ٹھیک I

تو یہ سکولوں میں یہ sessions کرنے کی ضرورت ہے کہ بچوں کو یہ سب چیزیں بتائیں علامات ڈالیں Parents تو بتاتے ہی ہیں E

تو آپ کے خیال سے بچوں میں awareness I

بچوں میں awareness اس طرح کی ہو کہ بہت ضروری ہے کہ سکول والے دیکھیں Teachers تو اپنی بات منوا لیتے ہیں نا زبردستی ہے کہ ڈانٹ ڈپٹ کر کے یا جیسے بھی تو سلیکٹ کر جائیں اپنے School کے even میرے پاس counselors کو دیکھیں جو بچے آتے ہیں اکثر دفعہ ان کے ساتھ E

Date ______

females آتی ہیں Young child جیسے 8، 10، 12 سال
تک کی وہ Problems پیش کرتے ہیں کہ
washrooms اتنے گندے ہوتے ہیں وہاں سے
germs آ جاتے ہیں تو Health کو نہیں
سے خراب ہو جاتی ہے۔ تو ایسی چیزیں
سکولوں میں بتائی جاتی نہیں ہے نا

T ٹھیک بالکل بالکل صحیح کہہ رہی ہیں آپ
اور یہ کہ awareness جتنی زیادہ create
کریں گے

F ==پھر سکولوں میں جو water filter لگے ہوئے ہیں==
==یا نہیں یا proper filter ہوتے ہیں==
==یا نہیں کب change ہوتے ہیں تو یہ==
==ساری چیزیں دیکھی جائیں تو پروسیس [illegible]==
check & balance کوئی تو کرے

T بالکل بالکل

F میں نے دیکھا سکول میں اپنے بچے کے School
میں water filter لگا ہوا ہے پتہ نہیں کب
سے لگا ہوا ہے

T آپ کے خیال سے کون ہے ایسا انسان یا
ایسی Appropriate ہیں یا کوئی ایسی Authority
یا ایسے لوگ یا کوئی ایسی چیز جو کہ زیادہ
اچھے طریقے سے اپنا جو یہ ہے نا اپنا
خدا کی محنت کے حوالے سے کام کر سکتے ہیں

F میرا خیال میں School میں Role تو بڑے لوگوں کا
ہوتا ہے نا Principal یا Principal check کریں
وہاں کسی Teacher کی weekly duty
کسی teacher کی اس کیلئے Fix
کریں اس چیز کو check کرنے کیلئے کہ ایک
پیشہ یہ teacher کرے گا پھر یہ Teacher
کرے گا اسی طرح ساری چیزوں کا Visit کر کے
check کریں کہ کوئی کام کیسا کر رہا ہے

T Hmm ٹھیک ٹھیک

Date ______

F میرے پاس تو بچے آتے ہیں school age والے پھر new borns آتے ہیں پھر میرے پاس بچے ہوتے ہیں جو underage پیدا ہوتے ہیں بھی ہوتے ہیں underage means neonate ہم [illegible] کا سال تک کے After 1 year سے لے کر بچے میرے پاس آتے ہیں

T دیہی ایریا میں کیا زیادہ تر غذائی بیماریاں نظر آتی ہیں غذائی طور پر وہ بہت متاثر ہوتے ہیں جیسے غذائی طور

F بڑے بڑے بچوں میں تو آج کل وہ بہت زیادہ بیماری ہے نا کہ ہم نے وہ [illegible] لینی ہے جو ہمیں موٹا نہ کرے Smart بنایا ہے

T اچھا اچھا

F اور 50% آپ یہ دیکھیں بلکہ 50% نہیں اس سے زیادہ کی تعداد اس طرح کی ہوتی ہے کم لوگ زیادہ کھاتے ہیں fast food ہیں۔

T کوئی [illegible] آپ نے پہلے بھی بات کی awareness کی

F awareness کی بہت زیادہ ضرورت ہے وہ [illegible] کریں teachers کریں اور Parents کریں اور ہم کیونکہ وہ یہ سب Seminars [illegible] کریں

T Thank you so much ٹھیک ٹھیک ٹھیک آپکا بہت بہت شکریہ آپ نے ہمیں Time دیا
