# Supplementary material for: Qualitative exploration of comprehension and experiences of healthcare professionals regarding nutrition care in Karachi, Pakistan
Source: PLOS Glob Public Health. 2025 Dec 30;5(12):e0005483. doi: 10.1371/journal.pgph.0005483 (PMC12753000; doi:10.1371/journal.pgph.0005483)
Supplement: S5 File — (ZIP) [file pgph.0005483.s005.zip › Nurse Female -002.pdf]

[redacted] F S S

Nurse Female-002 [illegible]

N اسلام و علیکم

S و علیکم سلام

N میرا نام نعیمہ طاہرہ سید ہے اور میرا تعلق خیر الدین سے ہے ہم ایک ریسرچ کر رہے ہیں جو کوالیٹیٹو ریسرچ ہے جس کے اندر ہم (different) ڈیفرنٹ ہیلتھ کیئر (Health care professionals) پروفیشنلز سے ڈاکٹرز اینڈ نرسز سے نیوٹریشن کیئر کے حوالے سے ان کے کیا (thoughts) تھوٹس ہیں کیا (opinions) اوپینین ہیں کیا سجیشن ہیں اور کیا ان کی رائے ہے، اس لیے انٹرویو کر رہے ہیں۔ ٹھیک ہے؟ اس سلسلے میں ہم آپ سے کچھ سوالات کریں گے۔ آپ کو اختیار ہے کہ آپ اس سوال کا جواب دینا چاہتی ہیں یا نہیں۔ اگر کسی سوال کا آپ جواب نہیں دینا چاہ رہیں تو آپ اس انٹرویو پہ کوئی اثر نہیں پڑے گا۔ اور آپ کسی بھی وقت اس انٹرویو کو ختم کر سکتی ہیں۔ آپ جو بھی معلومات

اس انٹرویو میں (جو) آپ دیں گے وہ صرف
ریسرچ پرپز کے لیے استعمال
ہوگی۔ پھر ہم اس کا (Analysis)
اینالیسز کریں گے۔ ہم آپ کا
نام اور کوئی بھی آئیڈینٹیفکیشن
ریموو (Remove) کر دیں گے
بتا دیں گے وہاں سے۔ اور
جو بھی معلومات حاصل کریں گے
اس کو پبلش کریں گے۔ پبلیکیشن
کے بعد بھی کوئی
بھی انفارمیشن شیئر نہیں ہوگی
اور آپ کو (completely) کمپلیٹلی
اینونیمس رکھا جائے گا۔
اگر آپ ان تمام کنڈیشنز
(conditions) کو (agree) اگری کرتی
ہیں تو آپ اپنا وربل (verbal)
کانسینٹ (consent) دے دیجیے گا۔
پلیز۔ آپ اپنا کانسینٹ دے رہی ہیں؟

S جی بالکل

N کیا نام ہے آپ کا اور آپ کیا کام
کرتی ~~ہیں~~ ہیں؟

S میرا نام سائف [redacted] ہے۔
میں یہاں ایز اے (as a) ہیڈ
مڈوائف (Midwife) جاب
(job) کرتی ہوں۔ میں نے
[illegible] مڈوائف پاس کیا ہے

(F) (F) (S) (S) Date

[illegible]: (Mid wife pass out) کیا ہے (LHP) کیا ہوا ہے میں اصل میں پبلک ہیلتھ (Public Health) اسکول سے کیا ہوا ہے۔ میری یہاں پر جو جوائننگ ہے وہ 2021 میں ہے تو (As a) ایڈ ہاک مڈوائف (As a) اور نرس میں یہاں کام کر رہی ہوں۔

N: تو آپ کی جاب کے اندر کیا کام ہوتے ہیں۔ شامل کیا ہے آپ کی (Job description) جاب ڈسکرپشن میں کیا کیا کام ہوتے ہیں؟

S: ہمیں گائنی (Gynae) کے بھی (Patient) پیس پیشنٹ دیکھنے ہوتے ہیں جنرل کے جو (Patients) پیشنٹس آ رہے ہوتے ہیں انہیں بھی ہم کو دیکھنا ہوتا ہے۔

N: ٹھیک ہے تو وہ (Patients) پیشنٹس بھی آپ کو دیکھنے ہوتے ہیں تو ان کا بھی کام ورک لوڈ (Work load) ہوتا ہے؟

S: جی

N تو آپ اپنے ان پیشنٹس کو نیوٹریشن کے حوالے سے یا خوراکی معمولات کے حوالے سے کوئی ان کو گائیڈ کرتی ہیں؟

S جی بالکل۔ جیسے ہمارے پاس گائنی کی پیشنٹس (Gynae) ہوتی ہیں کوئی پوسٹ آپ (Post op) آتا ہے یا نارمل ڈلیوری ہوتی ہے اس کی تو Regular ریگولر ڈائٹ (Diet) ہوتی ہے۔ لیکن جن کا سیزر (Caesarean) ہوتا ہے اس میں ہم اس کو (soft) سوفٹ ڈائٹ (diet) ایڈوائز (advise) کرتے ہیں کہ سوفٹ جیسے دلیہ وغیرہ لیں جیسے کھچڑی وغیرہ کھالیں لیکن اس طرح کی ڈائٹ (advise) ایڈوائز کرتے ہیں ویسے یہاں (Nutritionist) نیوٹریشنسٹ ہوتی ہے وہ خود ہی بتاتی ہے مگر ہم خود بھی گائیڈ کرتے ہیں۔

N ٹھیک ہے۔ یعنی کہ آپ سے جو بھی سوالات کیے جا رہے ہوتے ہیں اور آپ ان کے جوابات دیتے ہوئے ان کی (Counselling) کاؤنسلنگ کر رہی ہیں مکمل ہے۔ تو آپ کے خیال سے کیا یہ امپورٹنٹ ہوتی ہے؟

نیوٹریشن کنفیوژ (Nutrition conf[illegible]
شدہ ہے؟

S جی جی (بالکل امپورٹنٹ) ہے
نیوٹریشنسٹ کا ٹاپک کیونکہ
زیادہ تر بیماریاں سو فیصد
ہیں زیادہ تر لوگ نیوٹریشن
(Nutrition) کا خیال نہیں رکھتی
تو نیوٹریشن کے حوالے سے
ان کو سمجھانا ضروری ہے۔
تاکہ وہ آگے جاکر اپنی ذات
[illegible] کا خیال رکھ سکیں۔

N تو یعنی کہ آپ یہ کہہ
رہی ہیں کہ بہت سے لوگ خیال
نہیں رکھ رہے ہوتے تو ان کو
خیال رکھنا چاہیے اور اس
کے لیے آپ بھی انکو سجیشنز
(Suggestions) دے رہی ہوتی ہیں
بتا رہی ہوتی ہیں؟

S بالکل۔ یہاں پر (Nutritionist)
نیوٹریشنسٹ ہوتی ہے
لیکن ہم بھی انکو بتاتے ہیں۔

N یعنی کہ (Nutritionist) نیوٹریشنسٹ
جو ہیں وہ بھی انکو بتا رہی
ہوتی ہیں اور آپ بھی انکو

بنا رہی ہوتی ہیں؟ آپ جیسے وہ
[illegible] ہیں
S تو جیسے نہیں ہیں تو بچوں کو جو بتا رہے
نہیں۔

N تو آپ کے خیال میں پرابلمز
کیا ہوتی ہیں؟ جیسے فرح آپ
ان کو بتاتے ہیں کہ اس طرح
کھائیں یہ کھائیں۔

S کیونکہ ہمارے یہاں جو پیشنٹ
(Operate) آتے ہیں وہ (Pattern)
ہو کے آتے ہیں لہٰذا ان کو تھوڑی
پریشانی ہوتی ہے۔ کہ ہمیں یہ
چیز نہیں کھانی ہوتی۔ جیسے کہ اگر
انڈے، تلی (fries) وغیرہ ہے تو
کھانے میں مصروف (Fruits) وغیرہ
نہیں کھا رہے ہیں، دودھ نہیں
پیتے، تو ان کو دالیں بھی
تھوڑا ان کو کنٹرول کرنا پڑتا
ہے۔

N تو آپ کو کیا مشکلات پیش
آرہی ہوتی ہیں کیا چیلنجز
کرنے پڑتے ہیں (Phase)
ہیں کہ جب آپ بتا رہی
ہوتی ہیں اور وہ اس کے اوپر عمل
نہیں کر رہے ہوتے

S بیں) ۔ زیادہ تر اگر سمجھا دو تو (follow) فالو کر دیتے ہیں ۔

N ٹھیک ہے، ٹھیک ہے۔ اور مطلب آپ کے ذہن میں آیا ہے کہ آپ جو بتا رہی ہو تو نہیں کہ اس طرح، اس طرح سے نئی اپنی چیزوں کو امپرو کریں) وہ فالو (follow) کر رہے ہوتے ہیں ۔

۶ [illegible] (Nutritionist) نیوٹریشنسٹ، (Advisor) ایڈوائزر کر رہی ہوتی ہے، وہی ڈائٹ جیسے ان کو آتی ہے۔ تو وہ ویسی کھاتے ہیں ۔

۷ جی بالکل ٹھیک ہے۔ تو آپ کا مطلب کبھی اس طرح سے اتفاق ہوا کہ آپ نے کبھی گروپس (Groups) میں یا بہت سارے لوگوں میں یا کمیونٹی میں آپ نے کمیونٹی کاؤنسلنگ (Counselling) کی ہو یا ان کو ~~[illegible]~~ دی ہو کہ اچھی خوراک سے لیتے ہیں یا خیال رکھنا ہے غذائی قسمت ۱۶ ۔

S: جب ہماری ٹریننگ (Training)
ہو رہی ہوتی تھی اس ٹائم
(Time) ہمیں سب کمیونٹی
میں جانا ہوتا تھا۔ تب ہم
جو (gynae) گائنی کے پیشنٹ
ہوتے تھے ان کی کاؤنسلنگ
(Counselling) کرتے تھے ڈائٹ
کے حوالے سے۔ کہ یہ ڈائٹ
پریگننسی میں لینی ہے پھر اینٹی
~~[illegible]~~ اینٹی اور اگر پوسٹ ٹرم
(Post term) میں پھر آپ کو
یہ یہ چیزیں لینی ہیں۔

N: تو پھر اس گروپ میں بہت
ساری عورتیں تھیں اور آپ
ان کو بتا رہے ہوتے تھے؟

S: جی جی

N: پھر وہ ان چیزوں کے اوپر
عمل کر رہی ہوتی تھی؟ فالو
(follow) کرتی تھیں؟

S: جی جی۔

N: اچھا اس کا مطلب ہے کہ
اچھا تھا آپ کا (Experience)
ایکسپیرینس کہ بہت اچھے طریقے

سے فالو (follow) بھی کی انہوں
نے چیزیں... ٹھیک ہے! تو اس
کے علاوہ مطلب پریگنینسی کے
دوران کے علاوہ بھی ہوتا تھا؟

S (Breastfeed) بریسٹ فیڈ مدر
(Mother) جب ہوتی تھی ان کو
بھی تھوڑا گائیڈ کرتے تھے
(Counselling) کاؤنسلنگ کرتے تھے
لیکن فالو کرنا ہے یا کرنا وہ تو
ان کے اوپر ہے۔

N ٹھیک ہے، تو پھر آپ ان
کو دیکھتے بھی تھے؟ کہ یہ کیا
کس طرح کر رہی ہیں؟

S نہیں، صرف فرسٹ (Visit) وزٹ
ہوتا تھا پھر ان کی
کاؤنسلنگ (Counselling) کر کے
ہم جاتے تھے۔

N جی، تو آپ کو پتہ کس طرح
چلتا تھا کہ اس نے صحیح عمل
کر رہی ہیں، یا فوائد فیڈ کر
صحیح نہیں کر رہی [illegible] ان کو
کس طرح پتہ چل رہا ہوتا
تھا

S: کیونکہ زیادہ نہیں کہ بیمار ہسپتال
میں وہ بتاتی [illegible]
گھر لے کے سب [illegible] ۔ تو انہیں پتہ
ہوتا تھا۔

N: اور بچوں کو مشورہ ؟ ان کو
بھی کرتے تھے ؟

S: بچوں میں نہیں، جو پریگنینٹ
مدرز (Pregnant Mothers) ہوتی
تھی ان کے پاس جاتے تھے

N: یا تو جو چھوٹے بچے ہوتے
تھے ان کی ماؤں کو ؟

S: ہاں بریسٹ فیڈ (Breast feed)
کے حوالے سے جو ان کو کاؤنسلنگ
کرتے تھے۔

N: کہ کونسی فوراک ان کو کھانی
چاہیئے۔

S: آفٹر 6 منتھس (After 6 months)
بھی ان کو کیا کیا دینا ہے
اس کی کاؤنسلنگ (counselling)
ہوتی ہے بیبی کو۔

N: ٹھیک ہے۔

R: تو اب آپ کے اس کے بارے میں کیا سجیشن (Suggestion) یا رائے دینا چاہیں گی کہ اگر ہم یہ [illegible] میں تو غذائی امپروومنٹ (Improvement) اور بھی آ سکتی ہے۔ کہ اگر ہم یہ چیزیں بھی شامل کریں۔

S. نہیں اگر ڈائٹ (diet) کا بتائیں گے، کاؤنسلنگ (Counselling) کریں گے، تو آگے جا کے ہمیں مسئلے مسائل نہیں ہوں گے، جیسے مدر (Pregnant Mother) انیمیا (Anemia) کا شکار ہوتی ہے تو یہ سے اپنی ڈائٹ (diet) کا خیال نہیں رکھیں، ہم وہ سیویر (Severe) انیمیا (Anemia) کے ایسا تو آتی ہے وہ پیشنٹ (Patient) بھر بلڈ (blood) یہ چیزیں ان کو لگتی ہیں تو اگر ڈائٹ کا خیال رکھیں گے تو ان کو یہ مسئلے مسائل پیش نہیں آئیں گے۔

R: تو ان کے لیے ہم کیا کیا امپروومنٹ (Improvement) کر سکتے ہیں؟

S کہ جب وہ اس (Duration)
ڈیوریشن میں ہوں (Pregnancy)
کہ تو وہ اپنی ڈائٹ کا
صحیح رکھیں تاکہ آگے جا کے اس
کو پرابلم نہ ہو، اس کی
ڈلیوری کے ٹائم پہ پرابلم نہ
ہو، سیزر (caesarean) کے ٹائم
پہ پرابلم نہ ہو۔ ڈائٹ اس کی
صحیح ہو گی تو (baby) بے بی
بھی اس کا صحیح ہو گا، مدر (mother)
بھی اس کی صحیح رہے گی۔

N تو اس کے اندر ہم کیا کر سکتے کہ وہ
ان ساری چیزوں کے اوپر عمل
کریں، کیا ہم اپنے سسٹم میں
کوئی اس طرح کی چیز لا سکتے
ہیں کوئی چینج سسٹم میں جس
سے ہم غذائی تحفظ کا خیال رکھ
سکتے ہیں۔

S اب یہ ہے کہ کاؤنسلنگ وغیرہ
تو کرتے ہیں، ہو سکتا ہے کہ
آ جائے نیوٹریشنسٹ جو بھی ہر
ہماری ہیں وہ تھوڑی کاؤنسلنگ
کریں تو گھر جا کے بہتر ہو سکتی
ہے ڈائٹ وغیرہ

N تو گھر جا کے ان کے اندر چینج
آ سکتا ہے۔ کہ وہ اپنی ڈائٹ

کو اچھا کریں اور اپنی زندگی
کو صحیح طرح گزار سکیں
تو اس کے علاوہ آپ اپنی طرف سے
(Suggestion) سجیشن بھی دینا
چاہیں گی؟ کہ یہ چیزیں بہتر
سے بہت بہتر سے بہتر
(Change) آ سکتی [illegible] حکومت
میں لوگوں کی۔

ک یہی ہے کہ ہم (Counselling)
کاؤنسلنگ وغیرہ کریں لوگوں
کی ڈائٹ سے میڈیسن کی
طرف نہ جائیں، ڈائٹ کے
حوالے سے زیادہ بتائیں
فوراً میڈیسن اور ان چیزوں
پہ نہ آئیں

N یعنی کہ آپ کے کہنے کا یہ
مقصد ہے کہ ان کو پہلے ایسس
(Assess) کریں اور پھر ان کی
ڈائٹ امپروو کرنے کے لیے
ان کی (Counselling) کاؤنسلنگ
کریں پھر ان کو بتائیں اور
تاکہ پھر ان کے اندر امپرومنٹ
آئے۔ تو اس کے لیے آپ کے
خیال میں کیا ہونا چاہیے کہ وہ
اس کے اوپر عمل بھی کریں

ES جیسے بچیوں کو کاؤنسل (Counsel) کرنا چاہیے کہ فروٹس وغیرہ زیادہ لیں، ملک کا ~~[illegible]~~ استعمال زیادہ کریں اور یہ ہے کہ انکو زیادہ اچھی ڈائٹ لینی چاہیے۔

N کاؤنسلنگ کر کے کیا کر سکتے ہیں ہم؟ جس سے ان میں (Improvement) امپرومنٹ آ سکتی ہے؟

S بس نہیں ہے۔

N اچھا۔ اس کے علاوہ کوئی اور تجویز دینا چاہیں گی؟

S نہیں۔

N تھینک یو!
