# Supplementary material for: Qualitative exploration of comprehension and experiences of healthcare professionals regarding nutrition care in Karachi, Pakistan
Source: PLOS Glob Public Health. 2025 Dec 30;5(12):e0005483. doi: 10.1371/journal.pgph.0005483 (PMC12753000; doi:10.1371/journal.pgph.0005483)
Supplement: S5 File — (ZIP) [file pgph.0005483.s005.zip › Nurse Female -004.pdf]

Date [redacted] Nurse Female - 004

I: اسلام و علیکم

S: وعلیکم اسلام

I: ہم PhD کے سلسلے میں ادویہ اور nutrition care اور monitoring کے بارے میں آپ سے تھوڑے سے سوالات پوچھیں گے آپ کی اپنی مرضی ہے جس سوال میں آپ uncomfortable ہیں اور آپ نہیں جواب دینا چاہتیں تو آپ بیشک جواب نہیں دیجئے گا اگر جو ہے آپ comfortable ہیں تو آپ جواب دے دیجئے گا اس کے علاوہ جو ہے یہ ہم Publish کرائیں گے آپ کا نام identity ایڈریس کچھ کہیں show نہیں ہوگا hospital میں ہیں کچھ بھی show نہیں ہوگا اس میں یہ سب interview ہم ویسے ہی [illegible] ہیں ٹھیک ہے اور اگر آپ چاہیں گی تو آپ کو آسکتا جو Result آئے گا Publication جو بھی ساری ہوگی تو وہ بھی ہم email پہ آپ کو بھیج دیں گے جیسے آپ بتا رہی ہیں۔ شروع میں پہلے اپنے بارے میں تھوڑا سا بتائیں ا ا ا نام آپ کتنے سالوں سے یہاں نرس ہیں

S: میرا نام [redacted] ہے اور مجھے یہاں چھ (6) سال ہو گئے ہیں [redacted] میں job ہے as a trainee اور Recently کر رہی ہوں ابھی میں نے RN complete کیا ہے Bachelor کیا ہوا ہے

I: ٹھیک ہے بہت اچھا okay ٹھیک ہے آپ کو کبھی ایسا ہوا کہ patient نے کبھی آپ سے پوچھا ہو یا کبھی آپ نے دی ہو یا nutrition care یا nutrition کے حوالے سے کچھ بتانا چاہیں گی آپ

S: ہمیں nutrition care یہاں پر basically dietitian جو ہوتی ہیں وہ آکر بتاتی ہیں وہ chart وغیرہ بھی بنا کر دیتے ہیں یہاں اگر پڑھتے ہیں یہاں پر پڑھتے ہیں اگر nutrition وغیرہ کیلئے تو بتاتے ہیں۔ ہم لوگ کہ mom کی deficiency کے ساتھ تھوڑا زیادہ بچے [illegible] آتے ہیں

Date

تو ہم ایک meal کی medications وغیرہ دی جاتی ہیں
کم diet وغیرہ دی جاتی ہے patients کو consultants بھی
ہمیں guide کر رہے ہیں

I ٹھیک ہے

S Consultants بھی ہمیں جو بھی چیز prescribe کرتے
ہیں medication کر لیا proper طریقے سے ہم
chart بنا کر دے کیونکہ (ہمارا بہت کم ہوتا
ہے ان لوگوں کو سمجھانا یہ نہیں کے ان
سوال پوچھتے نہیں تو ہم نہیں بتاتے ہیں)

I ٹھیک ہے جب وہ آپ سے سوال پوچھتے ہیں
meal وغیرہ کے حوالے سے تو آپ ان کو
direct کر دیتے ہیں consultant کو

S نہیں consultant نہیں consultant بھی جو
ہے patients کے اوپر ہی

I dietitian یہاں پر کتنی dietitian ہیں

S آتی ہیں تین چار ہی ہیں

I ٹھیک ہے ٹھیک ہے مطلب آپ لوگ
خود patients کو کھانے پینے
کے حوالے سے کچھ نہیں counseling کراتے
ہیں تو ... آپ کے خیال میں nutrition care
ہے کیا، اس میں کیا کیا چیزیں آتی ہیں

S nutrition care یہاں پر تو patient ہوتا
6 months کے بعد feed ہوتی ہے مطلب Paediatric
میں 6 months کے بعد start ہوتی ہے diet semi solid شروع ہوتی ہے اس میں
ہم مطلب سمجھا رہے ہوتے ہیں کہ ابھی کو یہ
کھچڑی وغیرہ جو starting ہوتی ہے

I جی تو آپ نے بتایا کہ جو بھی nutrition concerns
ہوتا ہے تو dietitian کی طرف direct کر دیا
جاتا ہے usually آپ barely ان کو counsel
نہیں کر پاتی ہوں

S جی جی

I کبھی ایسا ہوا ہو groups میں جا کر کسی
community میں جا کر آپ کو ضرورت پڑی ہو

Date

counsel آپ نے nutritionist کے حوالے سے تو
کرنا ہے تو basically focus کیا ہوتا ہے
S focus بھی ہوتا ہے آجکل (بچوں میں iron
deficiency بہت زیادہ ہو رہی ہے mothers
زیادہ تر feed نہیں کرتیں nutrition بھی feed کرانا
iron ڈبے top feed سے ویسے بھی بچوں کے iron
deficiency بہت آ رہی ہوتی ہیں تو اس پہ
زیادہ دھیان دیتے رہے ہیں) اس
کے علاوہ بھی ہم نے بہت زیادہ guide کیا کہ
feed وغیرہ کو چھوڑ کر تھوڑا سا ان
کی diet وغیرہ پہ بھی دھیان دیں کیونکہ
ان سے ان کا Hb level بھی کم آتا ہے
T ٹھیک ہے تو Age کیا ہوتی ہو گی آپ
بتا سکتی ہیں
S ہاں جی (ویسے Age بھی بتاتی ہیں اب
میں نے آپ کو بھی بتایا تھا کہ 6 months
کے بعد تو start ہوتی ہے weaning diet تو
اس کے اندر ہم mashed potatoes یا آلو
کھچڑی یا boiled egg یہ سب ساری
چیزیں بتا رہے ہوتے ہیں) کہ آپ
دے سکتی ہیں
T ٹھیک ہے اور Iron کیلئے آپ basically
آپ کس چیز کو focus کر رہے ہو کہ
ان میں Iron deficiency Anemia نہ ہو
S Red meat بھی دیں
T ٹھیک کس Age میں دینے کا کہتے ہیں
S ایک سال یا دو سال کے بچوں کو
T صحیح ہو گیا
S Red meat ہو گیا green vegetables ہو گئے
ویسے تو otherwise کیا ہے consultant
بھی ہوتے ہیں وہ کچھ diet کیلئے منع
نہیں کرتے کہتے ہیں سب چیزیں کھلائیں
T ٹھیک ہے آپ کو ایسے لگتا ہے اس میں

CamScanner

Date ______

Nutrition Care دیے میں ایسے challenges ہیں
کوئی ایسے barriers ہیں کہ رکاوٹیں
ہیں ہوتی رہی ہیں جو کسی طرح سے دور
ہو سکتی ہے کوئی ایسی رکاوٹ ہو کوئی
آپ کو لگتا ہوتی کہ patient کو
صحیح سمجھ میں نہیں آ رہا ہے اس طرح
کی کوئی چیز

S ہاں یہاں پر آ لیے ہیں (language barrier
بھی بہت آتے ہیں جنھیں اسے سمجھ نہیں
آ رہی ہوتی ہے Nutrition سے بھی
لوگ آتے ہیں جنھیں سمجھ نہیں آتی ہے
کہ ہم لوگوں کو یہ کرنا ہے وہ کرنا ہے)
یہ barrier ہے سب سے زیادہ

I صحیح

S Language barrier اور mother tongue کی ہیں
نہ تو ان کو کیا بھی سمجھاتے ہیں انھیں
Language سمجھ میں ہی نہیں آ رہی ہوتی

I ٹھیک ہے کبھی ایسا ہوتا ہے کہ آپ
کو لگتا ہے کہ dietitian کم ہیں اور
زیادہ ہونی چاہئیں

S نہیں

I نہیں enough ہی جا رہی ہیں جتنے بھی patients
ہیں patient کے لیے

S dietitian آتی ہیں اچھے سے سمجھاتی
ہیں chart وغیرہ بنا کر دیتی ہیں
جنھیں need وغیرہ سب سے زیادہ ہوتی
ہے

I صحیح ہے اس کے علاوہ اب اس کے
بارے میں کچھ کہنا پسند کریں گی
Nutrition Care کے بارے میں کوئی ایسی
چیز ہو جائے کہ یہ اور بہتر ہو جائے
Seminar ہوں یا اس طرح کی کوئی
چیز

CS CamScanner

(S)

Date

ہاں Seminars ہونے چاہئیے یہ ساری
چیزیں ہونی چاہئیے ان Seminars ہونی S
چاہئیے لوگوں کو بالکل کرنا چاہئیے
ان چیزوں کو Community میں جا کر
[illegible] local علاقے والے لوگ ہیں خاص
طور پر انہیں سمجھانا چاہئیے
اور Seminar کون conduct کروائے T
یہی جو Community کے لوگ ہوتے ہیں S
outsider basically جن کا کام ہے
کا انہیں لوگوں کو یہ Seminars conduct کروانا
چاہئیے کہ Community میں جا کر
ٹھیک T
میرا یہ point ہے S
صحیح ہے آپکو ایسا لگتا ہے کہ Training T
یہاں پر جو nurses ہیں lady doctors
ہیں اُن کو بھی Seminars conduct
ہوتے ہیں research، up to date جیسے
ٹی بی ہے اُس کے حوالے سے کچھ
یہاں نہیں ہوتے ہیں یہ نہیں کہ S
نہیں ہوتے ہیں Seminars وغیرہ classes
ہوتے ہیں تو چیزیں ہوتی [illegible]
ہوتا ہے
ٹھیک ہے اور کچھ کہنا چاہیں گیں T
کچھ نہیں S
Thank you much T
