# Supplementary material for: Qualitative exploration of comprehension and experiences of healthcare professionals regarding nutrition care in Karachi, Pakistan
Source: PLOS Glob Public Health. 2025 Dec 30;5(12):e0005483. doi: 10.1371/journal.pgph.0005483 (PMC12753000; doi:10.1371/journal.pgph.0005483)
Supplement: S5 File — (ZIP) [file pgph.0005483.s005.zip › Nurse Female -005.pdf]

Date [redacted]

Nurse Female-005

میں Phd کی Student ہوں جو nurses کے nutritional care کے متعلق I
کچھ سوالات آپ سے پوچھوں گی
پہلے آپ مجھے اپنے بارے میں بتائیں گی۔ اچھا
کوئی سوال آپ کو ایسا لگے کہ آپ
کو نہیں بتانا آپ کو نہیں ہے تو بیشک
چھوڑ دیں وہ سوال آپ کو نہیں
کرنا ہے آپ بالکل، پھر جو بات
آپ کہہ رہی ہوں کہ آپ اس کا جواب
نہیں دینا چاہتیں ٹھیک ہے تو جیسے میں نے
آپ کو بتایا تھا کہ جیسے ہم Patient
کی ریکارڈنگ کے لیے اس چیز کو آپ کا نام
نہ کوئی اور چیز نہ آپ کی ریکارڈ کر رہی ہیں
لیکن کوئی ایسی چیز اس میں نہیں ہے
تو ٹھیک ہے اچھا آپ مجھے یہ بتائیں
اپنے بارے میں بتائیں اپنا نام آپ
کتنے سال سے یہاں پر ہیں کون سے
میں ہیں وغیرہ وغیرہ

میرا نام [redacted] ہے اور میں یہاں سے ہر Z
یہاں Paeds میں 2 سال سے ہوں

ٹھیک ہے a ... I

As a nurse Z

As a nurse اپنی basic knowledge آپ I
نے کہاں سے کیا

[redacted] سے میں نے education کی ہے Z
یہاں کی Institution میں

ٹھیک ہے آپ کو کبھی ایسا محسوس ہوتا ہے I
کہ جو nutrition care اپنی طرف سے جاتی
ہے Patient کی

ہاں کبھی کبھار تو ہوتا ہے کہ Patient Z
لیڈیز ہیں جیسے کہ ایک Patient
تھا آپ پاس nation کے ساتھ آیا ہے
اپنا diet plan اپنا treatment لے جاتے ہیں
ہم ... کی guidance لیتے ہیں اور

Date ______

خود بھی بتاتی ہیں جیسے کہ KYB diet ہو رہی ہے
ان کو یہ بھی ایجوکیٹ کر رہے ہیں ہم خود سے
ٹھیک ہے آپ کچھ خود سے بھی guiding I
کر رہی ہیں اور ان کو refer بھی
کر رہی ہیں
پہلے ہم dietitian کو ہی refer کرتے Z
ہیں اگر ان کو کچھ ہو in detail کچھ
پوچھنا ہو یا language barrier کی وجہ سے اگر
کچھ بھی تو ہم ان کو guide کر دیتے ہیں
ٹھیک ہے اگر language barrier ہو تو آپ I
تو آپ دیکھتی ہیں تو basically آپ
کیا guide کرتی ہیں؟
جیسے KYB diet کیا لانا، کھانا ہو گیا Z
جیسے yogurt وغیرہ جیسا کہ میں نے بولا
nuts کیلے کر دی بھی جیسے پیٹ خراب
ہو گیا تو اس کیلئے کیا diet
recommend کرتی ہیں، اسی طرح
ٹھیک ہے یہ nutrition care کا جب ہم نام لیتے I
ہیں تو اس میں آپ کیا کیا بتاتے
کیا کیا عناصر اس میں آتے ہیں کیا کیا چیزیں
اس میں شامل ہونی چاہیئے
جیسے؟ Z
جیسے ہم کہتے ہیں کہ آپ nutrition care I
دیتی ہیں تو اس میں کیا کیا چیزیں
ہیں counseling ہے
ہم nutrition care میں counseling بھی آتی Z
ہے اگر آپ کے سامنے کوئی patient ہے
جس کو وہ diet سوٹ نہیں کر رہی ہے
اور اس کے سامنے وہ دے رہا تو آپ اس کو
ری پلیس کرتی ہیں ہم یہ کرتے ہیں۔
ٹھیک ہے کبھی ایسا اتفاق ہوا کہ I
آپ نے کسی کو ٹوکا ہو اور آپ کے سامنے
اس طرح سے لے رہا ہو۔

CS CamScanner

Date ______

Z spice کھا رہا ہے کھا رہا ہے ہوتے ہیں
اگر بچے [illegible] Pizza کا شوق رکھتے ہیں
یا جیسے یا بہت خراب کے ساتھ بھی بچہ
آیا ہے تو Pizza کی demand کر رہا ہے
اس family کے ساتھ دے رہی ہے تو ہم definitely
منع کرتے ہیں کہ spice کم نہیں دیں

T ٹھیک ہے صحیح ہے کبھی ایسا ہوا ہے کہ
community میں جاتا ہو لڑکا ہو تو community
میں لوگوں میں ایک تو بہت nutrition دینے
بڑی ہے advice دینی پڑی ہو کبھی اس
کا اتفاق نہیں ہوا

Z نہیں

T صحیح ہے آپ کے خیال میں nutrition care
کس کو دینی چاہیئے

Z nutrition care کی تو ہر کسی کو need
ہوتی ہے ہر بندہ آپ سے پوچھتا ہے
nutrition care کے بارے میں تو یہ تو آپ
کسی کو بھی دے سکتے ہیں اگر آپ کی
knowledge میں ہے تو

T صحیح ہے بندہ best guide کون ہے اس کا

Z dietitian ہی دے

T صحیح یہ ہو گیا اب اس طرح سے دیکھا
ہے کہ dietitian کی کمی کی وجہ سے یہ
appropriate نہیں دی جا رہی ہے

Z ظاہر ہے government setup

T اپنا کبھی اتفاق ہوا کام کرنے کا

Z کام کرنے کا نہیں لیکن دیکھنے کا
ہوا ہے

T Government setup میں جیسے

Z Patient آتے کے جیسے diet
ہیں اس طرح کی diet نہیں دی
جاتی ہے

T Hospital میں نہیں دی جاتی یا

Date ______

Z — Disabled Equal طلبہ ہر کسی کو جیسے Political
Isolation کر رہا ہے یا کسی بھی وجہ اس
میں ignorance شامل ہے تو اس طرح
ہمارے Private Schools میں یہ ہوتا ہے کہ
ہر بچے ignorance کے مطابق الگ
درجہ ہے Government Schools کی سطح میں
نہیں ہوتا ہے

I — ٹھیک ہے یہاں پر Disabled کم ہوتے ہیں؟

Z — Disabled نہیں ہوتے یا نہیں پائے
جاتا Setup نہیں ہے لیکن

I — وہاں پر مسئلہ ہے

Z — مسئلہ ہے

I — پھر آپ کے خیال میں challenges
کیا کیا ہیں جیسے Autism Care کے حوالے سے
اگر ہم لوگوں میں بات کریں

Z — (Lack knowledge پھیلانا)

I — [illegible] knowledge [illegible] knowledge
دینے والے لوگ زیادہ ہونے چاہئیں
یا

Z — [illegible] Knowledge دینے والے طریقے سے ایک
Connecting کرنا چاہیئے تا کہ Awareness
کیا جائے جیسے ہم Seminars وغیرہ
(کرتے ہیں [illegible])

I — ٹھیک ہے

Z — (کوئی Platform دیتے ہیں لوگوں کو
تو وہ کرنا چاہیئے Seminars Guidance کیلئے)

I — تو کس کس کیلئے Guidance ہوتی
چاہیئے Platform کی

Z — Guidance کیلئے تو Awareness کیلئے
تو آپ Medically طرح ہر چیز کیلئے
Awareness کی ضرورت ہوتی ہے

I — صحیح ہے

Z — جیسے Covid تھا تو اس میں Awareness نہیں

Date

ہوتی تو اس میں لوگ اور زیادہ بہت مر گئے
صحیح I
awareness بہت زیادہ ہی تو اس سب مطلب Z
Covid سے اب
آپ TV پہ دیکھ رہے ہیں
کے بارے میں Precaution بتائی جاتی
ہے مطلب ہر طرح سے ایک guide کیا
جاتا تھا awareness بہت زیادہ اس لیے
ہوتی تو جب awareness آئی ہے تو
تو Covid کم ہونا شروع ہوا
ٹھیک ہے تو آپ کا یہ خیال ہے کہ I
problem اس وجہ سے زیادہ بڑھا ہے کہ
education کی کمی اس وجہ سے ہمارے
ملک میں ہے کہ لوگوں کو awareness نہیں
ہے
awareness نہیں ہے Z
اور اس کو ہم کس طرح overcome I
کر سکتے ہیں
(awareness دے کر) Z
awareness دے کر overcome کر سکتے ہیں I
ٹھیک ہے اس میں اور کچھ آپ add کرنا چاہیں
گی
نہیں Z
awareness اور education کے علاوہ اور کچھ نہیں I
نہیں as such نہیں Z
چلیں Thank you so much for your time I
آپ کے وقت کا بہت شکریہ
