# Supplementary material for: Qualitative exploration of comprehension and experiences of healthcare professionals regarding nutrition care in Karachi, Pakistan
Source: PLOS Glob Public Health. 2025 Dec 30;5(12):e0005483. doi: 10.1371/journal.pgph.0005483 (PMC12753000; doi:10.1371/journal.pgph.0005483)
Supplement: S5 File — (ZIP) [file pgph.0005483.s005.zip › Nurse Female-001.pdf]

[redacted] Nurse Female-001

I اسلام علیکم میرا نام [illegible] ہے ہم یہاں سلسلہ
ہم qualitative research کر رہے ہیں جس کے اندر ہم
different health professionals کے interviews لے رہے ہیں
اور ان سے پوچھ رہے ہیں کہ ان کا کیا concepts
ہے انکا رائے ہے malnutrition care یعنی غذائی کمی
کے حوالے سے تو وہ جو ہم انکی opinion اور conceptions
وہ دیکھ رہے ہیں تو یہ کہ ہم آپ سے اس سلسلے میں
جو ہے نا کچھ سوالات کریں گے اور آپ کو اختیار ہے
کہ آپ کس سوال کا جواب دینا چاہیں اور کس کا نہیں
اور آپ جس سوال کا جواب نہیں دینا چاہیں گی تو اسکا
اس interview پر کوئی اثر نہیں پڑے گا آپ کسی
بھی وقت اس interview کو ختم کر سکتی ہیں اور آپ
جو بھی معلومات اس interview میں دیں گی وہ صرف
Research Purpose کیلئے استعمال ہوگی جب ہم اسکی
Analysis کریں گے تو آپ کا نام اور کوئی بھی
identification remove کر دی جائے گی ہٹا دی جائے
گی اور جب اسکو Publish کریں گے تو تب بھی
Publication کے time پہ بھی آپ کی کوئی
Important information store نہیں ہوگی بلکہ وہ completely
anonymous ہوگی اگر آپ اس تمام condition
کو agree کرتی ہیں تو آپ اپنی Please verbally
جو ہے consent دیں آپ اسکی اجازت دیتی ہیں

جی. B

I اب آپ اپنے بارے میں بتائیں کہ آپ کیا Job
کرتی ہیں کیا کرتی ہیں

B میں staff ہوں یہاں پہ اور nursing کے
حوالے سے یہاں پہ duty کرتی ہوں
hashmanic hospital میں میرا نام [redacted] ہے

I آپ یہاں پر آپ staff nurse ہیں تو آپکی
یہاں پہ کیا کیا Job responsibilities ہوتی ہیں

B یہاں پر جو ہے نہ ہم doctors کے جو بھی
Patient کے حوالے سے medication یا انکی care
کے حوالے سے جو بھی ہوتا ہے نا وہ ہم follow کرتے ہیں

Date ________

I ٹھیک تو ایکا کچھ مطلب آپ جو ہے Patient کو Health کے حوالے سے یا غذائی معلومات ہوتی ہے اُن کو دیتی ہیں وہ آپ سے پوچھتے ہیں

B جی کھانے پینے کے حوالے سے جو کچھ Surgery کا Patient ہے؟ یا کوئی Bed Rest Patient ہے یا کوئی Anemia کے ساتھ ہے تو پھر ان کو جو بھی کھانے پینے کے حوالے سے جو Doctor نے advice کی ہوتی ہے [illegible] تو ہم تو وہی بتاتے ہیں لیکن اگر ہم کچھ دوبارہ بتانا ہو تو پھر وہ بھی بتاتے ہیں کہ اُن کیلئے کیا صحیح ہے For example کو Anemia کے ساتھ Patient ہے لیکن کہ blood اُن کا کم ہے یا blood بنتا نہیں ہے تو پھر ہم اس حوالے سے اُن کو guiding کرتے ہیں کہ آپ یہ والی چیزیں لیں Protein لیں Iron لیں تو یہی ساری چیزیں اُن کو advice کرتے ہیں

I ٹھیک ہے ٹھیک تو آپ کے خیال سے کیا ایکا لگتا ہے کیا Problems ہے یا مشکلات یا challenges ہیں جو کہ آپ کو دیکھنے پڑتے ہیں اپنی Job کے دوران جب آپ کسی کو advice کر رہی ہوتی ہیں کہ یہ کھائیں یا وہ نہ کھائیں یہ کریں وہ نہ کریں تو کیا مشکلات کیا پیش آتی ہیں آپ کو

B ہمم Mostly کھانے پینے کے حوالے سے تو کوئی مشکلات نہیں ہیں لیکن جو ہم بتاتے ہیں زیادہ تر وہ Patient Follow کرتے ہیں ہوتا ہے یا Available ہوتا ہے

I ٹھیک ہے لیکن آپ لوگ جو اُن کو بتاتی ہیں وہ آپ سے پوچھتے بھی ہیں اور Follow بھی کر رہے ہوتے ہیں

B ہاں کرتے ہیں

I ٹھیک ہے تو آپ نے کبھی اسطرح کبھی آتے ہیں کبھی اُن کو Counsel کیا اُن کو [illegible] دی جس طرح کہ یہ کھانا ہے یہ نہیں کھانا اور

Date

اُنھوں نے آ کر آپ سے پوچھا ہو اور آپ ان کو
بتا رہی ہوں چیزیں اور یہ سارے کام آپ
اپنے swallow کر رہی ہوتی ہیں ابھی پچھلے
تک ایسا تھا جی

ہوں جی (اس میں بہت سارے لوگ ایسے ہوتے Q
ہیں نا جو پڑھے لکھے نہیں ہوتے اُن کو ہم
بتا رہے ہوتے ہیں کہ basically یہ جو
نخرہ ہے کہ آپکے بچوں کو یہ نہیں لینا ہے ام
یہ لینا ہے تو اُس پہ وہ جو ہے نہ وہ swallow نہیں
کر پا رہے ہوتے کہ بچے ہیں ضد کر رہے ہیں یہ وجہ کہتے
ہیں) یہاں پر پچھلے دنوں ایک Patient تھا اُس
کو بہت زیادہ cough تھی syrup تھی یہ ساری
چیزیں تھیں تو ہم نے اُن کو پانی یا ٹھنڈا یہ پانی
وغیرہ چیزیں منع کی تھیں لیکن وہ نہیں اُس
پہ swallow کر رہے تھے ایسا بھی ہوتا ہے بعض
اوقات نہیں سمجھتے ہیں کہ ہمارے Patient کیلئے کیا
important ہے وہ بس یہی کہتے ہیں ہمارا Patient بچہ
جو require کر رہا ہے ہم نے اُس کو وہ require
کر لینا ہے۔ بعض اوقات ایسا بھی ہوتا ہے
لیکن جو پڑھے لکھے ہوتے ہیں وہ سمجھ جاتے ہیں تو وہ
swallow بھی کر لیتے ہیں

تو آپ کے خیال میں وجہ کیا ہوتی ہے کیوں نہیں
swallow کر رہے ہوتے ہیں کیوں نہیں مان رہے ہوتے

اب بعض اوقات بچے ہوتے ہیں نہ 2 یا 3 سال
کے بچے وہ رو رہے ہیں ضد کر رہے ہیں ماں باپ اُن کی
ضد میں آ جاتے ہیں تو چیزیں دے دیتے ہیں۔ اب
doctors ہیں وہ منع کر رہے ہیں کہ بازار کی چیزیں
یا ٹھنڈا پانی یا chips یا chocolate ہو گئے ہیں یہ ہو گئے
ہیں cold drink نہیں پینا بچے کو کھانسی ہو اور
چیزیں خراب ہو جائیں گی کوئی بچہ Medicine
کے ساتھ ہے تو ظاہر سی بات ہے اُن کو ٹھنڈا
چیزیں تو بالکل منع ہوتی ہیں مسئلہ ہے اکثر
ٹھنڈا چیزیں بالکل نہیں دینی ایسی چیزیں دینی ہیں

Date

اب بچہ کھاتا نہیں ہے ویسے ہی نخرے میں زبان میں بھی چھالے ہو جاتے ہیں تو وہ جو بھی demand کرتا ہے نہ ایک [illegible] وہ پورا کرتے ہیں وہ اُس کیلئے [illegible] harmful بھی ہو وہ تب بھی پورا کرتے ہیں کہ ہمارا بچہ ویسے ہی کچھ نہیں کھا رہا اور جو مانگ رہا ہے وہ آسکتا وہ بھی نہ دیں تو ایسے کیسے تو وہ پھر بعض اوقات نہیں سمجھتے ہمارے سمجھانے پر بھی I

ٹھیک ٹھیک، اور بعض ایسے بھی ہوتے ہیں جو مان بھی جاتے ہیں B

ہاں بہت سارے مان جاتے ہیں ظاہر سی بات ہے کوئی بڑا ہو گا اُن کو بتائیں گے کہ آپ کے Patient کیلئے یہ چیزیں صحیح نہیں ہیں تو وہ مان بھی جاتے ہیں سمجھ بھی جاتے ہیں کہ ہاں staff guidance کر رہا ہے پڑھا لکھا staff ہے وہ guidance کر رہا ہے تو یہ ہے کہ doctor نے جو بولا ہے اُس کو ہم follow کریں یہ ہمارے لیے صحیح تو وہ کرتے بھی ہیں follow

تو آپ کے خیال سے یہ Important ہے کہ ہم اُن کو غذائی چیزیں بتائیں اور اس پر عمل کریں I

بالکل Important ہے ہاں (غذا کا تو بہت ضروری ہے بتانا کیونکہ بہت ساری بیماریاں ایسی ہیں جو غذا سے related جو صحیح غذا نہیں لیتے ہیں تو بیماری کی طرف جاتے ہیں یہ آجکل جو calcium کی کمی ہے یا جو ہے نہ blood کی کمی ہوئی ہے بہت تیزی سے پہلے اتنا زیادہ نہیں تھا آجکل خون وغیرہ کی کمی کی اب دیکھا گیا ہے ہر دوسرے یا تیسرے بندے کو خون کی کمی ہے یہ نہیں ہے کہ ان کو blood چڑھانے کی ضرورت ہے لیکن خون کی کمی آ رہی ہے اور زیادہ تر females میں اور بچوں میں ہے) کیونکہ B

ہم یہاں کام کرتے ہیں نا جب سے اپنے Patients آتے
ہیں تو دیکھا ہوا ہے اتنی زیادہ awareness نہیں ہے
پڑھے لکھے لوگ نہیں ہیں مطلب ان سے اتنا
زیادہ نہیں knowledge ہے تو وہ لوگ جو
یہاں آتے ہیں زیادہ کم ہی ہیں اور یہ وہ مطلب
صحیح طریقے سے غذا نہیں لیتے دودھ ہو گیا
Protein ہو گیا انڈے ہو گئے یہ ساری چیزیں
Normally Routine میں انسان کو کھانا چاہئے
زیادہ نہیں لیکن اتنا تو کم انسان کھا ہی
لے اتنا کہ ہفتے میں 2 time meat
لے لے Mutton ہو گیا chicken ہو گیا لے لے
Beef لے لے یہ ان سب چیزوں کی بہت
ضرورت ہے اور یہ چیزیں لینی چاہئیں ہر
انسان کو کیونکہ اللہ تعالیٰ نے یہ ساری
نعمتیں اسی لیے بنائی ہیں نا کہ غذا most
important ہے کہ اگر آپ نے صحت مند
رہنا ہے تو غذا آپ نے اچھی استعمال کرنی
ہے ورنہ کیا ہو گا کچھ Time کے بعد آگے
muscles کی کمی آپ کے اندر blood کی کمی
یہ ساری چیزیں blood اور muscles calcium bones کا
کیلئے بہت زیادہ ضروری ہیں تو اگر ان
سب چیزوں کی کمی ہے تو Patients کوئی بھی
انسان میں ہوں آپ ہوں تو ہم لوگ اس
حساب سے workout نہیں کر پا رہے
جو ہمارے پورے ہوش ہیں workout کر پا رہیں
گے اور انکی awareness بنانا اس سے
بھی بہت اچھی بات ہے۔ اگر کوئی awareness
دے رہا اور اس چیز پر Research کر رہا ہے۔
بہت چیز اچھی وہ ہے۔ دوائی سے زیادہ غذا
معنی رکھتا ہے
تو ابھی بھی انسان ہیں کہ آپ جو ہیں اپنے گھروں
میں group میں یا کچھ community میں یا جہاں
بہت زیادہ لوگ اکٹھے ہوتے ہیں عورتیں ہوں

Date

رہے ہوں اُن سب کو ایک ساتھ کوئی بات بتا رہی ہوں کہ اچھا یہ کھانا ہے یہ نہیں کھانا یا پھر counseling کر رہی ہوں تو ایسی

زیادہ تر ہم اُن patients میں ایسا ہوا ہے group میں بھی advice کیا لیکن آپ patients یا family بتائی ہوں کہ بھئی یہ چیزیں زیادہ ضروری ہیں آپ کیلئے father ہیں یا بھائی ہیں یا بہن ہیں کسی کا operate ہوا ہے اُن کو بتائی ہوں کہ یہ چیزیں آپ کیلئے زیادہ ضروری ہیں یہ healthful ہیں یہ چیزیں نہیں لو یا کم لو ہے نا تو اس حساب سے الگ بھی group میں نہیں بتایا کہ یہ چیزیں آپ کو لینی چاہئیں یا نہیں تو بتانا چاہیئے As a medical professional جو انسان کو لوگوں کو بتانا چاہیئے At least اپنی family اپنے اردگرد جو لوگ ہیں اچھا ہے جو کر رہا ہے نہ جو بھی nutrition ہے اگر وہ یہ چیزیں عام کر دے تو بہت اچھا اس سے آنے والی زندگی میں تو

ٹھیک ہے ٹھیک تو آپ کیا رائے کیا Suggestions دینا چاہیں گی کہ ہم اس کو improve کرنے کیلئے کہ لوگوں کے اندر آگہی بس counseling کر سکیں یا ایسی nutrition کے حوالے سے اچھی ہو یا ایسی صحت اچھی ہو

اس کیلئے میں یہ نہیں کہتی کہ پڑھا لکھا انسان ہونا چاہیئے اس کیلئے میں یہ کہتی ہوں کہ کوئی ایک سمجھدار انسان ہو جو اگر جیسے بچے ہیں تو ان کو نہیں آتی سمجھ تو ہم ان کو جو لوگ ہیں وہ نہیں سمجھا سکتیں جلدی لیکن کوئی ایک بڑا جیسے کوئی father ہے mother ہے یا [illegible] ہے ان کو ہم سمجھا سکتیں ہیں کہ یہ آپ کیلئے اچھا ہے مطلب اگر آپ نے بہت زیادہ نہیں پڑھا تو آپ نے روزانہ دودھ کا استعمال

کرنا ہے انڈے کا استعمال کرنا ہے گوشت کا استعمال کرنا
روز نہیں گوشت کا تو ایسا ہوتا ہے کہ جو ہے نا ایک
دن چھوڑ کے بھی آپ نے استعمال کر لیا ہے تو ایک
اور وہ چیزیں پوری ہو گئی ہیں [illegible]
سبزیوں کا استعمال کرنا تاکہ اسکو کوئی قبض نہ ہو
یا کوئی اس طریقے کی چیزیں نہ ہوں چیزیں
نہیں کوئی بھی ایک constipation ایسی بیماری
ہے جو فوراً سے نہیں آپ کو harm کرتی لیکن
بھی آگے جا کر بعد اسکو harm کرنے لگتی ہے انکی
انتڑیاں خراب ہو سکتی ہیں کوئی بھی ایک
constipation کا patient ہے اور اس نے بہت
دنوں تک نہیں pass stool کیا تو اسکی
انتڑیاں خراب ہو سکتی ہیں اور جہاں ہم نے
ایسے سیریس کیسز دیکھیں ہیں جو constipation
کے ساتھ تھا اور اس نے اپنے اوپر care
نہیں کیا اسے نہیں سمجھ پایا اس وقت یا اس نے
[illegible] کو avoid کیا اس وقت تک اسکا abdomen
نہیں ہوا یا سخت ہو گئی اس کی انتڑیاں ہیں
[illegible] ہو گئیں اور پھر کیا ہوا کہ operation
ہوا اور انتڑیوں کو cut کر کے نکالا گیا
مطلب کہ ایک بہت بڑا ایک procedure
ہو گیا اگر وہ پہلے time ان چیزوں کا
استعمال کرتا اچھی fresh سبزیاں لیتا Routine
میں دالیں رکھتا Routine میں جو ہے نا اپنا گوشت
رکھتا انڈے رکھتا دودھ رکھتا مطلب یہ
ساری چیزیں اگر یہ ساری چیزیں وہ normally
کھاتے پیتے رہتا تو وہ بھی صحت مند رہتا اتنی
بڑی بیماری اس کو نہیں لگتی مطلب کہ
حالانکہ دیکھا جائے تو constipation ایک چھوٹی
سی بیماری ہے پر شریف بندے کو constipation
ہوتی ہے وہ ہوتی اسی لیے ہے کہ اسکی
غذا اچھی نہیں ہے junk food کھاتے ہیں بازار
کے ہیں اگر یہ bread یہ ساری چیزیں لیتے ہیں

[illegible] سے تکلیف ہوتا problem ہو رہی ہے

I یعنی کہ آپ کہہ رہی ہیں کہ خالی اپنی صحت کا
خیال ہی رکھ لیتے تو اتنی ساری ۔۔

B بیماریاں نہیں ہوتیں کچھ لوگ اب یہ نہیں
نہیں کہوں گی کچھ چیزیں اللہ کی طرف سے بھی
ہوتی ہیں ضروری نہیں بہت سارے لوگ
ایسے ہوتے ہیں جو اپنا بہت care کرتے ہیں
پھر بھی وہ بیمار پڑ جاتے ہیں تو وہ تو
پھر اللہ کی طرف سے بھی ہوتا ہے لیکن غذا
کا میں کہتی ہوں (غذا کے حوالے سے ہر بندہ اپنی
بہتری کرنی شروع کر دے نا تو ایک اچھا
Improvement ہو سکتا ہے اس کیلئے بھی اور
اور چیزوں کیلئے بھی تو میں کہتی ہوں کوئی
ایسا session ہونا چاہئے جو غذا کے
حوالے سے جو ہوتا ہے information دے
آگے guide کرے جن لوگوں کو knowledge نہیں
ہے یا جن کو knowledge ہے ان کو بعض اوقات
sessions کی ضرورت ہوتی ہے reminder کی
ضرورت ہوتی ہے) تو ان کو پتہ چلتا ہے کہ
ہاں ہمیں ان چیزوں کی ضرورت ہے need
ہے

I یعنی کہ آپ یہ کہنا چاہ رہی ہیں کہ کوئی
ایسا person ہونا چاہئے کہ جس کے پاس
پوری complete information ہو

B Information ہو

I اور وہ سب کو guide بھی کرے

B guide کرے

I اور وہ آگے سے لوگوں کو guide کریں
تاکہ یہ ساری چیزیں پھیلیں

B ہاں

I اور ہر انسان جو ہے نا

B صحت مند رہے

I صحت مند ہو جائے

Date

ٹھیک ہے ٹھیک تو اب آپ جو بھی اپنا رائے I
کیا دینا چاہ رہی ہیں آپ آخر میں کوئی
بات کرنا چاہیں گیں آپ کوئی بھی کسی
چیز کے بارے

بس میں یہی کہنا چاہوں گی کہ جو بھی انسان B
ہے ہر انسان میں آپ نے ہر ایک proper
اینڈ لیٹے وہ چیز ہی نہیں کر پاتا جو میں
کہہ رہی ہوں نہ ہی بعض انسان کام
کے ساتھ میں بچوں کے اسمیں گھر والوں
کے اسمیں اپنی بہت ساری چیزیں چھوڑ
دیتے ہیں ٹھیک کر دیتے ہیں مگر میں
کہتی ہوں کہ ہم لوگ پہلے لیکن آپ لیں ضرور
کیونکہ صحت بہت ضروری ہے آگے اپنی
اچھی حیات گزارنے کیلئے کیونکہ اگر صحت
ہوگی تو ہی ہم دوسروں کا بھی خیال رکھ
پائیں گیں یقینی کہ اپنی family کا۔

تو آپ کو Husband Support وغیرہ دینا I
چاہیں گیں کہ کس طریقے سے support کر سکتے
ہیں ہم

ہاں کس طریقے سے support کر سکتے ہیں B
اسمیں Husband کا بہت سب سے زیادہ
کردار ہے اگر کوئی انسان اپنی صحت
کا خیال رکھ سکتا ہے تو وہ بھی دوسروں
کو بھی balance کر سکتا ہے کہ ہاں ٹھیک ہے چیزیں
لیتا ہوں تو میں ٹھیک صحت مند ہوں تو وہ
ہم آگے بھی balance کرے سب کو

Ok Thank you so much , Thank you I
بہت شکریہ

Thank you B
