# Supplementary material for: Qualitative exploration of comprehension and experiences of healthcare professionals regarding nutrition care in Karachi, Pakistan
Source: PLOS Glob Public Health. 2025 Dec 30;5(12):e0005483. doi: 10.1371/journal.pgph.0005483 (PMC12753000; doi:10.1371/journal.pgph.0005483)
Supplement: S5 File — (ZIP) [file pgph.0005483.s005.zip › Nurse Male -006.pdf]

Date [redacted] Nurse Male-006

I اسلام علیکم آپکا نام

A [redacted]

I اچھا ہم لوگ Research work کر رہے ہیں Ziauddin University میں
کے students ہیں PhD کر رہے ہیں Research work جو ہے
ہمارا اس میں ہم nurses & doctors سے different interviews
لے رہے ہیں ان سے ان کے خیال پوچھ رہے ہیں suggestions
& opinions پوچھ رہے ہیں Nutrition care یا غذائی صحت
کے حوالے سے تو اس سلسلے میں جو ہے نا ہم آپ سے
تھوڑے بہت سوالات کریں گے اگر آپ کی مرضی ہے کہ
اگر آپ کسی سوال کا جواب دینا چاہیں تو اچھی بات
ہے اور اگر نہیں تو اس کا خیر Interview پر کوئی اثر نہیں
پڑے گا۔ تو جو ساری جو معلومات آپ ہمیں دیں
گے ہیں Research Purposes کیلئے استعمال ہونگی اور اس
میں جو آپ کا نام وغیرہ ہے یا جو بھی چیز ہوگی وہ
Anonymous رہے گی ٹھیک ہے تو اگر آپ Agree
کرتے ہیں تو آپ اپنا نام بتادیں اور یہاں پر (consent form)
sign کر دیں۔ آپنے اپنا نام کیا بتایا تھا

A [redacted]

I [redacted] ٹھیک ہے اچھا اپنے بارے میں کچھ
بتائیں کیا ہیں آپ

A میرا نام [redacted] ہے اور میں یہاں پر As a technician
کام کر رہا ہوں ICU Technician میں JSIC department
جس میں میں duty کرتا ہوں

I کتنے عرصے سے ہیں آپ

A کوئی چار سال

I ٹھیک ہے تو آپ کا اگر ہم نام لیتے ہیں Nutrition Care
کا یا غذائی صحت کا تو کیا آپکے دماغ میں چیز آتی ہے
کیا آپکو سمجھ آتی ہے کہ کیا ہے کیا چیز یہ؟

A Healthy food وغیرہ جس طرح Fruits
ہوتے ہیں Nutrition جو ہمارے ہوتے ہیں اس
کے علاوہ Plan کرنا اچھی ہونی چاہیئے
جس طرح Fish ہوگئی nutrition ہوتے ہیں اس میں
بھی جتنے بھی meat میں nutrition count میں

Date ______

ہوتے ہیں vegetables بھی nutritious ہیں جو ہوتے
ہیں تو اس طرح کی چیزیں کھانے سے آپکی صحت
اچھی رہتی ہے۔

اچھا کبھی جیسے آپ کام کر رہے ہیں ICU میں T
آپ نے کہا ICU میں آپکا زیادہ Time ہوتا ہے
ICU میں تو بہت مطلب Patient جو ہوتا ہے بہت
Critical ہوتا ہے تو اس کے اندر کیا آپ لوگوں
کا ہوتا ہے کس طریقے سے اپنے انکو خوراک دینی
ہے کیا کرنا ہے کس طرح Treatment کرنی ہے

Basically جو ICU کے Patient ہوتا ہے وہ A
NG Feed پہ ہوتا ہے تو Patient جو ہوتی
ہے اکثر وہ Glucerna ہے یا Ensure ہے یا
ہم Basically اس طرح کی Diabetic milk
دیتے ہیں جو Milk Powder کی شکل میں
Patient کو دیا جاتا ہے ICU میں بہت
کم Rare ہوتا ہے کہ Patient کوئی
Oral diet لے رہا ہو اور اگر Patient on ہے
تو Basically وہی آتا ہے جس طرح کھا رہا
ہوں اس جیسی چیزیں Fruit بھی آتے ہیں Patient
میں اس کے علاوہ chicken بھی ہوتی ہے Soup
ہوتا ہے جتنی بھی اچھی Patient آتی ہے
Patient کی اس کے علاوہ زیادہ تر جو ICU
کا Patient جو ہوتا ہے وہ Ensure پہ یا اس
milk پہ ہی ہوتا ہے

Ensure Hmm وہ تیمارداران کے دیتے ہیں۔ T

Ensure یا Glucerna A

تو مطلب کبھی اس طرح ہوتا ہے کہ آپ سے جو T
اپنی رائے مانگ رہے ہوں کہ آپ ہمیں بتائیں
Suggest کریں کیا کس طرح ہم اپنے آپ کو مطلب
بہتر کریں اچھی خوراک لیتے۔

نہیں (lightly smiling) Patient ہم سے تو نہیں A
پوچھتے ہیں Doctor جو decide کرتے ہیں
کہ Patient کو ابھی فلحال اگر NG پہ ہے

Date

ور اور oral intake نہیں ہے ہو سکا تو کیا اسکیلئے
مسئلہ ہو گا پر اس کے علاوہ ہمارے پاس
Hospital میں dietitian ہیں جو daily
morning میں round کرتے ہیں اور دیکھتے
ہیں کہ patient جو NG پہ ہے تو اس کیلئے کونسی
کیسی feed کتنی RD چلنی چاہئیے 150 ، 3 RD
ensure یا glucerna جو بھی اس کیلئے better ہے
اگر تو وہ digest کر رہا ہے تو feed ٹھیک ہے ورنہ اگر نہیں
digest کر رہا tolerate کر رہا تو feed hold کی جاتی ہے
feed

Hmm تو یہ Doctors وغیرہ ہی decide کر رہے ہوتے T
ہیں اور dietitian بھی help کر رہے ہوتے ہیں
جی جی doctors A
ٹھیک ہے ٹھیک ہے تو کبھی اسطرح ہوا کہ جس T
طرح ہوتا نا کہ بہت سارے لوگوں میں یا community میں
میں کبھی اسطرح کا موقع ملا ہو کہ آپ نے کبھی
ان کو کبھی کسی بھی حوالے سے guide کیا ہو
family members میں یا relatives ہیں تو اس طرح A
کوئی پوچھتا ہے تو کہ مجھے weakness ہوئی ہے
General weakness ہے تو کیا چیز کیا Supplement
میں Apply کرتے basically تو زیادہ تر
Avoid لوگ کرتے ہیں Medicine کو Avoid کرتے
ہیں جسطرح Capsule ہو گئے Surbex Z ہو گئی
Vitamins [illegible] ہو گئے ان کو Avoid کرتے ہوئے
زیادہ کوشش یہ ہوتی ہے میں خود بھی کوشش
یہی کرتا ہوں کہ زیادہ تر meat کا استعمال کروں
کیونکہ اس میں nutrition زیادہ ہوتا ہے Meat
جسطرح fruits ہیں Fruits کھانے چاہئیں
آپکو ان سے vitamins ملتے ہیں تو basically
اگر medicine اگر بہت زیادہ expensive ہو جاتی ہے نہیں
آپ کو اثر ہو یا کسی چیز کا تو ہم ہم medicine
اور چیزوں کو apply کر سکتے ہیں
Hmm ٹھیک ہے اگر کوئی جس طرح آپ کے کہنے کا T

Date

مقصد ہے کہ ایسی کوئی بھی اپکا Opinion ویسے jam [illegible]
بے رہا ہوتا ہے تو ہم اب ان کو balance کرتے ہیں
یہ چیز کھا لیں یا یہ دوائی اچھی ہے یا chicken کی A
بنا لیں اس طرح کی چیزوں کو balance کیا جاتا ہے
باقی کوئی ایسا Issue کوئی اس طرح کبھی مطلب موقع I
نہیں ملا کہ سب کے سامنے کوئی nurse وغیرہ
یا کوئی ایسی چیز بتانے کا یا کوئی ایسا کام کرنے کا
ایسا کچھ نہیں ہوا آپ کو اب تک
نہیں A
ٹھیک ہے تو آپ جس طرح کام کر رہے ہوتے ہیں اپنے I
ICU میں سب چیزوں میں جیسے ان Patients کی بھی
diet سب چیزوں کو آپ کو کیا مشکلات
پیش آ رہی ہوتی ہیں اس وقت کہ ان کو دے
رہے Food بھی دے رہے ہیں چیزیں بھی دے رہے
ہیں تو کیا مشکلات ہوتی ہیں آپ لوگوں کو
ہمیں تو کوئی مشکل نہیں ہوتی Patient کو اکثر A
ہوتا ہے جس طرح میں نے بتایا آپ کو NG
Feed پہ ہوتا ہے تو ظاہر سی بات ہے کم لوگ
کم Patients Feed کو Suffer کرتے ہیں مطلب
conscious ہوتے ہیں ٹھیک ہو کے جاتے ہیں diet
اچھی رہی ہے اکثر Patients ہوتے ہیں کم Oral
جو ہوتے ہیں وہ بیمار ہے کھا نہیں پا رہے ہوتے اور
Feed آتی ہے ایسا بھی ہوتا ہے کسی کو
Pressure لگا ہوا ہے by Pipe mask ہوتا ہے اچھا
خاص کر Feed کے اوپر ان کے لیے اگر وہ لگے
[illegible] آتی ہے تو وہ mask اتار کے کھانا
اس کے بعد پھر لگانا پھر کھانا اس طرح بہت
مشکل ہوتی ہے ان Patients کیلئے جن کو
SOB کی Complaint ہوتی ہے۔ وہ Feed نہیں
کھا پائے صحیح طریقے سے اور اس کے علاوہ بس
یہی ہوتا ہے Patients والے Peptic ہوتے ہیں
Peptic والے Patients کا ویسے same ہوتا ہے
اس کیلئے تو کوئی Issue نہیں ہے

I: تو آپ کے خیال سے کون زیادہ بہتر طور پر Normal
جو ہے نا یہ دے رہے بتانا بیشک غذائی صحت کے حوالے سے
کون زیادہ اچھا Guide کر سکتا ہے بتا سکتا ہے

A: Basically جو Normal ہوتے ہیں ہمارے یہاں پہ
وہی زیادہ اچھا Guide کرتے ہیں کیونکہ ان کا
پورا ایک Plan ہوتا ہے Paper ہوتا ہے
جس پر سارا کی نیوٹرین لکھی ہوتی ہیں اور Students
بھی انہوں نے بھی پڑھی ہوتی ہے اسی چیز میں تو
Basically ان کیلئے یہی سب سے زیادہ Important
ہوتا ہے یا ان کا ہی Main character ہوتا ہے
ایک Patient کے لحاظ سے کہ وہ اس کی
Health کو Perfect کر سکیں وہ اچھا

I: ٹھیک ٹھیک تو ویسے تو ہم چلو ابھی
تو ہم نے اپنے Hospital کے حساب سے بات کی
ویسے Generally اگر ہم بات کریں تو آپ کے خیال
سے کس طریقے سے ہم بہتر بنا سکتے ہیں عام عادات؟
سب بھی صحت جو ہے غذائی صحت جو ہے وہ
کس طریقے سے اچھی ہو سکتی ہے

A: ہم جس طرح ہم جاتے ہیں اکثر ہم جاتے ہیں doctor
کسی بھی Local Hospital میں یا کسی clinic پہ Personal
کے ساتھ جاتے ہیں کہ Doctor ہمیں کچھ Guide کرتے
ہیں کہ یار یہ چیز نہیں کھانی ہے گا یہ کھانی ہے گا
ایسا سینہ خراب ہے یہ چیز ٹھنڈی نہیں پینی ہے گا
یہ والے Fruits نہیں کھانے اکثر بولتے ہیں
آلو سے avoid کریں Rice سے avoid کریں
تو کم ہوتا ہے تو doctors زیادہ بہتر Guide
کرتا ہے Patient کو اور اس کے علاوہ جو ہے
ہمیں خود بھی تھوڑا Knowledge ہوتا ہے سب کو
ہی ہوتا ہے کہ یہ چیز ہمارے لیے اچھی ہے
یہ کھانی چاہیے ہیں سے ہمیں یہ چیز حاصل ہوگی
جسمانی طور پہ اس کے یہ فائدے ہیں تو Basically
ہمیں خود بھی Idea ہوتا ہے لیکن کچھ چیزیں
doctor ہمیں guide کرتے ہیں کہ آپ کیلئے

Date ________

ابھی یہ چیز صحیح نہیں ہے یہ نہیں کہا ئیں ہمیں جس طرح
اگر کوئی چھوٹے سے Level کے لحاظ سے جاب
ہو گیا تو اس کے لحاظ سے چیزیں ہیں جو Level
کی جاتی ہیں یہ نہیں کہائیں یہ آپ کے Field
میں میں ہوتا ہے جیسے تلی ہوئی چیزیں منع کر دی
جاتی ہے تلی ہوئی چیزیں نہ کھائیں جگر اگر
بگڑا ہوا ہے تو اس طرح doctors زیادہ اچھا
ہمیں Suggest کرتا ہے

آپ اپنی بات کر رہے ہیں یا یہ لوگوں کو I
نہیں لوگوں کو Suggest کرنا A
لوگوں کو بہت اچھا Suggest کر سکتا ہے تو I
یہ کوئی تو مطلب doctors کے پاس نہیں آ رہا
ہوتا تھا یا پھر جو مریض ہوتے ہیں صرف وہی جا
رہے ہوتے ہیں تو Awareness لوگوں کو کس طرح سے
سے جو Public ہے بالکل ہے Awareness [illegible] ہے جو لوگوں
میں پیدا ہوئی ہے اُن کو کس طرح سے آپ
کم سکتے ہیں

اس لحاظ سے اگر اب بات کریں تو آپ A
کوشش ہے کہ اب TV پہ Ads آتے ہیں
جو Health کے Ads آتے ہیں اس پہ
لکھا ہوتا ہے یہ Bones سے طاقت ملتی ہے اس
سے مضبوط ہوتی ہیں لوگ دیکھتے ہیں
یہ ایک اچھا Channel ہے Awareness کا جو
Health کے [illegible] Banners بھی اکثر کافی
کبھی کبھی لگے ہوتے ہیں یار یہ چیزیں نہیں آپ
یہ کھائیں گے اس سے آپ کا Cholesterol بڑھے
اس کے اندر بھی یہ چیزیں ہوتی ہیں کافی ہوتی
آتی ہیں باقاعدہ اچھی طرح جتنی بھی چیزیں
ہیں مطلب Products کے لحاظ سے یا کسی بھی
چیز کے لحاظ سے دیکھتے ہیں Banners کے لحاظ
سے Channels کے لحاظ سے کے Computers کے
لحاظ سے Net پہ اتنا کچھ ہے دیکھنے کیلئے
لوگ Net سے بھی Search کر کے دیکھتے ہیں

Date ________

تو میری نظر میں تو یہ ایک source ہے جو بہت
کر guide کر سکتا ہے population کو guide
کر سکتا ہے
یعنی آپ I
جیسے doctors کے پاس کوئی نہیں جا سکتا ہے جیسے؟ A
میں تو چلیں nursing میں ہوں میں تو یہ office
work کرتا ہے تو وہ doctor کے پاس نہیں
جا رہا وہ ٹھیک سے physically ٹھیک ہے
تو ہم بھی اگر اسے feel ہوتا ہے کہ یار مجھے
feel یہ دیکھنا ہے کہ یار یہ چیز کھاتا ہوں
مجھے اچھی لگتی ہے اس کے اب کیا نقصانات ہیں
تو وہ ایک زیادہ بہتر بتا سکتا ہے
یعنی کہ آپ کے کہنے کا مقصد ہے کہ یہ جو I
ہمارے پاس sources ہیں TV
ہو گیا Computers ہو گئے Browser
ہو گیا social media ہو گیا اس کے اندر
یہ کہ انسان یہاں سے search کر کے اور
یہاں سے لوگ اپنی معلومات وغیرہ
ہاں اگر کوئی guide کرنے والا نہیں ہے تو A
پھر تو یہ چیزیں ہیں یہاں سے ہی تھوڑا سا
گمراہ کر سکتی ہیں انسان کو)
ہاں تو وہاں یہ بھی دیکھا جاتا ہے بہت ساری I
چیزیں غلط غلط بھی پھیلی ہوتی ہیں
بالکل A
تو ہمیں اس کا کیا کرنا چاہیئے کہ ہم ایسا کچھ I
سوچا جائے جو اس کو بھی دیکھ رہا ہو
تو ہم اس والی بات پر آتے ہیں تو ایک example A
ہے پہلے ہمارے پاس جو options ہوتے تھے
فرض کریں ہوتے تھے تو جو یہ cream ہوتی ہے
وہ گھروں میں بناتی تھیں
Hmm I
ٹھیک ہے اب نہیں بناتی اب لوگ purchase A
کر لیتے ہیں اب وہ وہ cream نہیں ہوتی

اس کے کم side effects ہوتے ہیں وہ اس طرح
نہیں Best ہوتی ہوتی جس طرح گھر پر ہوتی
ہوتی تھی اس طرح کی واقعی کوئی ایک دو
چیزیں ہوتی ہیں جو ہمیں Net پر دکھتی ہیں
یا ہمیں Advertisement سے ہم دیکھتے ہیں
ہم دیکھ کر لیتے ہیں کہ ہم یہاں یار یہ چیز اچھی
ہے cream [illegible] بنائی [illegible]
نہیں کرنی پڑتی تو یہ Influence کرنے والا کوئی
نہیں ہے کہ ہاں یہ چیز اس طرح صحیح نہیں
ہے کہ آپ اس طرح اس کو Implement کریں
تو بس
تو کون ہونا چاہیے آپ کے خیال سے کس سے I
ہم کس طرح ہم کر سکتے ہیں صحیح والی
Awareness کس طرح پھیلا سکتے ہیں
لوگ ایک دوسرے کو بتائیں مطلب A
کہ میں ایک دوسرے person کو بتاؤں
وہ آگے کسی اور کو بتائے یار یہ چیز
نہ کھائیں اس چیز سے Problem کریں
یہ چیز کھائیں یہ چیز زیادہ اچھی ہے
جس طرح ہم اگر آپ مجھے کچھ بتائیں گی
آپ کو پتہ ہے Knowledge ہے تو آپ
مجھے بتائیں گی تو میں آگے لوگوں کو
بتاؤں گا کہ آپ یہ چیز کھائیں یہ نہ کھائیں
یہ صحیح نہیں ہے اس طرح بہتر چلے گا
یعنی کہ لوگ ایک دوسرے سے باتیں کر کے I
جو ہے نہ وہ چیزوں کو پھیلائیں آگے
پھیلائیں A
ٹھیک ہے ٹھیک ہے doctors جو ہوتے I
ہیں ہمارے پاس hospital میں وہ بہت
busy چل رہے ہوتے ہیں ان کے ایک ایک
کے پاس کافی load ہوتا ہے Patients
کا تو آپ کو ایسا لگتا ہے کہ وہ صحیح معلومات
کو Patient جاننا چاہتے ہیں وہ اس کو سب بتا[illegible]

چل رہا ہوتا ہے یا Time کی کمی ہو رہی ہے
یا کوئی اور مسائل ہو رہے ہیں تو کیا کیا
جائے اس سلسلے میں

A تو پہلی بات میں میں کہوں گا آپ
جو یہ بات کہہ رہے ہیں میں نے جو یہ note
کیا اور different hospitals میں بھی کام کیا اور یہاں
پر بھی چار سال کام کیا تو دیکھا ہمارے پاس
جو ہیں doctors ہیں discuss کرتے ہیں اچھا
discuss کرتے ہیں patient کو attendant کو
اگر patient conscious ہے اور اس کو اتنی
سمجھ ہے کہ وہ سن رہا ہے اور بات کو سمجھ رہا ہے
تو تو وہ بات پر عمل کرتا ہے ورنہ اگر patient
Psychosis میں ہے یا نہیں سمجھ رہا بات کو
سمجھ نہیں رہا تو attendant کو بتایا جاتا ہے کہ
بھائی آپ کے patient کے ساتھ یہ یہ چیزیں
ہوئی ہیں آپ کو briefly discuss کیا جاتا
ہے تاکہ کل کو کوئی misunderstanding نہ ہوں
تو یہاں پر میں نے note کیا کہ doctors
اچھا discuss کرتے ہیں بتاتے ہیں safety
کرتے ہیں تاکہ کوئی issue بعد میں نہ ہوں
وہ اگلی بات ہے کچھ نہ کچھ کوئی نہ کوئی بات
ہو جاتی ہے لیکن ہم نے جو دیکھا ابھی تک
کیونکہ یہ آپ attendant interaction سے بھی بہت
ہوتا ہے اور patient سے بھی بہت ہوتا ہے اور
doctors سے بھی بہت ہوتا ہے تینوں سے
بہت ہوتا ہے تو اسلئے میں نے جو note کیا
کافی چیزیں بتائی جاتی ہیں پہلے لیکن کہیں
یہ الگ بات کہ misunderstanding ہو جاتی ہے لیکن
اتنا بڑا issue ان باتوں پہ نہیں ہوتا لیکن
doctors پورا اپنی طرف سے discuss کرتے ہیں
سمجھاتے ہیں بتاتے ہیں تاکہ کبھی بھی mishape
نہ ہوں

T ٹھیک اس کے علاوہ کچھ add کرنا چاہیں گے

Date ______

کس طرح سے nutrition care ہم بہتر بنا سکتے ہیں معاشرے
میں لوگوں کی individual basis پہ یا
[illegible] base پہ

A وہی بات کہ میں پھر دوبارہ کہوں گا جس طرح میں
نے بتایا کہ آپ مجھے کچھ guide کریں گے کہ یہ
چیز اچھی ہے یہ کریں آپ تو میں آگے بتاؤں
گا لوگوں کو تاکہ وہ بھی اس چیز کو implement
کر سکیں تو اسی طرح چیزوں کو بڑھایا جا سکتا
ہے اگر یہ کہا جائے کہ through social media کے
کرنا چاہیں تو وہاں سے بھی کر سکتے ہیں یا
awareness کو بڑھانا چاہیں یا جو بھی آپکے [illegible] میں
nutrition ہے اس لحاظ سے بھی
health
کو بہتر کیا جا سکتا ہے

Q (۲) بات ہے social media پہ سب سے بہترین
کون ہے بتانے والے کون ہو سکتا ہے Person
ہیں یا وہ کوئی ایسا بندہ ہو کم جس
doctor
کا nutrition background کا ہو کون ہونا
چاہئے

A جن کا background nutrition ہے یا وہ
Doctors جن کی [illegible] دے سکتا ہے
زیادہ اچھا guide
کوشش بھی ہوتی ہے ان کی
basically
medicine کی طرف زیادہ توجہ ہوتی ہے
ٹھیک

Q صحیح

A کیونکہ وہ بھی کہیں گے اگر blood کی کمی ہے
تو آپ supplements کر لیں یا Transfusion
نہیں کروا رہے blood نہیں لگا رہے تو چلیں
آپ کو injection لگا دیتے ہیں blood کی
کمی کے لیے nutrition basically ہے تو
کی طرف نہیں جا رہے ہوتے وہ medicine کی طرف
جا رہے ہوتے ہیں [illegible] HB کا patient کا اسکا
improve ہو ٹھیک ہے تو اس لحاظ سے میں
یہ کہوں گا کہ nutrition والا بندہ زیادہ اچھا

Date ______

growth کم ہو سکتا ہے کہ ہاں جی آپ یہ feel کر لیں
گے یہ Tictoc لیں گے تو آپکا download بڑھے
گا کم نہیں ہو گا

I صحیح اور اس میں کچھ اور Add کرنا چاہیں گے

A نہیں بس

I Thank you so much بہت اچھی معلومات دیں
آپ کے time کا بہت شکریہ

A Thank you

I شکریہ

A اللہ حافظ

I اللہ حافظ
