# Supplementary material for: Qualitative exploration of comprehension and experiences of healthcare professionals regarding nutrition care in Karachi, Pakistan
Source: PLOS Glob Public Health. 2025 Dec 30;5(12):e0005483. doi: 10.1371/journal.pgph.0005483 (PMC12753000; doi:10.1371/journal.pgph.0005483)
Supplement: S5 File — (ZIP) [file pgph.0005483.s005.zip › Nurse Male -008.pdf]

Date [redacted] Nurse Male-008

I: ہم Research کر رہے ہیں جس کے اندر ہم Doctors & Nurses
کے Interviews لے رہے ہیں اُن سے کچھ سوالات کر رہے
ہیں کہ اُن کی رائے کیا ہے Nutrition کے بارے میں یا غذائی
صحت کے بارے میں اور آپ کا Concept تو اس لحاظ سے ہم
آپ سے کچھ سوالات کریں گے تو جس کا چاہیں آپ
جواب دے سکتے ہیں جس کا چاہیں آپ نہ دیں اس
کا کوئی اثر نہیں ہوگا ہماری اس Research کے اندر
اور یہ جو Research ہوگی اس کی معلومات ہم
اپنی Publication میں استعمال کریں گے لیکن آپ کا جو
نام ہے وہ نہیں ہوگا Anonymous رہے گا اپکی جو
معلومات ہوں گی وہ Research Paper کیلئے استعمال
ہوں گی تو اگر آپ Agree کرتے ہیں ان ساری
چیزوں کا تو Kindly مجھے یہ Consent دے دیں
اور یہاں پر Sign کر دیں اپنے Thank you so much
اچھا جی سب سے پہلے تو آپ اپنے بارے میں بتائیں
تھوڑا سا ہمیں

A: میرا نام [redacted] ہے RN کیا ہوا ہے پھر میں نے Specialist
کی ہے میں نے Paeds میں Cardiac میں پر Complete نہیں
ہوئی پھر میں نے Post RN میں [redacted] سے کر رہا
ہوں جس میں ICU میں کر رہا ہوں پھر اب میں Gastro میں
Joining دی ہے

I: تو آپ کے خیال سے کیا ہے غذائی صحت ایک دماغ
میں کیا چیز آتی ہے پہلے کہ یہاں پہ ہے غذائی صحت یا
Nutrition Care

A: جیسے Nutrition کے حساب سے ہم دیکھتے ہیں Patient
کو جیسے دیکھا جاتا ہے کہ ان کا اپنی Related Field سے
تا کہ (Nutrition ہو جاتی ہے تو ان کو weakness
Proper نہیں مل رہی Protein وغیرہ یا High Protein
اس طرح سے تو کیسے Patient صحت یاب ہو گا
اٹھ کے بیٹھے گا یا اس طرح کی چیزیں تو اس لحاظ
سے ہمیں High Protein دینی ہوتی ہے جیسے
Feed کرتے ہیں اگر NG پر ہے تو Feed دی
جاتی ہے تو اگر Oral پر ہے تو اس حساب سے

Date

جو High Protein ہوتی ہے وہ Diet Accordingly وہ Decide
کرتے ہیں کہ Regular میں دینی ہے
لیے یا کیا ہے

I صحیح Doctors decide کرتے ہیں اب یہ کیسے ہے
یہی High Protein Diet دینی چاہیے اب یہ کہ

A Hmm..

I اچھا ایسا کبھی واسطہ پڑا کبھی Nutrition Care
یا غذائی صحت کے حوالے سے

A نہیں کہ ایسا نہیں ہے یہاں کا جو System
ہے وہ تو Doctor decide کرتے ہیں یا تو
وہ آتے ہیں Dietitian تو وہ
اگر دیکھتے ہیں تو پھر وہ Diet change کر دیتے
ہیں

I صحیح ایسا کبھی one to one Patient کے حوالے
سے کبھی

A Patient کے حوالے سے ہمارا زیادہ تر رشتہ ہے
اگر ہم Care, Medication سے Related ان
ساری چیزوں سے ہوتا ہے زیادہ تر یہ
ہوتا ہے

I غذا کے حوالے سے

A غذا کے حوالے سے نہیں ہو

I Patient کچھ پوچھتے نہیں ہے آپ کو

A جیسے کہ مطلب کیا

I کبھی مطلب ہم کیا کھا سکتے ہیں

A نہیں وہ تو جب Discharge ہوتے ہیں
تب Patient کی Condition کے حساب سے ہم
بتاتے ہیں اگر یہ NG Patient ہے یہ چار بار
لینے تو ہم انکو بتا دیتے ہیں ایک بار یہ
جو Resource ہے یا Glucerna ہے Milk
اس طرح سے جو بھی ہے آپ نے وہ 150
میں 150 ml کا گلاس ہوتا ہے اس میں 3 Tbsp
ڈال کے حل کر کے دینا ہے اس طرح سے اگر
Regular Diet پہ ہوتے ہیں جیسے ان کا Stomach
Disturb ہوتا ہے تو کہہ دیتے ہیں۔

Date ____

زیادہ Roasted والی Spicy چیزیں باہر کی چیزیں
نہیں کھاتی) اب تو Avoid کرنا ہے آپکو ان
چیزوں سے تاکہ تم کا جو Problem ہے وہ
گھٹائیں آپ مصالحے مرچیں Avoid کریں کم
لوگوں کی عادت ہوتی ہے کہ وہ باہر کا کی زیادہ
پسند کرتے ہیں تو ہم بھی ہم انکو کہتے ہیں کہ انکو
کم اس طرح سے کرو نا کہ Late night میں
نہیں کریں آپ اگر کر رہے ہو تو ہفتے
میں ایک دفعہ کرتے ہیں تو اس کا یہ ہے
مطلب ہے 6-9 بجے تک Normal meal
پہلے ہیں اور اس کے بعد اب Walk
کرتے ہو اور ہاں اس کے بعد اب Cold drink
نہیں لو تو زیادہ اچھا ہے کیونکہ اگر چائے
اس کے بالنسبت ساری چیزوں کو ہماری
چکناہٹ کو فضلے کے ذریعے نکل جاتا ہے
کیونکہ اگر ہم لوگ Spicy چیز تو چکنی
لے رہے ہیں اور پر سے Cold drink لے رہے
ہیں وہ ٹھنڈی ہے تو ہمیں تو جیسے اگر
For Example اگر kitchen ہے کام کرتے ہیں
تو اگر کبھی تو Pipeline میں جم جاتا ہے
گرم پانی ڈالتے ہیں تو سب نکل جاتا ہے
تو اسی طرح سے ہمارا بھی Intestine کا System
ہے کہ ہمیں چکناہٹ کے بعد گرم چیزیں
لیں جیسے Coffee چائے تو وہ زیادہ
بہتر ہے تو میں اکثر ایسا ہی کہہ دیتا ہوں
Patient سے

I اس کے علاوہ Patient کے علاوہ کوئی [illegible]
آپ سے پوچھتا ہے کہ غذائی کے بارے
میں بتائیں کیونکہ آپ Medical Field
سے ہیں۔ کوئی خاندان میں کبھی بھی
کوئی پوچھتا ہے آپ سے کبھی ہوا ہے اسطرح
سے

A کبھی ایسے کہتے ہیں کہ میں بہت [illegible] ہوں

Date ______

تو میں کیا کروں تو مجھے چاہیئے کہ مجھے تو اچھا Health
ہو جاؤں تو مجھ کتنا شوق کہ آلو اور چاول
کا زیادہ استعمال نہ کروں اسطرح ہوتا ہے

T ٹھیک کبھی ایسا بھی ہوا ہے کہ آپ نے
Community People کو Groups of [illegible]
میں جا کر غذا کے بارے میں کچھ بتایا ہو

A نہیں ایسا نہیں ہوا

T ایسا نہیں ہوا ٹھیک ایکو کیا لگتا ہے غذا کی
صحت دیتے وقت یا [illegible] Nutrition دیتے وقت
[illegible] کیا کیا پیش آتے ہیں یاں کوئی
challenge کا سامنا ہوا کوئی مشکلات
کا سامنا ہوا

A ویسے ایسا ہوا نہیں کبھی

T اچھا آپ جو سمجھاتے ہیں Patient آرام سے
سمجھ لیتا ہے یا بعض دفعہ ایسے سوال پوچھتا
ہے جس کا آپکے پاس جواب آ رہا ہو نہ آ رہا ہو
اسطرح سے کچھ

A نہیں ایسا بھی کبھی نہیں ہوا

T اچھا ٹھیک تو آپ کے خیال میں غذا کی صحت
پہ بات اگر ہم کریں تو کون زیادہ اچھی
بات کر سکتا ہے

A جیسے ایک Previous سوال کا جواب دے
رہا ہوں میں جیسے کسی کو Diarrhea وغیرہ ہوتا
ہے۔ مطلب یہ ضرور کہتے ہیں کہ تھوڑا Lightly
آپ نے تو یہ ہے مگر میں بھی [illegible] Lightly لے
تھوڑا سا آہستہ آہستہ جیسے ایک Diarrhea
ٹھیک ہونا شروع ہو جائے پھر آپ Regular
Diet آہستہ آہستہ Start کرو تاکہ [illegible]
Soft بھی ہوتا ہے تو Regular کی جگہ used to
Diet بھی لیتے ہیں کہ تو ایک Time لگا
ہے کہ پیٹ تنگ آ جاتا ہے تو بہتر یہی ہے نا
کہ آپ تھوڑا اسطرح نہ Hard ہے کہ پھر
Diet Normally پہ آ جاؤ جیسے Bread کیسا تھ بھی

Date ________

اگر آپ کبھی روٹی پہ آتے ہو تو روٹی یا نان جیسے بیکن نان
ایکدم نہیں لینا ہے کیونکہ نان Heavy ہوتا ہے۔
ٹھیک تو جیسے آپ کہتے ہیں Light لو تو Light سے T
Patient پوچھتا ہوگا کیا مراد ہے
Hmmm Light میں جیسے یہ کھچڑی ہوگیا دلیہ A
ثابت دانہ اس طرح کی چیزیں ہم دے سکتے ہیں
جن کا استعمال رکھو
ٹھیک، ٹھیک، آپ کے خیال میں غذائی صحت کے T
مسائل ہیں ہمارے پاس ہمارے پاکستان میں ہمارے
Environment میں اس طرح کے ہیں مسائل؟ کوئی
challenges ہیں اگر Hospital کے باہر بھی
بات کریں
یہ تو اگر Hospital سے باہر جائیں Community میں A
[illegible] ہے تو یہ تو کافی Area میں ہر جگہیں ہیں
اور ہیں بھی کیونکہ کچھ لوگ ایسے ہوتے ہیں کہ
Afford بھی نہیں کر پاتے آپ ایسا ابھی بھی ہے
2024 میں بھی اس طرح کے بھی ہیں۔
کچھ لوگ ایسے بھی ہیں جو 48 hrs میں کھانا
کھا رہے ہیں صرف 1 اور یہ تو Real
میں ہے کیونکہ مجھ سے ایک نام تو نہیں لینا
چاہیے تو مجھے 3-2 دن پہلے Share
کیا کہ 48hrs ہو گئے ہیں اور ہم لوگوں نے کچھ
کھانا وغیرہ کوئی Meal وائیٹ کی ہے اس کے
بعد ابھی تک نہیں کچھ بھی ہوتا ہے ایسے کہ میں
سمجھ رہا ہوں کہ یہاں ہونا چاہیے تو میں بھی help کر دیتی ہوں
اور بعض اوقات
یعنی کہ آپ کے کہنے کا مقصد یہ ہے کہ ہم لوگوں T
کی اس طریقے سے مدد کر سکتے ہیں لوگوں کو
جو afford نہیں کر سکتے وہ ان کو جو ہم کھانے
پینے کی چیزیں دیں یہ کیا چاہ رہے ہیں
ہاں یہ ہونا چاہیے مطلب اگر وہ Patient A
بھی ہے تو ہمیں نہیں پتا کہ ان کے گھر میں Patient
ہے یا نہیں ہے تو وہ خود بھی نہیں کھا پا رہا تو۔

Date

Patient کو یا اپنے گھر پہ کیسے دے سکتے ہیں یا
Bedridden Patient ہیں یا فرد ہے ایسا انکو
کیسے کر پائیں گے

I یعنی کوئی ایسی Service ہونی چاہیے کہ جس
میں جو مریض گھر کے اندر گئے ہیں وہ
bedridden ہیں اور نہیں treat کر سکتے نہیں
کھا سکتے نہیں رکھا سکتے یا کوئی بھی مجبوری ہے
تو اُن کو ایسی جگہ ہے یا [illegible] Hospital ؟

A یہ Hospital site

I یہ Hospital site ایسی ہو جو انکو ...

A provide treatment کر سکے

I اور انکو گھر کے اندر انکو کھانے پینے کی چیزیں
دے تاکہ انکی صحت بہتر ہو

A کیونکہ کچھ لوگ ایسے بھی ہوتے ہیں کہ وہ ہوتے
تو تندرست ہی ہیں Hospital کی need
نہیں ہے لیکن یہ کہ صرف treatment کی
وجہ سے وہ weak ہوتے ہوتے نہ ملنا
یہ بیماری چیزیں بھی ہیں

I تو آپ کے خیال میں واحد یہی وجہ ہے کہ
مہنگائی ہے یا اور بھی وجوہات ہو سکتی ہیں

A اگر مہنگائی کو دیکھا جائے تو یہ بھی وجوہات کچھ
سکتے ہیں

I اور کیا وجوہات ہو سکتی ہیں Nutrition care میں
proper نہ ہونے کی غذائی صحت Proper نہ ہونے
کی

A لوگوں کے پاس روزگار کا نہ ہونا Human [illegible]

I صحیح

A یہ بھی ہے Main Issue اور اگر ہے بھی تو ایک دو
فرد ہوتے ہیں باقی سارے بیچارے کھانے والے ہوتے
ہیں Earning اگر ایک آدمی ہے تو [illegible]
تو کر رہے ہیں اگر انکا بچہ چھوٹا ہے تو سمجھ لیں
آجکل تو لوگ 6، 7 سال تک کے بچے کو بھی دکان
پہ بٹھا دیتے ہیں تاکہ وہ کچھ کر کے آئے گا کام کرے

Date

گا کچھ ایسے بھی لوگ ہیں جو بیچارے سارا دن کام کر
رہے ہوتے ہیں تو رات میں ملے گا تو امید کرتے ہیں کہ
3-4 بجے یا جیسے گھر جائیں گے تو ان کو یہ ہوتا ہے
یہ امید ہوتی ہے کہ میرے پاس کم 100، 200، 300
روپے ہوں گے تو میں کچھ کھانا لے کر جاؤں گا۔

T ٹھیک

A بالکل Basically یہ روزگار اور یہ diet دونوں ہی
جڑ رہی ہیں

I Hmm

A اگر روزگار ہوگا تو ہر کوئی اپنے گھر میں
بھلے تھوڑا دے گا لیکن کچھ لے کر تو شام میں
جائے گا

T ٹھیک، آپ کے خیال میں یہ غذائی بحث
کی بات کون زیادہ بہتر طریقے سے کر سکتا ہے
لوگوں تک کون زیادہ بہتر طریقے سے پہنچا
سکتا ہے یہ بات ڈاکٹر یا کوئی ایسا بندہ
کہ جس کا background ہو nutrition میں پڑھا
ہوا ہو

A اگر تو دیکھا جائے تو کہتے ہیں نہ تو
First of all تو ڈاکٹر جانے سے پہلے لوگوں کا صبر
ان کے پاس Time نہیں ہے ٹھیک ہے اگر چلا بھی جائے گا
تو اس نے صرف آدھا ایک گھنٹہ لوگوں کو دیکھ
کر باقی وہ فیملی Health professional مطلب یہ کہ Professionally
تو کم Doctors ایسے بھی ہوتے ہیں جو Community میں
جا کر بھی حقارت کرتا تو یہ چیزیں Possible نہیں
ہوتیں آپ اس Field میں آئے ہیں تو آپ اگر
Field میں جا رہے ہیں تو لوگوں کو understand
کرنا پڑتا ہے

I Hmm صحیح کہہ رہے ہیں

A تو ان کو understand کرنا پڑتا ہے لوگوں کو اس طرح
ہم کر رہے ہیں تو ہم ان کو کیا دیکھ رہے ہیں تو پہلے تو ہم
ان سے الٹا نفرت کر رہے ہیں نہ تو وہ ہم سے بات کون
کرے گا اگر آپ ان کا لحاظ کر رہے ہیں تو وہ بھی ہاتھ ملائے

CamScanner

Date ______

ہیں بڑے بھی ہاتھ لگاتے ہیں ہمارا کام بھی Hand Therapy
کر کے انکو Feed کرنا ہے انکو اپنائیت دے
ہو وہ کھل کے بات کر سکیں بتا سکیں باتیں اور اگر
میں سمجھتا ہوں Nutrition کے حوالے سے Dietitian
اور Community Nurse وہ دونوں چیزیں بہت اچھے سے
کر سکتے ہیں Community Nurse ساتھ Dietitian جیسے
رکھتے ہیں بہتر جانتے ہیں اچھے سے کیا ہے complete Course Best knowledge
ہے انکو وہ انکے ساتھ ہیں ہم (Seminar Organize
کر لیں Community میں تو ایک جگہ پہ Arrange
کر لیں جیسے ہم یہاں جگہ پہ Attend کر نشن کا لوگوں
کو [illegible] Awareness ہو جائے) لوگوں کو بتایا جاتا ہے
چلیں کچھ انکو پاس سے کچھ دے نہیں سکتے Normally
تھوڑا بہت بتا تو سکتے ہیں کہ یہ چیزیں لیں یہ ہیں
اور اگر کرنا چاہیں تو وہ ہیں [illegible] روپے کی دلیہ ثابت دانہ
بھی پڑیا ملتی ہے وہ بن جاتا ہے۔

T کس چیز کی پڑیا

A ہے دلیہ ثابت دانہ ہمارے پاس ہوتا ہے تو وہ
بنا کے دے دیتا ہے جیسے کہ میں نے بتایا تھا اگر گھر
میں [illegible] Pulse ہے تو اور کچھ نہیں تو
انکو کھلانے کے کام آئے گا بچے وغیرہ مانگتے ہیں (کسی time)
Demand کرتے ہیں اور کچھ نہیں تو دلیہ کو کسی
بچے کو کھلانے کے کام آ جائے گا

T صحیح کہہ رہے ہیں بالکل صحیح کہہ رہے ہیں

A اس طرح سے وہ چیزیں ہو جاتی ہیں اگر آپ بہت
ہمارے ہم نہیں کر سکتے تو چلیں یہ چھوٹی چھوٹی
contribution تو ہم کر سکتے ہیں آپس میں

T آپ کا کہنے کا مطلب ہے کہ Dietitian یا
community nurse زیادہ بہتر طریقے سے یہ کام
کر سکتی ہے

A یہ [illegible] کر سکتی ہے

T صحیح ہے جو Community Nurse جو ہمارے پاس
ہوتی ہیں انکی اتنی knowledge ہوتی ہے
Nutrition کے بارے میں

CamScanner

Date ________

A نہیں میں نے کہا کہ دونوں مل کر کر سکتی ہیں

I دونوں مل کر کیا وہ اتنا کر Trained کر لیں پہلے

A نہیں جیسے میں Community Nurse ہوں، آپ Dietitian ہیں
ہم نے organize کیا لوگوں کو بتایا وہ آتے ہیں
ان میں سے اپنا کام ہے جیسے ہمارے پاس Doctor ہیں
ہیں Dietitian ہیں وہ ان کو اچھے سے بتا دیں گے
Diet کے حوالے سے

I صحیح

A تو اس طرح مل کر ہو گیا دونوں کا (جو چیزیں)
مجھے پتا ہیں وہ میں نے بتا دیں جو مجھے نہیں پتا وہ
میں نے آپ سے پوچھ لیا اور آپ لوگ Dietitian
کا پتہ جانتے ہیں تو Nurse Dietitian اتنا نہیں جانتے
تو اس طرح سے بجائے ہم کہیں گے یہ وہ [illegible]
Guide کرنے سے بہتر ہے بتا کر میں پہلے مجھے
پتا ہے لیکن مجھے پتا ہے کہ Diet آپ بتا
سکتی ہیں تو میں آپ کی طرف کیوں نہ Direct
refer کر دوں

I اچھا صحیح

A یہ چیز ہوتی ہے اس طرح سے مل کر بھی ہو جاتا
ہے

I صحیح بالکل صحیح، دوسرا میں یہ پوچھنا چاہ رہی
تھی آپ نے ابھی تک تو ہم health کے حوالے
سے بات کی تو اگر ہم overall دیکھیں تو آپ کے
خیال سے ہم ایسا کونسا طریقہ اپنا سکتے ہیں جس
سے جو ہے غذائی محنت بہتر تو تمام عوام کی ہم
سب کا ایسا کیا ہم چیزیں کریں کہ ہم کیا کریں کہ ہم
کم جس سے آپ کو لگتا ہے کہ اگر یہ ہم کام کریں
گے تو بہتر ہو جائے گا چیزیں بہتری کی طرف جائیں گی

A میں تو سمجھتی ہوں اس حوالے سے اگر سب
سے بڑھ کر مہنگائی ہے اگر یہ بھی کم ہو جاتی ہے تو
بہتری ہو گی [illegible] چیز تو نہیں لیتے ساری چیزیں
مہنگی ہیں جس نے جو لینا ہوتا ہے وہ بھی نہیں لے پاتے
جو لے پاتے ہیں تو ظاہر ہے جہاں تو ہم ایک گھر کی Income تھی

Date ________

دیا ہے 2030 کے لحاظ سے کم گزارہ کر رہے ہیں

I: یعنی کہ آپ کے کہنے کا مقصد ہے کہ اگر مہنگائی کم ہو جائے تو لوگوں کی صحت سب کی صحت بہت اچھی ہو جائے گی. یہ کہنا چاہ رہے ہیں آپ

A: نہیں صحت ...

I: خوراک کے لحاظ سے

A: خوراک کے لحاظ سے تو میں کہوں گا کہ کم کیسے ہیں اس میں ہو سکتا ہے لیکن اگر صحت کو کہوں مطلب یہ کہ اس میں تو ہمیں یہ ہم چیزیں دیکھنی ہیں کہ جیسے صفائی ستھرائی کہ جیسے دیکھیں بارش ہوتی ہے یہاں سے بچے پڑھنے گزرتے ہیں پانی سے بھی علاقوں میں دیکھیں کتنی گندگی ہو جاتی ہے صفائی ستھرائی ہونی چاہیے تو ان چیزوں سے پھر infection اور بیماریاں پھیلتی ہیں

I: یہ آپ نے بہت اچھی بات کی کہ صفائی ستھرائی کے لحاظ سے یہ چیزیں ہونی چاہیے لیکن یہ جس طرح غذائی صحت جس طرح ہوتی ہے نا کہ بہت سارے آپ نے ذکر کیا تھا کہ بہت سے ایسے بھی ہوتے ہیں کہ جن کو صرف غذائی چیزیں نہیں Food نہیں مل رہی ہوتیں ہیں کہ جن کی وجہ سے ہے

A: مہنگائی ہے تعلقہ Related میں نے اس کا بھی Answer دیا تھا

I: جی آپ نے کہا تھا مہنگائی سے مسئلہ اور جیسے یہ پیسے والے لوگ ہیں ان کے لیے مہنگائی کا کوئی مسئلہ نہیں ہے آپ کے خیال میں وہ سب صحیح غذا کھا رہے ہیں

A: جن کے پاس پیسے ہیں وہ تو صحیح یہ کھاتے ہیں اسلئے کہ اگر دیکھا جائے کہ کسی کے گھر دیا جائے یہ اگر ادھر Truck بھی آ جائے گا تو سب سے پہلے گاڑی والے لائن میں جائیں گے کہ ہم لوگ بھی سب سے زیادہ غریب.

Date

ہیں تو ایسا Previous کچھ 2 Year میں
تا یہاں پر Nazimabad میں ہوا تھا کہ میں
نے بنوایا ہوا تو دو Truck آتے تھے آٹا انکو
دینے کیلئے لوگوں کیلئے گھر سے بیٹھے ہیں سے کم
لوگ آتے تھے بہت غریب طبقے کے وہ بھی آتے
ہمارے کافی لوگ Line میں بھی لگے ہوتے
تھے اور کچھ لوگ جو تھے وہ گاڑی Main
Road پہ ٹھہر کے Line میں لگ
ہوئے اور آس پاس کے جو Banglow ہیں
وہ بھی Line میں لگے ہوئے ہیں کہ ہم
سے زیادہ کوئی غریب ہے ہی نہیں

I Hmm...

A تو ہم جو Distributor ہوتے ہیں میری
PR آپ سے ہے میں ہم دور سے دیکھ کر
تو اشارہ کر دو گا تو ہم میں نے آپ
لوگوں کو وہ کر دیا تو دوسرے تو ہم
دوسرے تو بیچارے وہیں رہ گئے نا۔

I صحیح۔ اور کچھ ہمارے ساتھ کچھ Hmm.
بات Share کرنا چاہیں گے ہمارے ساتھ
nutrition care کے حوالے سے مطلب
Generally اگر کچھ بھی Share کرنا چاہیں

A میں تو یہی کہوں گا کہ اگر ہمیں اللہ
نے اس قابل کیا ہے تو کسی کو Nutrition
کے حوالے سے اگر تو ہم Pay کرتے ہیں
Pay تو ہم کوئی کرتا ہے اگر ہم نہیں
کھانا ہی دے دیں تو زیادہ اچھا ہے

I صحیح۔

A اگر جیسے باہر گھر کا اسلئے کہہ رہا ہوں کہ
میرے دل میں ہے اگر میری کوشش ہوتی
ہے جیسے Karachi کے سامنے بھی لوگ
ہیں کافی لوگ ہیں تو اگر میرے پاس
ہے تو میں تھوڑا بہت یہ کر کے دیکھ لیکن
ہم بھی جتنا ہم سے ہوتا ہے اتنا میں کرتا ہوں۔

Date

Thank you , God bless you

Thank you okay

x———x

CamScanner
