# Supplementary material for: Qualitative exploration of comprehension and experiences of healthcare professionals regarding nutrition care in Karachi, Pakistan
Source: PLOS Glob Public Health. 2025 Dec 30;5(12):e0005483. doi: 10.1371/journal.pgph.0005483 (PMC12753000; doi:10.1371/journal.pgph.0005483)
Supplement: S5 File — (ZIP) [file pgph.0005483.s005.zip › Nurse Male-007.pdf]

Date [redacted] Nurse Male-007

I اسلام علیکم، اچھا ہم Research work کر رہے ہیں
جس کے اندر ہم different doctors & nurses سے ان کا
concept پوچھ رہے ہیں کیا ہیں ان کے opinion
nutrition کے حوالے سے یا غذائی صحت کے حوالے
سے تو اس سلسلے میں ہم نے آپ کو بلایا ہے یہاں پر
تو آپ اپنا نام بتائیں گے

S [redacted]

I آپ سے اجازت لے رہے ہیں کہ اگر آپ اس
چیز کی اجازت دیتے ہیں تو ہم آگے Proceed
کرتے ہیں تو آپ [illegible] اس پر Sign
کر دیں یہ consent form ہے۔ اگر آپ کسی
سوال کا جواب نہ دینا چاہیں تو آپ ہمیں
بتا دیجیے گا اس سے ہمارے interview
پہ کوئی فرق نہیں پڑے گا، اپنا نام وغیرہ
جتنا بھی آپ بتائیں گے تو وہ کوئی چیز
نہیں آئے گی

S نہیں کوئی issue نہیں ٹھیک ہے
It's okay no problem

I اچھا آپ تھوڑا اپنے بارے میں بتائیں

S جی میرا نام شاھد انور ہے میں نے RN کیا
ہے اور specialty بھی میں نے کی ہوئی ہے
[illegible] میں بھی کوئی تقریباً ایک ڈیڑھ سال
ہو گیا ہے، اس سے پہلے میں علاقے میں
hospital تھا وہاں میں Job کرتا تھا اور
home care nursing بھی میں نے کی ہوئی ہے اور آجکل
تو [illegible] بھی کر رہے ہیں اور ساتھ میں study
بھی شروع کیا ہے

I اچھا ٹھیک ٹھیک کیا پڑھ رہے ہیں آپ

S Post Doc میں

I ٹھیک ہے very good بہت اچھی بات ہے
تو آپ کے خیال سے کیا ہے غذائی صحت ہے
کیا آپ کے دماغ میں کیا چیز آتی ہے پہلے
یہ بات سن کے۔

S دیکھیں سب سے پہلے تو nutrition کی بات کریں
غذائیت کی بات کریں تو اس میں
healthy food لینا چاہیئے میں تو اکثر بچوں
کو کہتا ہوں کہ آپ junk food avoid کریں fruits
کی طرف آئیں vegetables کی طرف آئیں اور
دودھ پئیں juice پئیں لیکن ان کو تو بس
Fast food ہی چاہیئے chocolate اور یہ اور
میں تو ان کو اکثر کہتا ہوں کہ آپ chocolate
کی جگہ آپ کھجور استعمال کر لیں لیکن
وہ تو نہ ہونے کے برابر ہے بڑی مشکل سے
دودھ بھی وہ بڑی مشکل سے پیتے ہیں بس
ان کو لولئے رہتے ہیں۔ اور fruit بھی آپ
یقین کریں ایسے کھائیں گے کہ plate
ایک تیار کر کے دے دیں تو کھائیں گے ورنہ نہیں
تو میں یہ سمجھتا ہوں کہ بہت ضروری ہے کہ ہم لوگ
healthy food کھائیں [illegible] کھائیں fast food
کو avoid کریں جو کہ ہماری young generation
تو بالکل اس طرف نہیں آتی ان کو fast food ہی
چاہیئے اور خاص طور پہ مطلب میری Age جو
ہے 37% ہے میں تو cold drink بالکل ہی
avoid کرتا ہوں میں چاہتا ہوں کہ بچے بھی
ہیں وہ avoid کرتا ہوں cold drink بھی میں یہ
suggest کرتا ہوں کہ ہمیں نہیں لینی چاہیئے اگر
ہم لوگ اس سے دور رہیں تو بہت اچھا ہے
کیونکہ اس میں بہت زیادہ Sugar ہوتی ہے
تو یہ تو نقصان دہ ہے ہمارے لئے اور [illegible]
زیادہ deep fried چیزوں سے ہم بھی دور
رہیں تو وہ بھی اچھا ہے ہمارے لیئے اور
healthy food کھائیں صاف ستھرا food کھائیں
ہر چیز fruit سے vegetables سے اس کو ہم wash
کر کے کھائیں اس چیز کا بھی میں خاص خیال
رکھتا ہوں اور باقی [illegible] plan بنائے ہیں کہ یہ کھائیں
گے یہ نہیں لیکن life اتنی busy ہوتی ہے

کہ اس schedule کو کرنا مشکل ہو جاتا ہے۔ جیسے میں
نے اپنا meal plan بنایا تھا کہ میں صبح کو brown
bread کھاؤں گا دوپہر میں چپاتی کھاؤں گا
لیکن کبھی کبھی تھک جاتے ہیں کہ بغیر ناشتے کے ہی
hospital move ہونا پڑتا ہے۔ اور دوسرا یہ ہے کہ
اگر healthy رہنا ہے تو exercise
ضروری ہے 24 گھنٹے میں ہم half hour یا
hour walk کریں تو وہ بہت اچھا ہے اپنے لیے

I: Patients سے کبھی interaction ہوا ہے
یا کسی بھی individual سے

S: یہ nutrition کے حوالے سے؟

I: جی nutrition کے حوالے سے nutrition care
کے حوالے سے غذائی صحت کے حوالے سے

S: زیادہ تو ہمارا interaction نہیں ہوتا ہم لوگ
تو آپ کو پتا ہے medicine کرتے ہیں اور جو
patient کے کام جو ہیں room سے باہر آکر
کرتے ہیں۔ اگر کوئی زیادہ ہمیں force کرتا
ہے کہ بتائیں ہم لوگ کہ stomach میرا خراب ہے
کیا کریں میرے میں جلن رہتی ہے تو ہم
یہی کہتے ہیں کہ آپ یہ spicy چیزیں avoid
کریں liquid diet لیں actually دلیہ میں
آئیں تو آپ کا stomach set ہو جائے گا
بہت کم لوگ زیادہ تو doctors ہی mainly question
Patient کرتے ہیں آپ کو پتہ ہی ہے answering
سے تو ہم سے بہت کم contact کرتے ہیں
اگر کوئی پوچھتا ہے تو ان کو یہ suggestion کرتے
ہیں کہ

I: ویسے خاندان میں یا ویسے کوئی پوچھتا ہے
nutrition care کے حوالے سے

S: جی جی بالکل وہ تو بالکل جیسے ہی ہمیں پتا ہے ہیں
کہتے ہیں یہ تو medicine سے ہے تو فوراً پوچھتے
ہیں اچھا جو یہ پیٹ خراب ہے کیا کھاؤں
میں پوچھتا ہوں کہ کیا کھایا ہے تو وہ بولتے ہیں باہر سے

Date ۵۰۹

کھا رہا ہوں میں نے کہا وہ بند کر دیں گھر کا کھانا کھائیں اور
spicy چیزیں avoid کر دیں گھر کا کھانا کھائیں
اور بہتری کی طرف آپ جائیں گے۔ زیادہ تر میں نے
یہ دیکھا ہے خود پہ بھی میں نے یہ experiment کیا ہے
continue اگر باہر کا کھانا کھاؤ تو میری
health خراب ہونا شروع ہو جاتی ہے میں
دوسروں کو بھی یہی suggest کرتا ہوں باہر کا کم
کھائیں اگر زیادہ کوئی مجبوری ہے otherwise
avoid کر دیں پڑھتے ہیں رشتہ دار دوست
وغیرہ انکو پتہ بھی ہے میں health conscious ہوں
ہم فون کر لیتے ہیں یا ملاقات پہ پوچھ لیتے ہیں
بات یہ ہے suggestions مانگتے ہیں
آپ کو لگتا ہے عمل ہم کرتے ہیں یہ نہیں کرتے آ
کرتے ہیں ہم ٹھیک ہو جاتے ہیں ہم دوسری S
طرف چلے جاتے ہیں
ٹھیک آ
رکھتے پرہیز داری نہیں کرتے وہ S
جیسے آپ کہہ رہے ہیں Regular آ
follow Regularly نہیں کرتے ہماری Society S
میں بہت مشکل ہے ایک چیز کو Start
کرتے ہیں اسکو continue کرنا بہت مشکل
ہو جاتا)
تو اسکی وجوہات کیا ہو سکتی ہیں آپ کے خیال میں آ
میرے خیال میں تو اول اگر اپنی life پہ بولوں S
میں کوشش کرتا ہوں میں exercise کروں لیکن
کبھی موقع مل جاتا ہے کبھی نہیں ملتا (عام لوگ
[illegible] ہم کہیں یہاں سے جائیں بہت کم ہوتا
ہے کرتے میرے خیال میں بیچ میں آ جاتا ہے
career busy life ہونے کی وجوہات
ہوتی ہیں
ٹھیک کبھی آپ کو موقع ملا nutrition counselling آ
کے حوالے سے زیادہ افراد سے بات کرنے کا
community میں زیادہ افراد سے ایک ساتھ

Date ________

groups سے

S نہیں نہیں ایسا تو کچھ نہیں

I آپ کے خیال میں غذائی صحت دینے میں یا Provide کرنے میں کیا کیا Challenges ہو سکتے ہیں کیا کیا مسائل درپیش ہو سکتے ہیں

S میرے خیال میں میرا نظریہ یہی ہے کہ جیسے ہم لوگ community میں جا کر کام کر لیں session launch کریں لوگ بیٹھیں اس میں وہ لوگ ہیں جو اس میں کم لیں گے جو اس پہ عمل نہیں کریں گے تو یہ چیز آتی ہے ہماری community میں society میں دیکھا جائے تو جو لوگ کر لیتے ہیں وہ اپنی lifestyle کو بہتر دیکھتے ہیں کہ میں healthy feel کر رہا ہوں تو اگر اس چیز کا احساس ہو جاتا ہے کہ ہمیں اچھی nutrition کی طرف health کی طرف آنا چاہئے اور جو لوگ نہیں کرتے تو پھر وہ بیمار رہتے ہیں اب health بھی خراب ہے تو یہ کرنا چاہئے awareness تو آجکل ویسے بھی ہے

I تو اس کو solve کیسے کر سکتے ہیں یہ جو آپ نے بتایا challenge

S تو میں جیسے میں سمجھتی ہوں آجکل social media بہت active ہے تو اس میں ہمیں کرنی چاہئے اس طرح کی

I کونسے social media platform پہ کی لوگ کیا زیادہ دیکھتے ہیں آپ کے خیال میں

S زیادہ تو facebook ہے insta اور whatsapp بھی use کرتے ہیں اور بھی یہ ہے twitter ہو گیا یہ لوگ زیادہ youngster ہمارے جو ہیں یعنی جو آنے والی نسل ہے جو آگے کمان سنبھالنی ہے اس چیز کو follow کرتے ہیں میرے نظریے میں اگر لوگ use کریں تو social media کا platform وہ best رہے گا

Date ________

I ٹھیک تو اسمیں کیا کیا جا سکتا ہے social media پہ

S اسمیں جیسے کہ اب video upload کر دی Lemon
چلتی ہے Lemon لگا دی ہم لوگوں نے یا
Tweet کا status لگا دیا اس طرح سے میں سمجھتا ہوں
یہ چیز اچھا benefit ہو گئی ہمارے لیے غذائی
صحت کے حوالے سے

I ٹھیک ہے جیسے ایک آدھ message آپ
مثلاً بتائیں کیا ہونا چاہیے

S جیسے اب message یہ ہو گا کہ fast food
avoid کریں، vegetables کھائیں fruit
کھائیں dry fruit کھائیں nuts کھائیں
healthy food کھائیں cold drink کو avoid کریں
کھائیں دودھ کا استعمال کریں یہ ہمیں
message دینے چاہئیں

I صحیح اس کے علاوہ آپ کچھ add کرنا چاہیں
گے اسمیں nutrition care کے حوالے سے غذائی صحت
کے حوالے سے اور کیا ہونا چاہیے

S Add بھی کرنا چاہوں گا کہ اگر ہم لوگ تھوڑا
add کر لیں فروٹس ہیں اسکو دھو کر کھائیں
vegetables ہیں تو اسکو دھو کر کھائیں اور اگر ہم
لوگ کہیں سے کھانے کی چیز لیں تو اسکو دیکھ
لیں یہ اچھی quality کی ہے یا کہ نہیں
quality کی quality بھی ہمیں دیکھنی چاہیے
بعض دفعہ food اچھا نہیں ہوتا تو بیمار ہو
جاتے ہیں گھر والے کہتے ہیں کہاں سے food لے آئے
ہیں کئی دفعہ گھر والوں نے بولا ہے کہ اس
دفعہ کا food اچھا نہیں لائے میں کہتا ہوں
اچھا اگلی دفعہ change کر لوں گا تو یہ بھی
awareness ہمیں پھیلانے کی quality کی بھی
ہونی چاہیے

I آپ کے خیال میں nutrition پہ بات کس کو
کرنی چاہیے doctor کو کرنی چاہیے یا
کسی ایسے بندے کو کرنی چاہیے جس کے nutrition

Date

کا background ہے جس کے پڑھا ہوا ہو اپنے پورے
سالوں میں medication کسی کو بات کرنی چاہیے
کسی کے لوگ زیادہ نہیں ہیں

S: میرے خیال میں تو جیسے today کہ آپ first
priority دے رہے ہیں جیسے میں medical سے
Any medical سے نہیں تو nursing ہو
کوئی بھی related ہو Pre-medical
کا staff ہے تو اسی لوگ بیٹھے ہیں
swallow کرتے ہیں یہ تو ضروری ہے کہ
ہم لوگ اپنی community کو تھوڑا aware
کریں breastfeeding کیلئے

I: ٹھیک ہے اور کچھ آپ add کرنا چاہیں گے
ویسے کافی اچھی باتیں آپ نے کی ہیں

S: I think نہیں جی بہت شکریہ knowledge
بہت ہے

I: ٹھیک ہے Thank you so much آپ
کے time کا بہت شکریہ
