# Supplementary material for: Qualitative exploration of comprehension and experiences of healthcare professionals regarding nutrition care in Karachi, Pakistan
Source: PLOS Glob Public Health. 2025 Dec 30;5(12):e0005483. doi: 10.1371/journal.pgph.0005483 (PMC12753000; doi:10.1371/journal.pgph.0005483)
Supplement: Attachment — Submitted filename: Response to Reviewers.docx [file pgph.0005483.s009.docx]

**Rebuttal letter**

**PGPH-D-25-01594**

Qualitative exploration of comprehension and experiences of healthcare professionals regarding nutrition care in Karachi, Pakistan

Dear Dr. Hawley,

We sincerely thank you and the reviewers for the thoughtful and constructive feedback on our manuscript. We have carefully revised the manuscript in line with the comments provided. Below, we address each comment point-by-point, indicating the changes made. All modifications are highlighted in the revised manuscript with track changes file.

Kind Regards,

Maria Atif

**Journal Requirements:**

**1. Please amend your online Financial Disclosure statement. If you did not receive any funding for this study, please simply state: “The authors received no specific funding for this work.”**

The online Financial Disclosure statement stated that the authors received no specific funding for this work.

**2. Please update your online Competing Interests statement. If you have no competing interests to declare, please state: “The authors have declared that no competing interests exist.”**

The online Competing Interests statement stated that “The authors have declared that no competing interests exist.”

**3. In this instance it seems there may be acceptable restrictions in place that prevent the public sharing of your minimal data. However, in line with our goal of ensuring long-term data availability to all interested researchers, PLOS’ Data Policy states that authors cannot be the sole named individuals responsible for ensuring data access (http://journals.plos.org/globalpublichealth/s/data-availability#loc-acceptable-data-sharing-methods).**

**Data requests to a non-author institutional point of contact, such as a data access or ethics committee, helps guarantee long term stability and availability of data. Providing interested researchers with a durable point of contact ensures data will be accessible even if an author changes email addresses, institutions, or becomes unavailable to answer requests.**

**Before we proceed with your manuscript, please also provide non-author contact information (phone/email/hyperlink) for a data access committee, ethics committee, or other institutional body to which data requests may be sent. If no institutional body is available to respond to requests for your minimal data, please consider if there any institutional representatives who did not collaborate in the study, and are not listed as authors on the manuscript, who would be able to hold the data and respond to external requests for data access? If so, please provide their contact information (i.e., email address). Please also provide details on how you will ensure persistent or long-term data storage and availability.**

The electronic copies of data are kept in a secure computer system, managed by the Ethical Review Committee of Ziauddin University and data can be accessible by requesting the ERC contact person, Dr. Nadia Haroon, at nadia.haroon@zu.edu.pk

We have provided the contact details of the ethics review committee to ensure long-term access.

**4. “S2 Checklist.docx” is currently uploaded as an 'Other' file type, which is not viewable by reviewers. Please ensure that all files meant for review are uploaded as 'Supporting Information' and include a legend in the manuscript.**

We apologize for the inconvenience. The S2 checklist document is revised and uploaded as supporting information.

**5. We have noticed that you have uploaded Supporting Information files, but you have not included a list of legends. Please add a full list of legends for your Supporting Information files before or after the references list.**

We apologize for the inconvenience. A list of legends has been added before the reference list

**6. If the reviewer comments include a recommendation to cite specific previously published works, please review and evaluate these publications to determine whether they are relevant and should be cited. There is no requirement to cite these works unless the editor has indicated otherwise.**

Only the relevant publication is cited.

**7. Please review your reference list to ensure that it is complete and correct. If you have cited papers that have been retracted, please include the rationale for doing so in the manuscript text, or remove these references and replace them with relevant current references. Any changes to the reference list should be mentioned in the rebuttal letter that accompanies your revised manuscript. If you need to cite a retracted article, indicate the article’s retracted status in the References list and also include a citation and full reference for the retraction notice.**

We have reviewed the reference list and added the citations. There is no retracted article cited.

| **No.** | **Reviewer 1 Comment** | **Response** |
| --- | --- | --- |
| 1 | The authors were silent on field notes, can they provide insights on how field notes were handled? | Thank you for your comment. Field notes were taken to capture nonverbal cues of participants, which helped in the analysis and interpretation of data. In cooperated in line 178-180. |
| 2 | During coding, how did authors resolve conflicts. | Coding was done independently by 2 authors. The coherence of coding was discussed and consensus was reached after involving 3^rd^ author. Still, if there was any ambiguity, the 4^th^ author was taken on board and a mutual consensus was achieved through discussion. |
| 3 | What type of qualitative coding did the authors perform on their transcripts, kindly be specific on which was used as it may also influence your results and interpretation. | Thank you for the comment. Deductive coding was performed with a **predefined set of codes** or categories based on the research question and objectives. |
| 4 | As part of their report, authors should indicate how their backgrounds and experiences influenced the interviews, their codes, their themes, the results of this study, and how these were handled. | Multi-disciplinary team was formulated for this research, with the PI belonging to the nutrition background field, along with an expert from Public health having vast experience in qualitative research to avoid influence on interviews, codes, themes or result. Multiple researchers were involved in coding and development of themes and consensus was maintained after discussion. |
| 5 | The authors should kindly report about their personal biases, assumptions, reasons and interests in the research topic. | Thank you for your suggestion. In order to avoid the biases and assumptions in this study, a multidisciplinary team including a Public Health PhD expert, along Clinical Nutritionist as well as a Public Health Nutritionist. All were aware of the inherited biases when the analysis was done. All the authors brought their expertise to understand the results and the discussion disagreements were resolved through discussion. |
| 6 | Authors indicated that “Data was first immersed by the NTS and SS”, but it is unclear how many people did the coding. Authors should also be friendly with non-technical readers by using terms that can generally be understood. | Coding was independently done by 2 authors, and in case of any discrepancy 3^rd^ author was involved and still if there was any ambiguity 4^th^ author was involved and consensus was achieved after discussion. |
| 7 | Authors should also consider structuring their work according to PLOS Global Public Health reporting guidelines. | Thank you for your suggestion. The work has been structured and highlighted in word file to in cooperate all suggestions and the manuscript is in accordance to PLOS Global Public Health reporting guidelines |
| 8 | Additional comments are embedded in the word file. | Thank you for your comment. All the additional comments are addressed and highlighted in the word file. |
| 9 | I suggest the authors should in brief describe how the framework was used enable readers appreciate what was done.  Attaching the framework as additional materials would be useful for readers. | Thank you for your comment. Comment addressed on lines 34-39. The framework was attached as supplementary material (S4 Table). |
| 10 | Is this the appropriate section for the sentence “We adhered to Lincoln and Guba’s criteria (18) for ‘trustworthiness’ by establishing credibility through open-ended questions, dedicating ample time to the data, and providing a comprehensive description of our methods.”? I suggest that it may better fit in the methods sections (data collection) instead of the data analysis section. | Thank you for your comment. Comment addressed on lines 168-171. |
| 11 | The interview guide can be attached as additional materials | Interview guide attached as Supplementary material (S3 Table 1). |
| 12 | How were the audio recorded?  How did the authors ensure confidentiality of the audio recordings? | Thank you for your comment. Comment addressed on lines 175-180. |
| 13 | Authors should kindly describe to improve clarity on how “they transcribed the text line by line for coding”. | Thank you for your comment. Comment addressed on lines 185-190. |
| 14 | The authors should kindly be brief on how thematic coding approach was employed in their analysis. | Thank you for your comment. Comment addressed on lines 194-196. |
| 15 | The authors should also include the number of eligible participants who declined to participate or were unable to complete the study, and provide potential reasons for their non-participation. | There were no participants who declined to participate in the study. All 15 participants willingly gave written consent. |

| **No.** | **Reviewer 2 Comment** | **Response** |
| --- | --- | --- |
| 1 | In the first sentence (lines 47–49), please provide a citation for the definition used. Is this sourced from a nutrition or dietetics manual, glossary, institutional document, or report? | We reviewed the definition, with the citation added in lines 52-54. |
| 2 | Page 4, line 65: When introducing the term ‘food environment,’ consider providing a brief definition and a supporting reference or example. This will clarify your meaning for readers, given that the introduction has focused on ‘nutrition’ until this point. Highlighting the importance of the food environment in the nutrition context would strengthen this section. | Thank you for your suggestion. We have added the definition with supporting reference and example in lines 69-74. |
| 3 | Page 4, lines 77–78: Please add a reference for the ‘study’ mentioned regarding midwives’ and nurses’ participation in nutrition care and their reported lack of knowledge and confidence. Also, consider clarifying whether you are referring to a specific study, multiple studies, or research in general, for precision. | Thank you. References were added and clarified in lines 83-84. |
| 4 | In the introduction, you discuss the connection between NCDs and nutrition. It would be helpful to provide examples of the types of NCDs to which you are referring. Since ‘NCD’ is mentioned throughout the manuscript without specific examples, consider clarifying which NCDs are most relevant to your discussion—especially when describing the double burden of malnutrition and NCDs (e.g., in lines 101–102 regarding Pakistan). This will help readers better understand the relationships between nutrition and specific diseases. | Thank you. Clarification of NCD with example and references added in lines 107-113. |
| 5 | In the methods section (lines 150–155), the way participant numbers are presented is unclear and could be confused with citation style. Consider using ‘(n=11)’ and ‘(n=5)’ for clarity. | Thank you. A clear presentation of participants number was added in a suggested way in lines 161 -166. |
| 6 | The use of authors’ acronyms (e.g., NTS and SS) is confusing, especially in the data analysis section. I recommend removing these acronyms and instead using a general description of the process (e.g., ‘We analysed data by…’). Stating which author performed each task may not be necessary and could be omitted for clarity. | Thank you, acronyms were removed, and a general description of the process was added in lines 183-188. |
| 7 | Page 9, line 169: When referencing Braun and Clarke’s work, please include the appropriate citation for their thematic analysis method. The current citation appears to be for COREQ, which relates to reporting guidelines, not the analysis method itself. | Thank you. Appropriate citation of Braun and Clarke’s work for the thematic analysis method and a little description was added in lines 191-194. |
| 8 | Results, page 13, lines 271–279: This section appears to address practitioners going beyond their responsibilities, rather than fitting within the private/public sub-theme. Providing additional context on why this content belongs in the sub-theme would be helpful. | Thank you. The mentioned section moved from private/public sub-theme to the theme going beyond their responsibilities in line 235-243. |
| 9 | Page 21, line 447: If appropriate for the local context and translation, consider replacing ‘man’ with a more inclusive term such as ‘lay-audience.’ Please disregard this suggestion if it does not suit the language or cultural context. | Thank you. ‘man’ word was replaced with ‘lay-audience’ in line 470. |
| 10 | Discussion, first sentence: If available, please cite a systematic review or other source to support the claim that this area is under-researched, as it forms a key basis for your study. | Thank you. Systematic reviews citation was added in line 490. |
| 11 | Lines 470–473: Currently, this section reads as though there are more than three themes. Ensure that the three themes match those described in the results (e.g., Beyond responsibility, Being compelled, and Disseminating). | Thank you. We revised this section, ensuring each sub-theme is supported by data extracts and better aligned with findings in lines 493-495. |
| 12 | Lines 474–475: Please clarify whether the ‘recent findings’ refer to your own study or to the broader literature. If referring to external research, include a citation. | Recent findings refer to our own study and are rewritten for clarity in lines 522-525. |
| 13 | Lines 499–507: To contextualize this important result, consider comparing it with a health system where this recommendation is implemented, or another setting where multidisciplinary approaches have been successful. Relating your findings to the broader literature would strengthen the discussion. | Thank you for your suggestion. We expanded the discussion and added comparisons with citations to strengthen the discussion in lines 525-531. |
| 14 | In your discussion, you highlight the importance of training. However, you also note that health practitioners are often overloaded and face constraints in providing nutritional support. It may be worth suggesting a systems-level change that involves integrating nutritionists more fully into patient care, rather than simply increasing training for existing practitioners. Training could focus on when and how to refer to nutritionists, ensuring these specialists are available within public health services to support a truly interdisciplinary approach. | Thank you for your suggestion. We expanded the discussion with citations to strengthen the discussion in lines 590-604. |
| 15 | In your ‘future research’ section, you note that some practitioners highlight gaps in the interaction between nutritionists/dietitians and patients. It may be valuable to explore the perspectives of nutrition professionals in future studies. Including this as a research need could help identify where gaps truly exist. | Thank you for your suggestion. We revised this section in lines 618-622. |
| 16 | p.4, line 76: remove the period before references (5)(6). | Comment addressed in lines 71. |
| 17 | p.7, line 143: use “(Table 1)” for clarity. | Comment addressed Table 1 was added as a supplementary material (S3 Table 1). |
| 18 | p.15, line 322, and p.16, line 348: capitalize “I.” | Comment addressed in lines 345 and 371. |
| 19 | p.21, line 552: “non-communicable disease (NCDs)” is already defined earlier; no need to repeat in full. | Comment addressed in line 580. |

**Reviewer 3**

**Comment:** This work addresses a gap in the literature on the challenges faced in developing countries, and in Pakistan in particular, in the delivery of nutritional care. The results presented are consistent with the announced title and the methodology presented. The manuscript is written in a readable and understandable manner. However, some clarifications could be made regarding the methodology. It is not clear whether the recruitment and the interviews were carried out simultaneously or in a staggered manner as suggested by the dates mentioned because of this sentence "The recruitment process started on 19th December 2023. Shortly after, participant interviews began and continued alongside recruitment. Recruitment ended on 19th February 2024, once initial signs of data saturation appeared". How did the data saturation appear before having collected data?

**Response:** We thank the reviewer for this observation. Recruitment was completed by February 19, 2024, while interviews continued until April, with data saturation identified in the later stages and confirmed through subsequent interviews. We have revised the methods section to clarify this sequence in line 156-160

**Reviewer 4**

**Comments**: The paper is well written. Additional information related to we paradigm that guided the study will and to the richness of the paper. Authors should also review punctuations around atactions esmee 75 seems to have a full stop before and after The citationsee unes 77 to 82 _ there's red to cold citations You have indicated in line 77 ' study show is it just one..cite which study or studies the statements that follow are poorly cited.

**Respond:** We thank the reviewer for this observation. We have added details on the interpretive paradigm guiding our qualitative approach and the punctuation and citation were corrected throughout the entire article.
